# Protein Disulfide Isomerase Inhibitor Suppresses Viral Replication and Production during Antibody-Dependent Enhancement of Dengue Virus Infection in Human Monocytic Cells

**DOI:** 10.3390/v11020155

**Published:** 2019-02-13

**Authors:** Nantapon Rawarak, Aroonroong Suttitheptumrong, Onrapak Reamtong, Kobporn Boonnak, Sa-nga Pattanakitsakul

**Affiliations:** 1 Division of Molecular Medicine, Research Department, Faculty of Medicine Siriraj Hospital, Mahidol University, Bangkok 10700, Thailand; n_1103@hotmail.com; 2 Graduate Program in Immunology, Department of Immunology, Faculty of Medicine Siriraj Hospital, Mahidol University, Bangkok 10700, Thailand; 3 Department of Molecular Tropical Medicine and Genetics, Faculty of Tropical Medicine, Mahidol University, Bangkok 10400, Thailand; onrapak.rea@mahidol.ac.th; 4 Department of Microbiology and Immunology, Faculty of Tropical Medicine, Mahidol University, Bangkok 10400, Thailand; kboonnak@gmail.com

**Keywords:** protein disulfide isomerase inhibitor, viral replication and production, antibody-dependent enhancement, dengue virus infection, human monocytic cells

## Abstract

One of several mechanisms that leads to the development of dengue hemorrhagic fever (DHF) and dengue shock syndrome (DSS) is called antibody-dependent enhancement (ADE). Monocytes can be infected by the ADE phenomenon, which occurs in dengue secondary infection. This study aimed to investigate the proteins involved in ADE of DENV infection in the human monocytic cell line U937. The phosphoproteins were used to perform and analyze for protein expression using mass spectrometry (GeLC-MS/MS). The differential phosphoproteins revealed 1131 altered proteins compared between isotype- and DENV-specific antibody-treated monocytes. The altered proteins revealed 558 upregulated proteins and 573 downregulated proteins. Protein disulfide isomerase (PDI), which is an enzyme that had a high-ranking fold change and that catalyzes the formation, breakage, and rearrangement of disulfide bonds within a protein molecule, was selected for further study. PDI was found to be important for dengue virus infectivity during the ADE model. The effect of PDI inhibition was also shown to be involved in the early stage of life cycle by time-of-drug-addition assay. These results suggest that PDI is important for protein translation and virion assembly of dengue virus during infection in human monocytes, and it may play a significant role as a chaperone to stabilize dengue protein synthesis.

## 1. Introduction

Dengue virus (DENV) infection is an anthropod-borne viral infection that is a major global public health burden, with approximately 50 new million cases reported annually worldwide [1]. DENV belongs to the family *Flaviviridae*, the genus *Flavivirus*, and is a positive single-stranded RNA virus that is transmitted to humans by *Aedes* mosquitoes [2]. Its genome size is approximately 11 kb, and it encodes for three structural proteins (capsid protein (C), membrane protein (M), and envelope protein (E)) and seven nonstructural proteins (NS1, NS2a, NS2b, NS3, NS4a, NS4b, and NS5) [3]. DENV has four antigenically different serotypes (DENV1, DENV2, DENV3, and DENV4) that were characterized from plaque reduction neutralization assay data, and the 4 DENV serotypes share 70–80% amino acid sequence similarity in whole structural and non-structural proteins [3]. Clinical manifestation of dengue infection ranges from asymptomatic cases of dengue fever (DF) to the more severe dengue hemorrhagic fever (DHF) and dengue shock syndrome (DSS) [4]. The pathogenesis of complicated DENV infection is not clearly understood, but viral factors and host immune factors may influence disease severity.

The question why some patients develop DF (mild disease) and others develop DHF or DSS (severe disease) continues to be investigated and debated. It was previously reported that secondary infections led to more severe dengue disease [5]. Epidemiological research showed pre-existing humoral immunity against DENV to be a predisposing factor for the severe form of the disease [5,6]. Heterotypic antibodies with subneutralizing properties from different serotypes of DENV or waning concentrations of homotypic antibodies were found to enhance DENV infectivity in vitro and in vivo. This mechanism of host immunity is called antibody-dependent enhancement (ADE) [7,8]. ADE of dengue virus (DENV) infection is an important process of secondary infection that results in the pathogenesis of severe dengue (SD) in humans [9]. Enhancement is mediated via interaction between the virus-antibody complex and Fcγ receptors [10]. It has been proposed that subneutralizing antibody concentrations of previous infection facilitate viral infection of Fc receptor-bearing cells, which stimulates virus replication and production [5,11]. Human cell line U937 consists of human monocytic cells that present Fc receptors that can be used as a model of in vitro ADE conditions [5,11].

Using this model and depending on the infection history of an individual, two distinct infection mechanisms can be distinguished from each other: infection in the absence or presence of DENV antibodies, or ADE condition. In infection without ADE condition, cell binding is mediated by the DENV protein [4], and can occur via a wide range of attachment factors; however, during DENV ADE, cell binding occurs via the Fcγ receptor (FcγR). The current hypothesis is that DENV particles employ ADE-specific pathways to enter and infect cells, which leads to a higher number of infected cells, altered immune responses, and increased virus infectivity [2]. DENV infection via the Fc receptor-mediated pathway is associated with a signal transduction that is able to suppress the transcription of antiviral response [12,13]. Thus, the signal transduction of the ADE-specific pathway is important for the pathogenesis of dengue virus infection in humans. Signaling molecules and pathways have recently been described in ADE condition of DENV and Ebola virus. DENV-ADE infection in monocytes could induce early production of ISG (NOS2) via the RLR-MAVS signaling axis independent of the IFNs pathway [14]. ADE-DENV infection also induces an increase in IL-10 in monocytes [14,15,16], whereas a reduction in IL-10 is observed in macrophages [15]. Moreover, in vitro ADE of Ebola infection in FcγRIIa-expressed Jurkat T cells required FcγRIIa to activate the Src signaling pathway, which led to increased viral entry into the cells [17]. Signal transduction occurs when an extracellular signaling molecule activates a specific receptor located on the cell surface or inside the cell, such as ADE [18,19]. The activated receptor then triggers a biochemical chain of events inside the cell that instigates a response. Signal transduction controls and regulates many biological processes of function, including cell proliferation, cell death, and cell division [19]. Abnormality in the signaling pathway may lead to serious complication and disease.

The aim of this study was to investigate the role of signal transduction in human monocytes (U937) during dengue virus infection in the presence of immune serum or under ADE condition. Phosphoprotein enrichment was performed to enrich the proteins involved in signal transduction, after which the differential phosphoproteins were analyzed by one-dimensional gel electrophoresis, and the proteins were analyzed by mass spectrometry. Finally, proteins of interest were evaluated to observe whether pharmacologic agents or biomolecules affect protein function.

## 2. Materials and Methods

### 2.1. Dengue Viruses and Antibodies

DENV1 strain Hawaii, DENV2 strain 16681, DENV3 strain H87, DENV4 strain H241, and mouse monoclonal antibody against DENV 1–4 (4G2) were generously provided by the Armed Forces Research Institute of Medical Sciences (AFRIMS), Bangkok, Thailand.

### 2.2. Dengue Virus Propagation

DENV serotypes (DENV1 strain Hawaii, DENV2 strain 16681, DENV3 strain H87, and DENV4 strain H241) were propagated at 28 °C in C6/36 cell line. Approximately 8 × 10^6^ cells were grown in a 75 cm^2^ tissue culture flask (Costar, Cambridge, MA, USA) in L-15 medium supplemented with 10% FBS and incubated at 28 °C for 1 day. The cell monolayer was infected with DENV at a multiplicity of infection (MOI) of 0.01 in L-15 medium supplemented with 2% FBS for a total volume of 5 mL at room temperature (RT) for 3 h with slow rotating. The culture supernatant was then replaced with 15 mL of L-15 medium supplemented with 2% FBS and further incubated at 28 °C for 6 days. Supernatant of infected cells was collected by centrifugation at 400× *g* for 5 min at 4 °C to remove cell debris. Supernatants were aliquoted and maintained at −70 °C until use.

### 2.3. Dengue Virus Titration

Focus-forming unit assay (FFU) was performed for virus titration. Vero cells were cultivated in 96-well plates at 3 × 10^4^ cells per well for 1 day or until confluence. Monolayer cells were infected with a serial 10-fold dilution of viral supernatants for 2 h in a 37 °C, 5% CO_2_ incubator. Virus supernatant was added with 100 μL of overlay medium (2× MEM medium supplemented with 3% FBS and 1.5% carboxy methyl cellulose) for 3 days in a 37 °C, 5% CO_2_ incubator. Infected cells were then stained with antibody against viral protein and the viral titer was counted by FFU assay. Briefly, infected cells were washed and fixed with 3.8% formaldehyde. Cells were then permeabilized with 1% Triton X-100 and stained with a 4G2 antibody (anti-E DI/II). Finally, secondary antibody labeled with horse radish peroxidase (HRP) was added to react with mouse Igs, followed by color detection with substrate for HRP to observe the focus-forming units of infected cells. Stained foci were counted under an inverted light microscope (Olympus CKX41; Olympus Corporation, Tokyo, Japan) at a magnification of 100×, and that value was used to calculate the viral titer, which was reported as focus-forming units (FFU)/mL.

### 2.4. Antibody-Dependent Enhancement Assay

Purified 4G2 antibody (2.86 mg/mL) was diluted to concentrations ranging from 62.5 ng/mL to 1000 ng/mL, and then incubated with virus at an MOI of 10 for 30 min in a 37 °C, 5% CO_2_ incubator to allow immune complex formation. Isotype-matched Igs was used as a control. The immune complex solution of each tube was added into a tube containing 6.5 × 10^6^ U937 cells and incubated for an additional 2 h. The solution mixtures were centrifuged and the cell pellets were resuspended in RPMI 1640 medium containing 2% FBS, and incubated in a 37 °C, 5% CO_2_ incubator for 24 h. Infected cells were collected and used for the study experiments. Once the optimal concentration of 4G2 was identified, it was used throughout the study.

### 2.5. Detection of Cell Viability

Trypan blue exclusion assay was performed to calculate of percentage of cell viability. Mock and treated samples were collected for cell viability analyses. Ten microliters of cell suspension were mixed with equal volume of 0.4% trypan blue (Sigma-Aldrich, St. Louis, MO, USA) and incubated at RT for 5 min. Ten microliters of stained cells were placed in a hemocytometer and observed under an Olympus CKX41 light microscope at a magnification of 40×. Viable (unstained) and dead (stained) cells were counted. The percentage of viable cells was the number of viable cells divided by the number of dead cells plus the number of viable cells.

### 2.6. Phosphoprotein Enrichment

Phosphoproteins were purified using a TALON PMAC Phosphoprotein Enrichment Kit (Clontech, Mountain View, CA, USA). Briefly, cells were collected by centrifugation at 500× *g* for 5 min, washed with PBS 3 times, and aspirated to remove any residual traces of liquid. The cell pellet was frozen in liquid nitrogen and then resuspended in Buffer A (extraction/loading buffer) containing phosphatase inhibitors (10 mM sodium fluoride, 1 mM sodium orthovanadate) to lyse the cells. Cells were incubated at 4 °C for 10 min, after which the cell lysate was transferred to a microcentrifuge tube. Lysates were desalted by spin down at 1000× *g* for 2 min in a PD-10 desalting column (GE Healthcare, Little Chalfont, UK). Desalted samples were added into the phosphoprotein enrichment column and incubated at 4 °C for 20 min on a platform shaker. The column was washed by adding 5 mL of Buffer A. Four mL of Buffer B (elution buffer) was added and the eluate fraction was collected. The proteins present in enrichment samples were precipitated using a ProteoExtract^®^ Protein Precipitation Kit (EMD Biosciences, Darmstadt, Germany), and the proteins were centrifuged at 10,000× *g* for 10 min at 4 °C. The pellet was dried for 5 min at RT and resuspended with SDS sample buffer (0.5% (*w*/*v*) SDS in 375 mM Tris-HCl, pH 8.8). The concentration of protein fraction was determined using the Bradford method.

### 2.7. One-Dimensional Polyacrylamide Gel Electrophoresis (1D PAGE)

Thirty micrograms of phosphoproteins were mixed with SDS sample buffer (100 mM Tris-Cl (pH 6.8), 4% (*w*/*v*) SDS, 20% (*v*/*v*) glycerol, 200 mM β-mercaptoethanol, and bromophenol blue). The first-dimensional separation was performed using gel electrophoresis. Separated proteins were observed with Coomassie Brilliant Blue staining.

### 2.8. In-Gel Tryptic Digestion of 1D-PAGE with Reduction and Alkylation

The protein bands on the 1D-PAGE were excised with a clean scalpel into 13 small pieces, and the gel pieces were transferred into 1.5 mL microcentrifuge tubes. The gel pieces were washed once with 100 µL of 50 mM ammonium bicarbonate. The solution was removed after washing and the gel pieces were destained with 500 µL of 50 mM ammonium bicarbonate and 500 µL of acetonitrile (ACN). The destaining process was performed until the gel pieces were cleared. The solution was then removed from the gel pieces. Disulfide bonds were broken by adding 100 µL of 4 mM dithiothreitol (DTT) in 50 mM ammonium bicarbonate onto the gel pieces and incubating them at 60 °C for 15 min. The solution was allowed to cool to room temperature before adding 7 µL of 250 mM alpha-Iodoacetamide (IAM) for alkylation followed by additional incubation at RT in the dark for 30 min. The solution was quenched by adding 3 µL of 4 mM DTT in 50 mM ammonium bicarbonate. That solution was then discarded and 200 µL of ACN was added to dehydrate the gel pieces before incubation at room temperature for 15 min. This process was performed once more, after which the supernatant was removed and subsequently dried at RT. Peptides were digested from gel pieces by adding 10 µL of 20 µg/200 µL trypsin in 50 mM ammonium bicarbonate, 100 µL of 50 mM ammonium bicarbonate, and 100 µL of 5% ACN in 50 mM ammonium bicarbonate, followed by incubation at 37 °C overnight. Peptide solutions were subsequently added with 200 µL of ACN and incubated at RT for 20 min. Supernatants were collected into 1.5 mL microcentrifuge tubes. Peptides were concentrated using a Savant^TM^ SpeedVac^TM^ Concentrator (Thermo Fisher Scientific, Waltham, MA, USA). Peptide pellets were resuspended in 75 µL of 0.1% formic acid. Twenty-five µL of peptide solution was loaded into tubes for mass spectrometry.

### 2.9. Mass Spectrometric Analysis

Analysis was performed on an Ultimate 3000 nano-LC system (Dionex, Surrey, UK), and the eluted peptides were directly analyzed by MicroTOF Q II mass spectrometer (Bruker, Bremen, Germany). Protein identification was performed using LC-MS/MS results that were entered into an NCBI database search using Mascot version 2.4.1 (Matrix Science; London, UK). Proteins from the MASCOT search with a minimum of 2 peptides and a minimum score of 20 were filtered.

### 2.10. Western Blot Analysis

Mock and treated samples were lysed with RIPA buffer (50 mM Tris HCL pH 8.0, 150 mM NaCl, 1% NP-40, 2 mM EDTA, 0.1% SDS). Fifty µg of protein was resolved on 10% SDS-PAGE and transferred onto nitrocellulose membrane using a semi-dry transfer apparatus (Bio-Rad, Hercules, CA, USA) at 85 mA for 1 h. Non-specific binding proteins were blocked using 5% skimmed milk in PBS for 1 h followed by incubation overnight with goat polyclonal anti-PDI at 1:500 dilution in 5% skim milk/PBS at 4 °C. After washing 3 times with PBS, the membranes were incubated with a horseradish peroxidase-conjugated secondary antibody for 1 h at RT. Signal visualization was then performed using a Luminata Forte Western HRP Substrate Enhanced Chemiluminescence Detection Kit (Merck Millipore, Burlington, MA, USA) and exposed to Fuji medical X-ray film (Fujifilm Corporation, Tokyo, Japan). Signal visualization of the control loading gel was performed using antibodies against GAPDH or β-actin at 1:1000 dilution.

### 2.11. Functional Analysis of Protein Disulfide Isomerase

To test the function of altered proteins, protein disulfide isomerase (PDI), which had high-ranking fold-changes of upregulated level, was chosen for further analysis. Chemical inhibitors, including PDI inhibitor; bacitracin (Sigma-Aldrich), which was dissolved in water at a concentration of 50 μg/μL; or, goat polyclonal anti-PDI antibody (ab110195; Abcam, Cambridge, UK) were used to treat cells during ADE assay for 24 h or 48 h post infection. Cell lysates and culture supernatants were used to detect intracellular virus and extracellular virus titer, respectively, by FFU assay.

### 2.12. Time-of-Drug-Addition Assay

To determine the effect of bacitracin on the stages of the virus life cycle, U937 cells were cultivated in 24-well plates at 1 × 10^5^ cells per well. Cells were infected with DENV2 strain 16681 using ADE assay for 1 h on ice. The plate was centrifuged for 5 min at 400× *g* to discard the supernatant medium and washed with PBS three times. Cells were then treated with 3 mM of bacitracin diluted in RPMI 1640 complete medium at 0, 2, 4, 6, 8, 12, 18, or 24 h post infection. The supernatants were harvested at 24 h postinfection and measured for viral titer by FFU assay.

### 2.13. Immunofluorescence Staining

The localization of the host was determined by immunofluorescence staining (IFA). For surface staining, U937 cells (2 × 10^5^ cells) were immunostained with primary antibodies in 1% BSA in PBS using anti-FcγRIIa (15625-1-AP; Proteintech Group, Chicago, IL, USA) at 1:50 dilution at RT for 1 h, and then washed with PBS three times. PDI antibody Alexa Fluor^®^ 488-conjugated (sc-74551 AF488; Santa Cruz Biotechnology, Dallas, TX, USA) at 1:25 dilution, secondary antibodies of donkey anti-rabbit Alexa Fluor^®^ 555-conjugated at 1:500 dilution, and the nucleus were stained with Hoechst dye in 2% FBS in PBS at RT in the dark for 1 h. After being washed three times with PBS, cells were fixed with 4% paraformaldehyde in PBS for 20 min and then washed with PBS. Cells were mounted with 50% glycerol in PBS on a glass coverslip and then sealed with another coverslip. That coverslip assembly was then mounted onto a microscope slide with nail polish. Immunofluorescence imaging was visualized under laser confocal microscope (LSM 800 with Airyscan; Carl Zeiss, Jena, Germany).

### 2.14. Data Analysis

Comparisons of data were analyzed by unpaired *t*-test using GraphPad Prism version 5.01 (GraphPad Software, Inc., San Diego, CA, USA). Statistical significance was indicated as *p* < 0.05.

## 3. Results

### 3.1. Enhancement of DENV2 Infection with Cross-Reactive Anti-E Antibody in Human Monocytic Cell Lines

**Figure 1:**
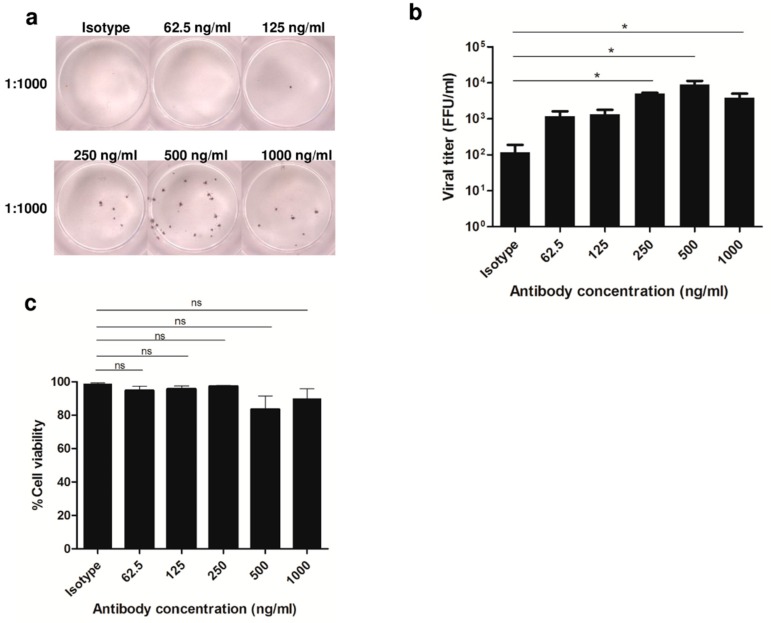
Optimization of ADE condition with DENV2 infection. The enhancement of virus infection of 4G2 mAb and isotype control (IgG2a Ab) was assessed with DENV2 strain 16681 at an MOI of 10 in U937 cells for 24 h. Viral titer and cell viability was determined by focus-forming unit (FFU) assay and trypan blue staining, respectively (**b**,**c**). A photograph of representative FFU assay is shown in (**a**). A *p*-value < 0.05 shown as * indicates statistical significance, (ns, non-significant).

We determined the condition of in vitro ADE phenomenon with DENV2 in myeloid cell line. U937 cells were evaluated by ADE assay. About 1 μg/mL of 4G2 Ab concentration was serially 2-fold diluted for use in enhancement experiments of DENV2 infection at an MOI of 10 in U937 cells. Percentage of infection and cell viability was measured by FFU and trypan blue staining, respectively. The results showed enhancement of infection to be dose-dependent (Figure 1a). DENV2 infection in the presence of 4G2 Ab at a concentration of 250 ng/mL showed a high level of infection (Figure 1b), and a low percentage of cell death (Figure 1c). We therefore determined the 250 ng/mL concentration of 4G2 antibody with DENV2 at an MOI of 10 to be suitable for cell survival and infection in ADE condition.

### 3.2. Altered Phosphoprotein Response to DENV2 Infection in Human Monocytic Cells under ADE Condition

**Figure 2:**
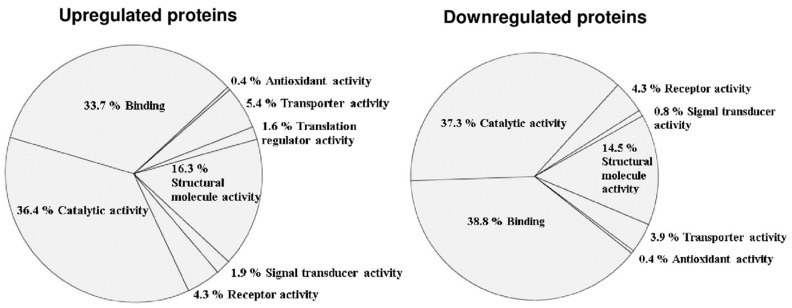
Classification of altered proteins based on molecular function. The percentage of altered proteins in DENV2-infected cells under ADE infection compared to isotype control is demonstrated by pie chart. Upregulated proteins and downregulated proteins were provided by PANTHER version 11.1 (Protein ANalysisTHrough Evolutionary Relationships). All results were analyzed from three independent experiments.

Further experiments were performed, and proteomic analysis was used to screen proteins that play a role in pathogenesis during ADE of DENV infection. After 24 h of DENV2 infection with ADE condition or ADE control (isotype Igs), infected cell lysates were enriched with phosphoproteins using a TALON PMAC Phosphoprotein Enrichment Kit (Clontech Laboratories, Inc. Mountain View, CA, USA) before being subjected to GeLC-MS/MS. For GeLC-MS/MS, all enriched phosphoprotein samples were determined using 1D SDS-PAGE and stained with Coomassie Brilliant Blue. The proteins were then excised and cut into small pieces for tryptic digestion, after which the peptides were subjected to mass spectrometry (LC-MS/MS). All LC-MS/MS data were searched using the Mascot search engine against the NCBI database. That search identified a difference of 1131 altered proteins between control and treated monocytes. Of those altered proteins, 90% were phosphoproteins identified from phosphopeptide detection. Five hundred and fifty-eight proteins were upregulated, and 573 were downregulated (Supplementary Table S1). A summary of high-ranking fold-changes of upregulated and downregulated altered proteins is shown in Supplementary Tables S2 and S3, respectively. These altered proteins were further classified relative to their molecular function using PANTHER software (Figure 2). The upregulated proteins consisted of 36.4% catalytic activity, 33.7% binding, 16.3% structural molecule activity, 5.4% transporter activity, 4.3% receptor activity, 1.9% signal transducer activity, 1.6% translation regulator activity, and 4.9% antioxidant activity. The downregulated proteins consisted of 37.3% catalytic activity, 38.8% binding, 14.5% structural molecule activity, 4.3% receptor activity, 3.9% transporter activity, 0.8% signal transducer activity, and 0.4% antioxidant activity. Thus, the major molecular functions of altered proteins were catalytic activity, structural molecule activity, and binding (Figure 2).

### 3.3. Validation of Protein Expression by 1D Western Blot Analysis

**Figure 3:**
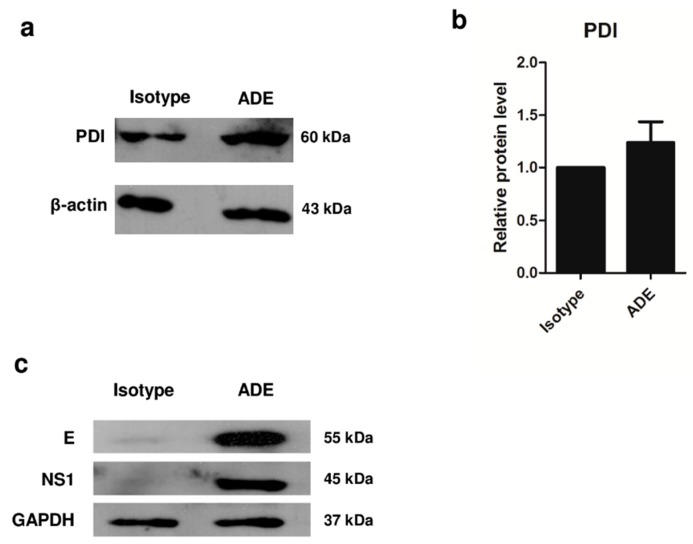
Western blot analysis of PDI protein. Whole cell extract of 24 h post-infection DENV2-infected cells under ADE infection and isotype control were resolved by 1D-PAGE and Western blot analysis. The intensity of PDI was stained with specific antibody, and the intensity of PDI was calculated and normalized with β-actin as a loading control (**a**,**b**). The infection of ADE condition was determined by Western blot analysis of DENV2 E protein (envelope protein) and NS1 protein, with GAPDH used as a control (**c**). The results of 3 independent experiments are shown as mean ± SEM.

To confirm the proteomic data, an altered protein from the data shown in Tables S1 and S2 was identified. Based on a high fold-change rank and the relevance of its function, protein disulfide isomerase (PDI) was selected for further validation of its protein expression by Western blotting. Analysis of protein intensity of PDI using ImageJ software [20] revealed higher protein level in ADE condition compared to isotype control (Figure 3). This result revealed PDI to be upregulated during ADE condition of DENV infection. The viral proteins E and NS1 were also found to be prominent in ADE condition of DENV infection. GAPDH was used as an internal control, because there was no difference in expression when compared between isotype and ADE infection (Figure 3c).

### 3.4. Role of PDI in DENV Infection, and Viral Protein Production during ADE Infection in U937 Cells

**Figure 4:**
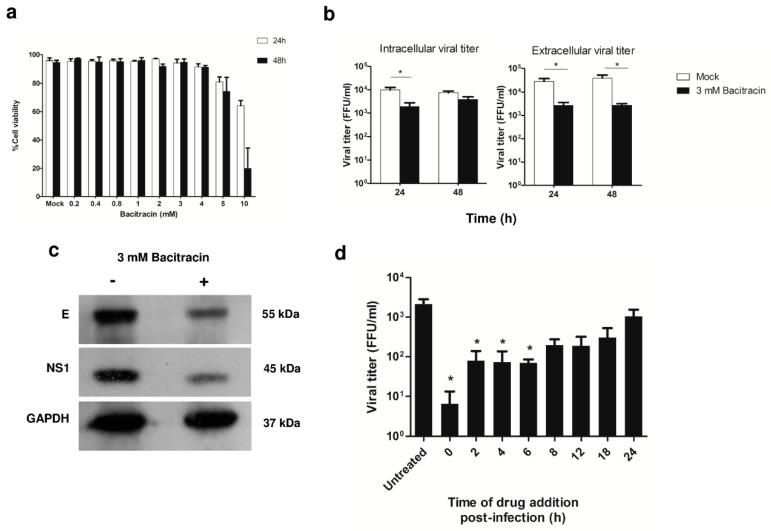
Effect of bacitracin, a PDI inhibitor, on ADE condition of DENV2 infection of U937 cells. The effect of bacitracin on cell viability is shown in (**a**). Three mM of bacitracin was found to be suitable for use in further drug treatment experiments, because it showed low effect on cell viability. The effect of bacitracin on viral titer in both intracellular (lysed cells) and extracellular viral particles (culture supernatants), as determined by FFU assay (**b**); and, viral protein production (DENV2 E and NS1), as determined by Western blot analysis (**c**). The effect of bacitracin on time-of-drug-addition in ADE condition of DENV2 infection in U937 cells was evaluated by viral titer as determined by FFU assay. Bacitracin was added at 0, 2, 4, 6, 8, 12, 18, and 24 h after ADE infection in U937 cells. At 24 h post-infection, culture supernatants (extracellular virus) were collected to determine viral titer by FFU assay (**d**). The effect of drug-addition showed dramatically decreased viral titer during 0–6 h. The results of 3 independent experiments are shown as mean ± SEM (* *p* < 0.05).

To determine the functional role of PDI in viral infection, bacitracin inhibition of PDI protein was used to determine whether PDI has an effect on DENV2 infection under ADE condition. Three mM of bacitracin was the dose that caused the least amount of cell death in mock control cells, so this concentration was considered to be optimum for further experiments (Figure 4a). ADE-infected U937 cells were treated with bacitracin. The viral titer of lysed cells (intracellular virus) and culture supernatants (extracellular virus) was examined by FFU assay. At 24 h post-infection, the viral titer was significantly decreased in both intracellular and extracellular virus particles in 3 mM bacitracin-treated cells (Figure 4b) when compared to untreated cells. Then, we evaluated whether inhibition of PDI by bacitracin upon DENV-ADE infection affected the viral protein levels of the envelope (E) and non-structural (NS) 1 proteins using Western blot analysis. At 24 h post-infection, the E and NS1 proteins were both significantly decreased in bacitracin-treated cells (Figure 4c).

### 3.5. Effect of PDI Inhibition on Virus Life Cycle Stages

Since our results showed that PDI might play a role in viral production in both intracellular and extracellular viral particles of DENV-ADE-infected cells, we set forth to investigate the effect of PDI inhibition on different stages of the virus life cycle under DENV-ADE infection using time-of-drug-addition assay. DENV-ADE infection of U937 cells was treated with 3 mM of bacitracin at 0, 2, 4, 6, 8, 12, 18, and 24 h post-infection. At 24 h post-infection, culture supernatants were collected and subjected to viral titration by FFU assay. The addition of bacitracin at 0 to 6 h post infection significantly decreased the viral titer (Figure 4d). This result suggests that bacitracin-induced inhibition of PDI occurred during the early stage of DENV-ADE infection.

### 3.6. Significance of PDI in DENV Infection during ADE Infection

**Figure 5:**
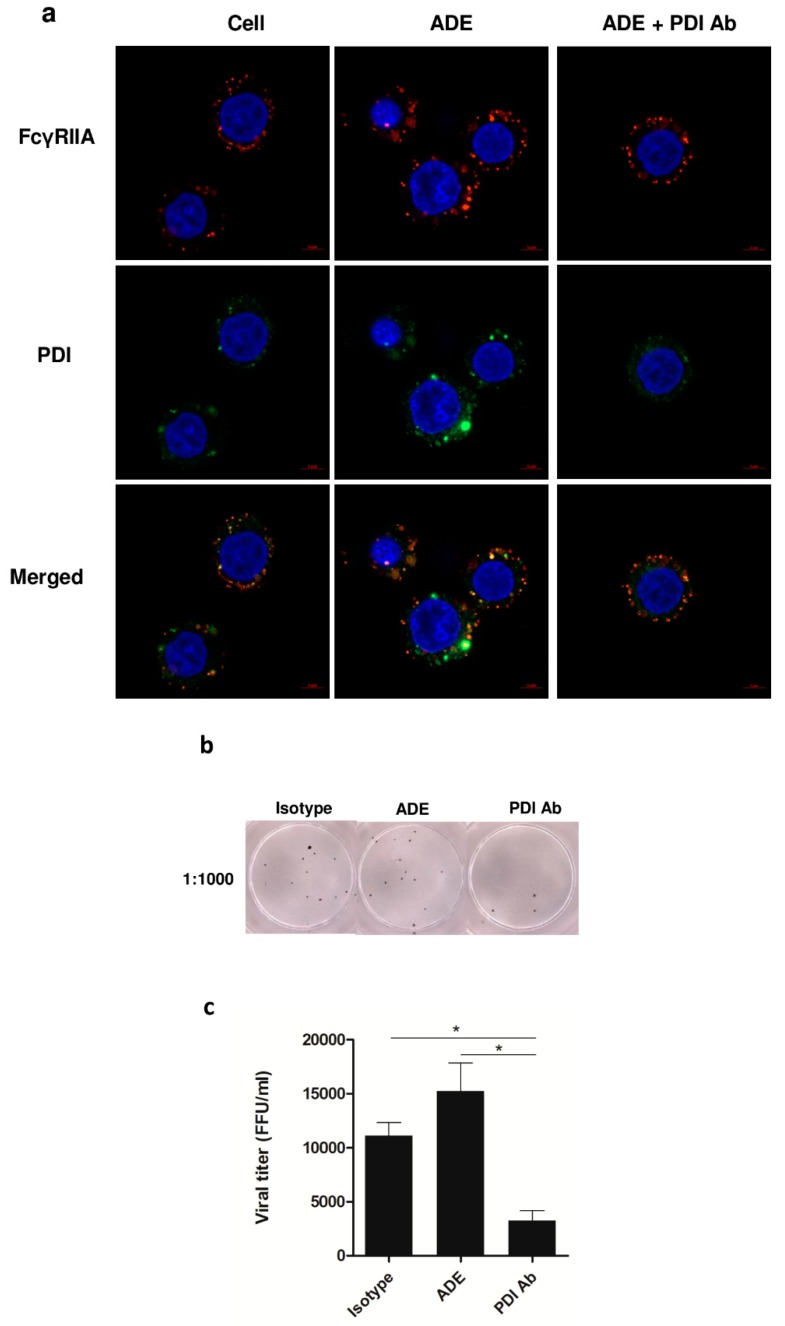
Immunofluorescence staining of FcγRIIA and PDI in U937 cells infected with ADE condition of DENV infection in the absence or presence of blocking PDI with goat polyclonal antibody. The localization of FcγRIIA (red) is shown on the surface of cells, while PDI (green) was observed both inside and on the surface of cells. Nuclei are shown in blue color (**a**) The viral titer at 24 h post-infection is shown as a photograph of the representative FFU assay (**b**), and as a histogram (**c**). The results of 3 independent experiments are shown as mean ± SEM (* *p* < 0.05).

Immunofluorescence staining showed localization of FcγRIIA on the surface of U937 cells, whereas PDI was observed both inside and on the surface of the cells in ADE condition. The expression of both proteins was decreased in the cells containing the antibody against PDI (Figure 5a). Decreased viral titer was observed in ADE infection in the antibody blocking condition with anti-PDI (Figure 5b,c).

### 3.7. Effect of Bacitracin on Viral Titers of DENV1, DENV3, and DENV4 Infection of U937 Cells

**Figure 6:**
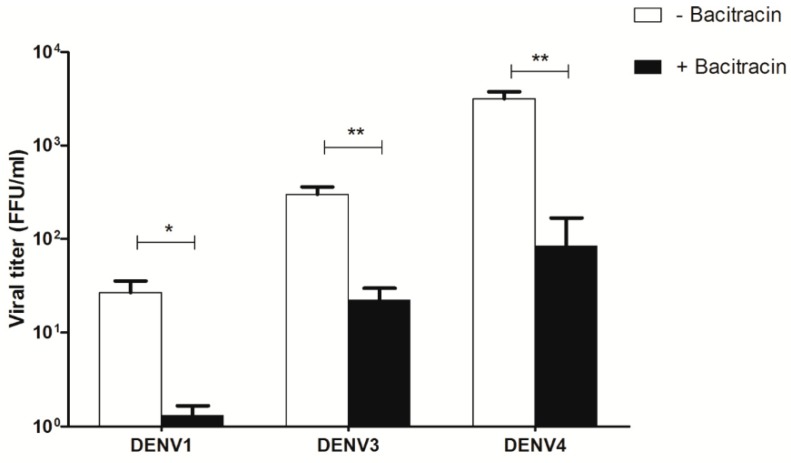
Effect of bacitracin on viral titer of ADE condition of DENV1, DENV3, and DENV4 infection in U937 cells. The viral titer of DENV1, DENV3, and DENV4 was decreased after treatment with 3 mM bacitracin. The results of 3 independent experiments are shown as mean ± SEM (* *p* < 0.05, ** *p* < 0.005).

To observe whether bacitracin affected other dengue serotypes, the viral titer of DENV1, DENV3, and DENV4 infection in U937 cells under ADE condition was determined by FFU assay. Three mM of bacitracin was used to treat U937 cells under the same conditions that were used in the DENV2 experiment. Bacitracin had the same effect on the viral titer of DENV1, DENV3, and DENV4 as it did on the viral titer of DENV2 (Figure 6). The viral titer of DENV1, DENV3, and DENV4 showed significantly decreased viral titer when compared with the untreated group.

## 4. Discussion

Monocytes represent the first line of human immune defense. Monocytes mediate dissemination of DENV after a mosquito bite, and they are one of the main targets of viral replication [21,22]. Monocytes can be infected by ADE phenomenon, which is a mechanism that contributes to the development of the severe dengue hemorrhagic fever (DHF) and dengue shock syndrome (DSS) forms of dengue disease [7,8,23]. The 4G2 monoclonal antibody against the DENV envelope protein was suitable for studying ADE condition in human monocytic cell lines because it showed enhancement activity in a dose-dependent fashion [24].

The analysis of GeLC-MS/MS data from isolated phosphoproteins by TALON PMAC Phosphoprotein Enrichment Kit also confirmed high amounts of the identified phosphoproteins. The 3 major altered proteins identified from PANTHER analysis revealed proteins involved in structural molecule activity, catalytic activity, and binding. Structural molecule activity is the action of a molecule that leads to the structural integrity of a complex, or its assembly within or outside a cell. The cytoskeleton is a structural constituent of structural molecule activity, it forms a network that is controlled by several accessory proteins, and it contributes to the cell’s shape and internal organization. Cytoskeletal elements are a group of host proteins that contribute to DENV infection, such as viral entry and release of DENV2 by actin filament interaction with DENV E protein [25,26]. Vimentin and microtubules interact directly with viral protein upon virus replication [27,28]. Our proteomic data revealed the proteins with high level in structural molecule activity to be mostly β-actin, tubulin β-4A, radixin, myosin-14, and moesin.

Catalytic activity is defined as biochemical reaction at physiological temperatures. Catalyzed reactions are known as enzymes that play a role in biologically catalyzed reactions by identifying and securing specific binding sites for substrates. In DENV infection, the viral polyproteins are processed by host proteases at sites in the endoplasmic reticulum. Our proteomic data showed proteins with high level in catalytic activity to be mostly protein disulfide-isomerase A4 (PDI), DNA-directed RNA polymerase II subunit RPB1, and glucose-6-phosphate isomerase (neuroleukin).

Binding is the selective, often stoichiometric, or non-covalent interaction of a molecule with one or more linked sites on another molecule. The binding of protein function acts as a linker to connect proteins. It can be used to capture macromolecular interactions, such as nucleic acid binding and protein binding. Binding contributes to DENV infection in many stages, including binding of virus and target receptor, which contributes to the internalization of the virus, and binding of viral proteins and ER-resident chaperones, which facilitates the folding and assembly of viral proteins. Our proteomic data revealed proteins with high level in binding to mostly be retinoic acid receptor alpha and alpha-actinin-4.

It is important to understand whether interruption of these protein functions affects pathogenesis, as well as virion and viral protein production. Of these altered proteins, the upregulated protein disulfide-isomerase (PDI) was selected for functional study based on its high rank relative to fold-change value (Table S1), and its relevant roles in the pathogenesis of DENV infection. PDI is most abundant at locations in the endoplasmic reticulum (ER), and it acts as a chaperone. PDI is formed from 4 tandem domains that contain thioredoxin-like folds. However, PDI is also detected in the nuclear envelope, cytosol, and other endomembranes, such as the Golgi, secretory vesicles, and plasma membrane. In addition, PDI on the cell surface has reducing activity for exterior thiols [29].

PDI also regulates the internalization of several viruses. HIV-1 infection increased after overexpression of PDI by increasing the fusion of viral membranes, which caused entry of the virus [30,31]. Similarly, PDI on the cell surface facilitates infection of mouse polyoma virus [32] and Newcastle disease virus [33]. In DENV infection, PDI was found to colocalize with β1 and β3 integrins on the cell surface after DENV infection, which facilitated integrin activation that then allowed entry of DENV into endothelial cells to promote viral infection of cells [34,35].

The viral entry enhancement effect of PDI was recently found to be associated with lipid raft in human monocytic cell line THP-1 [36]. In our study, PDI was analyzed by PANTHER software. That analysis revealed catalytic activity in PANTHER protein class, and cellular process and response to stimulus in PANTHER biological process. Increased PDI in cell lysates as determined by Western blot analysis was well correlated with proteomic data. Inhibition of PDI also inhibited DENV2 production during DENV-ADE infection in U937 cells. Although we could not exclude the possible induction of PDI expression caused by FcγR on monocytes during ADE condition, the isotype IgG used as a control did not show any significant increase in the level of PDI, which is what was observed in ADE-DENV-infected monocytes.

A commonly used inhibitor of PDI is bacitracin, which is a mixture of cyclic polypeptides. Bacitracin can inhibit the reductive activity of PDI, and it has been used to study the effect of PDI in the murine macrophage-like cell line RAW 264.7 [37,38]. Significant PDI inhibition of TNF-α gene expression and production was reported. The downregulation of PDI expression could inhibit proinflammatory cytokine production in chemical-induced sepsis in rats [39]. A PDI knockdown experiment with siRNA resulted in reduced infection of mouse polyomavirus, but no change in SV40 infection in HeLa cells was observed. This result was explained by the role of PDI in reducing and rearranging disulfide bonds on stabilizing the capsomeres of mouse polyomavirus [32]. This virus appeared to reach the ER normally, but failed to exit the ER, which caused a defect in virus disassembly in the ER.

PDI inhibition effectuated by bacitracin administration decreased virus production in both intracellular and extracellular virus particles, as well as in the expressions of the virus proteins E and NS1. Bacitracin has demonstrated effectiveness in all four DENV serotypes. Our data is similar to the result from an experiment performed in another ER chaperone protein (glucose regulated protein 78 (GRP78)). GRP78 is an ER-associated member of the HSP70 family of chaperone proteins that was found in higher levels in DENV-infected K562 erythroleukemic cell line [40]. The suppression of GRP78 with SubAB, a toxin derived from Shiga toxigenic *E. coli* strains, in 293 cells revealed a dramatic decrease in DENV antigen production, and an increase rather than a reduction in viral RNA level and/or virus replication. Moreover, PDI can associate with ubiquitin and play a role in controlling the degradation of key molecules via the ubiquitin-proteasome pathway [41].

The results of our study suggest that PDI might be required for DENV-ADE infection in human monocytic cell lines. Bacitracin-induced inhibition of DENV was shown to be group-specific, because it showed suppression of viral replication in all 4 serotypes of DENV. It is also important to understand which stage or stages of infection is/are affected by bacitracin within cells. To more closely identify the scope of the effect of PDI on DENV-ADE infection, time-of-drug-addition assay was performed. The key time points of the viral life cycle are described, as follows: within 2 h post-infection, the virus attaches and enters into the cell; at 5 h post-infection, viral RNA is translated and modified to mature protein; at 5 to 12 h post-infection, viral RNA is replicated and amplified genome; and, after 12 h, viral RNA and viral protein are assembled into virus particles and secreted from the host cells [42,43,44]. The evidence indicates that bacitracin should interrupt or inhibit any stage of virus infection, including cell binding, entry, translation, RNA synthesis, and assembly. The present study showed that the addition of bacitracin at 0 to 6 h post-infection significantly decreased the viral titer. This finding suggests that bacitracin inhibited the early stage of DENV-ADE infection. In addition, the viral titer was lowest when bacitracin was added at 0 h post-infection, which suggests that binding and/or internalization might be interrupted by bacitracin. However, after the addition of bacitracin at 2 h to 6 h post-infection, the viral titer, RNA synthesis, and translation were all decreased. We could not exclude the possible direct effect of bacitracin against the virus, but the results of time-of-drug-addition should support that the effect of bacitracin did not occur in the late stage of infection or after the virus entered into the cells. The experiment where we blocked PDI with antibody also supports the effect that PDI inhibitor has on virus infection (Figure 4d and Figure 5). Moreover, the colocalization of PDI and lipid rafts on the cell surface mediates DENV entry, and the inhibition of lipid rafts decreased cell surface PDI along with DENV cell surface binding [34,36]. Lipid rafts were required for DENV-ADE infection in U937 cells [10]. Fc-FcγR interactions are associated with lipid rafts for efficient signaling [45,46]. The results of this study suggest that PDI is an important protein for viral replication, and inhibition of this protein could minimize both the severity of dengue virus infection and virus replication in human monocytic cells. Taken together, the findings of this study suggest PDI inhibition as a new therapeutic approach to treating dengue virus infection. 

## 5. Conclusions

Monocytes were reported to be the primary target in ADE condition of DENV infection. The present study investigated altered proteins involved in signal transduction during DENV-ADE in U937 cells by GeLC-MS/MS. Relative to type of molecular function, most of the altered proteins were involved in catalytic activity, structural molecule activity, or binding activity. Protein disulfide-isomerase (PDI) was found to be upregulated and involved in viral protein and virion production. Inhibition of PDI with bacitracin caused dramatically reduced viral replication, and this bacitracin-induced inhibition of PDI was observed in the early stages of the life cycle of DENV in DENV-ADE infection. These results provide a better understanding of the mechanism of ADE in DENV infection.

## Supplementary Materials

The following are available online at https://www.mdpi.com/1999-4915/11/2/155/s1, Table S1: Altered phosphoproteins in antibody-dependent enhancement (ADE) of DENV2-infected U937 cells, Table S2: Summary of high-ranking fold-changes in upregulated altered proteins during ADE of DENV2-infected U937 cells, and, Table S3: Summary of high-ranking fold-changes in downregulated altered proteins during ADE of DENV2-infected U937 cells.
