# Supplementary material for: Protein Disulfide Isomerase Inhibitor Suppresses Viral Replication and Production during Antibody-Dependent Enhancement of Dengue Virus Infection in Human Monocytic Cells
Source: Viruses. 2019 Feb 13;11(2):155. doi: 10.3390/v11020155 (PMC6410196; doi:10.3390/v11020155)
Supplement: Supplementary file 1 [file viruses-11-00155-s001.zip › Supplementary Table 1 Nov 2018.docx]

**Supplementary Table 1.** Altered phosphoproteins in antibody-depedent enhancement (ADE) of DENV2-infected U937 cells

| **No.** | **NCBI ID** | **Protein** | **pI** | **MW (Da)** | **Identification score** | **No. of matched peptides** | **%cov** | **Mock (Mean±SD)** | **Treated (Mean±SD)** | **Ratio (Treated/Mock)** |
| --- | --- | --- | --- | --- | --- | --- | --- | --- | --- | --- |
| 1 | gi\|119389581 | Chain C, Ctd-Specific Phosphatase Scp1 In Complex With Peptide From C-Terminal Domain Of Rna Polymerase Ii | 5.25 | 848 | 94 | 4 | 100 | no emPAI | 5.59±0 | n/a |
| 2 | gi\|190238 | nucleolar phosphoprotein B23, partial | 9.72 | 9189 | 157 | 5 | 52.4 | no emPAI | 3.98±0 | n/a |
| 3 | gi\|190240 | nucleolar phosphoprotein B23, partial | 9.84 | 9292 | 144 | 5 | 56.1 | 0±0 | 3.89±0 | n/a |
| 4 | gi\|119395750 | keratin, type II cytoskeletal 1 | 8.15 | 65999 | 872 | 7 | 35.2 | 0±0 | 0.88±0 | n/a |
| 5 | gi\|578802476 | PREDICTED: protein disulfide-isomerase A6 isoform X4 | 5.79 | 53510 | 741 | 7 | 31 | no emPAI | 0.87±0.27 | n/a |
| 6 | gi\|48257068 | HSPA8 protein, partial | 5.36 | 64633 | 791 | 7 | 31.2 | 0±0 | 0.81±0.62 | n/a |
| 7 | gi\|178024 | beta-actin, partial | 6.75 | 7794 | 133 | 4 | 52.1 | 0±0 | 0.78±0.33 | n/a |
| 8 | gi\|8569617 | Chain C, Crystal Structure Of The Moesin Ferm DomainTAIL DOMAIN COMPLEX | 7 | 10341 | 101 | 4 | 33.3 | no emPAI | 0.78±0 | n/a |
| 9 | gi\|574584803 | tubulin beta-4A chain isoform 1 | 4.92 | 54432 | 434 | 6 | 28.1 | 0±0 | 0.7±0.1 | n/a |
| 10 | gi\|6063147 | ezrin | 9.19 | 19224 | 336 | 5 | 42.1 | no emPAI | 0.65±0.27 | n/a |
| 11 | gi\|12620919 | prosomal P27K protein | 9.15 | 6196 | 125 | 5 | 62.1 | 0±0 | 0.58±0 | n/a |
| 12 | gi\|178027 | alpha-actin | 5.23 | 42081 | 445 | 6 | 27.9 | 0±0 | 0.55±0.21 | n/a |
| 13 | gi\|30908859 | actin alpha 1 skeletal muscle protein | 5.71 | 28133 | 335 | 5 | 27.2 | 0±0 | 0.48±0.26 | n/a |
| 14 | gi\|4139784 | Chain A, Canine Gdp-Ran Q69l Mutant | 7.01 | 24393 | 242 | 5 | 27.3 | 0±0 | 0.47±0 | n/a |
| 15 | gi\|187661962 | RecName: Full=Putative tubulin beta chain-like protein ENSP00000290377 | 4.77 | 41748 | 160 | 6 | 8.1 | 0±0 | 0.46±0 | n/a |
| 16 | gi\|346652078 | Chain A, Crystal Structure Of P97n In Complex With The C-Terminus Of Gp78 | 6.19 | 21025 | 157 | 5 | 25.1 | 0±0 | 0.46±0.11 | n/a |
| 17 | gi\|33990951 | RDX protein, partial | 9.53 | 40371 | 373 | 6 | 21.2 | 0±0 | 0.39±0.22 | n/a |
| 18 | gi\|28436809 | Radixin | 5.88 | 68522 | 370 | 6 | 13.7 | 0±0 | 0.29±0.03 | n/a |
| 19 | gi\|14326412 | short heat shock protein 60 Hsp60s2 | 4.62 | 27079 | 159 | 5 | 12.4 | 0±0 | 0.26±0 | n/a |

**Supplementary Table 1.** Altered phosphoproteins in antibody-depedent enhancement (ADE) of DENV2-infected U937 cells (cont.)

| **No.** | **NCBI ID** | **Protein** | **pI** | **MW (Da)** | **Identification score** | **No. of matched peptides** | **%cov** | **Mock ๖(Mean±SD)** | **Treated (Mean±SD)** | **Ratio (Treated/Mock)** |
| --- | --- | --- | --- | --- | --- | --- | --- | --- | --- | --- |
| 20 | gi\|605603744 | Chain A, Crystal Structure Of The Human Mortalin (grp75) Atpase Domain In The Apo Form | 6.54 | 41340 | 211 | 5 | 21 | 0±0 | 0.26±0 | n/a |
| 21 | gi\|6063145 | ezrin | 9.3 | 19076 | 244 | 5 | 40.5 | 0±0 | 0.25±0.09 | n/a |
| 22 | gi\|62088144 | stathmin 1 variant | 8.47 | 15093 | 112 | 5 | 22.4 | 0±0 | 0.23±0 | n/a |
| 23 | gi\|1314308 | nucleophosmin-retinoic acid receptor alpha fusion protein NPM-RAR long form | 5.85 | 62533 | 131 | 4 | 6.9 | no emPAI | 0.23±0 | n/a |
| 24 | gi\|38455427 | T-complex protein 1 subunit delta isoform a | 7.96 | 57888 | 307 | 5 | 22.3 | no emPAI | 0.22±0.04 | n/a |
| 25 | gi\|189238 | neuroleukin | 8.43 | 63149 | 138 | 6 | 7.9 | 0±0 | 0.2±0.09 | n/a |
| 26 | gi\|119595805 | hCG41772, isoform CRA_c | 8.9 | 18242 | 67 | 2 | 19.3 | 0±0 | 0.19±0 | n/a |
| 27 | gi\|292162 | heat shock protein 86, partial | 4.56 | 35652 | 188 | 6 | 14.7 | 0±0 | 0.19±0 | n/a |
| 28 | gi\|1857526 | beta-tubulin | 5.11 | 48346 | 131 | 4 | 9.2 | 0±0 | 0.18±0.04 | n/a |
| 29 | gi\|33357460 | Chain B, Human Pyruvate Dehydrogenase | 5.95 | 37018 | 163 | 6 | 8.2 | 0±0 | 0.16±0.05 | n/a |
| 30 | gi\|2285963 | high mobility group protein 2a | 8.67 | 22902 | 87 | 3 | 15 | 0±0 | 0.15±0 | n/a |
| 31 | gi\|131412225 | keratin, type I cytoskeletal 13 isoform a | 4.91 | 49527 | 160 | 6 | 22.7 | 0±0 | 0.14±0 | n/a |
| 32 | gi\|57997573 | hypothetical protein | 4.71 | 27159 | 133 | 4 | 31 | 0±0 | 0.12±0 | n/a |
| 33 | gi\|119577215 | actinin, alpha 4, isoform CRA_c | 5.24 | 104099 | 489 | 6 | 23.1 | 0±0 | 0.11±0.05 | n/a |
| 34 | gi\|378404908 | glyceraldehyde-3-phosphate dehydrogenase isoform 2 | 7.15 | 31528 | 117 | 5 | 20.1 | 0±0 | 0.11±0 | n/a |
| 35 | gi\|453155 | keratin 9 | 5.14 | 61950 | 244 | 5 | 26.8 | 0±0 | 0.11±0 | n/a |
| 36 | gi\|350610483 | Chain A, Truncated Human Atp-Citrate Lyase With Adp And Tartrate Bound | 6.83 | 90640 | 222 | 5 | 10 | 0±0 | 0.11±0.04 | n/a |
| 37 | gi\|3157976 | alpha actinin | 5.47 | 105159 | 431 | 6 | 19 | 0±0 | 0.11±0.03 | n/a |

**Supplementary Table 1.** Altered phosphoproteins in antibody-depedent enhancement (ADE) of DENV2-infected U937 cells (cont.)

| **No.** | **NCBI ID** | **Protein** | | **pI** | | **MW (Da)** | | **Identification score** | | **No. of matched peptides** | | **%cov** | | **Mock (Mean±SD)** | | **Treated (Mean±SD)** | | **Ratio (Treated/Mock)** | |
| --- | --- | --- | --- | --- | --- | --- | --- | --- | --- | --- | --- | --- | --- | --- | --- | --- | --- | --- | --- |
| 38 | gi\|4557719 | DNA ligase 1 isoform 1 [Homo sapiens] | 5.49 | | 101673 | | 157 | | 5 | | 6.5 | | no emPAI | | 0.1±0 | | n/a | |  |
| 39 | gi\|119616807 | hCG2043493, partial | 9.26 | | 33954 | | 111 | | 4 | | 8.9 | | 0±0 | | 0.1±0 | | n/a | |  |
| 40 | gi\|62898171 | L-plastin variant | 5.2 | | 70215 | | 120 | | 5 | | 12.4 | | 0±0 | | 0.1±0 | | n/a | |  |
| 41 | gi\|56967028 | Chain A, Crystal Structure Of Human Dna Ligase I Bound To 5~-Adenylated, Nicked Dna | 5.79 | | 76060 | | 180 | | 6 | | 11.8 | | no emPAI | | 0.09±0 | | n/a | |  |
| 42 | gi\|119574084 | guanine nucleotide binding protein (G protein), beta polypeptide 2-like 1, isoform CRA_h | 8.67 | | 39680 | | 72 | | 2 | | 5.8 | | 0±0 | | 0.08±0 | | n/a | |  |
| 43 | gi\|119581140 | keratin, hair, acidic, 5, isoform CRA_a | 4.85 | | 50339 | | 107 | | 5 | | 6.4 | | 0±0 | | 0.07±0 | | n/a | |  |
| 44 | gi\|12311759 | type I hair keratin 8 | 4.79 | | 50457 | | 71 | | 2 | | 6.1 | | 0±0 | | 0.07±0 | | n/a | |  |
| 45 | gi\|186685 | keratin type 16 | 4.97 | | 50668 | | 131 | | 4 | | 13.2 | | 0±0 | | 0.07±0 | | n/a | |  |
| 46 | gi\|31074643 | type I inner root sheath specific keratin 25 irs4 | 5.33 | | 50564 | | 96 | | 4 | | 8.4 | | 0±0 | | 0.07±0 | | n/a | |  |
| 47 | gi\|3329390 | SKD1 homolog | 6.75 | | 49255 | | 67 | | 2 | | 7.4 | | no emPAI | | 0.07±0 | | n/a | |  |
| 48 | gi\|3724107 | type I hair keratin 5 | 4.75 | | 47556 | | 73 | | 2 | | 6.6 | | 0±0 | | 0.07±0 | | n/a | |  |
| 49 | gi\|4929561 | CGI-46 protein | 6.25 | | 48261 | | 126 | | 5 | | 13.1 | | 0±0 | | 0.07±0 | | n/a | |  |
| 50 | gi\|7108915 | glucocorticoid receptor AF-1 specific elongation factor | 9.08 | | 46240 | | 124 | | 5 | | 12.4 | | 0±0 | | 0.07±0 | | n/a | |  |
| 51 | gi\|94538345 | keratin, type I cuticular Ha5 | 4.85 | | 50329 | | 80 | | 3 | | 6.4 | | 0±0 | | 0.07±0 | | n/a | |  |
| 52 | gi\|1195531 | type I keratin 16 | 4.99 | | 51206 | | 122 | | 5 | | 10.6 | | 0±0 | | 0.06±0 | | n/a | |  |
| 53 | gi\|12803709 | Keratin 14 | 5.09 | | 51619 | | 148 | | 5 | | 16.9 | | no emPAI | | 0.06±0 | | n/a | |  |
| 54 | gi\|6739602 | talin | 5.77 | | 269486 | | 264 | | 5 | | 6.3 | | 0±0 | | 0.04±0 | | n/a | |  |
| 55 | gi\|35046 | NF-M | 4.9 | | 102386 | | 67 | | 2 | | 3.9 | | 0±0 | | 0.03±0 | | n/a | |  |
| 56 | gi\|116284394 | myosin-14 isoform 2 | 5.52 | | 227732 | | 152 | | 5 | | 3.7 | | 0±0 | | 0.03±0.02 | | n/a | |  |
| 57 | gi\|7243019 | KIAA1319 protein | 5.44 | | 137518 | | 80 | | 3 | | 2.9 | | no emPAI | | 0.02±0 | | n/a | |  |
| 58 | gi\|119608589 | calmodulin regulated spectrin-associated protein 1, isoform CRA_a | 6.27 | | 160293 | | 103 | | 4 | | 2.8 | | 0±0 | | 0.02±0 | | n/a | |  |

**Supplementary Table 1.** Altered phosphoproteins in antibody-depedent enhancement (ADE) of DENV2-infected U937 cells (cont.)

| **No.** | **NCBI ID** | **Protein** | **pI** | **MW (Da)** | **Identification score** | **no. of matched peptides** | **%cov** | **Mock (Mean±SD)** | **Treated (Mean±SD)** | **Ratio (Treated/Mock)** |
| --- | --- | --- | --- | --- | --- | --- | --- | --- | --- | --- |
| 59 | gi\|90403581 | meiosis inhibitor protein 1 | 6.24 | 141071 | 110 | 4 | 4.6 | 0±0 | no emPAI | n/a |
| 60 | gi\|825671 | B23 nucleophosmin (280 AA) | 4.71 | 30919 | 247 | 5 | 24.6 | 0.4±0.07 | 1.5±0 | 3.69 |
| 61 | gi\|307086 | keratin-10 | 5.11 | 46359 | 350 | 6 | 31.6 | 0.15±0 | 0.51±0 | 3.40 |
| 62 | gi\|119581085 | keratin 10 (epidermolytic hyperkeratosis; keratosis palmaris et plantaris), isoform CRA_b | 5.13 | 63308 | 509 | 6 | 33.8 | 0.16±0 | 0.5±0 | 3.13 |
| 63 | gi\|623409 | keratin 10 | 5.01 | 57213 | 390 | 6 | 30.5 | 0.18±0 | 0.4±0 | 2.22 |
| 64 | gi\|181402 | epidermal cytokeratin 2 | 8.07 | 65825 | 417 | 6 | 25.7 | 0.1±0 | 0.22±0 | 2.20 |
| 65 | gi\|119617032 | keratin 6B, isoform CRA_a | 8.38 | 59874 | 222 | 5 | 12.4 | 0.05±0 | 0.11±0 | 2.20 |
| 66 | gi\|8394076 | proteasome subunit alpha type-6 [Rattus norvegicus] | 6.34 | 27382 | 212 | 5 | 30.1 | 0.12±0 | 0.26±0 | 2.17 |
| 67 | gi\|13124875 | myosin-11 isoform SM2A | 5.44 | 223439 | 221 | 5 | 4.3 | 0.02±0.01 | 0.04±0.02 | 2.00 |
| 68 | gi\|6005942 | transitional endoplasmic reticulum ATPase | 5.14 | 89266 | 581 | 6 | 21.1 | 0.23±0.11 | 0.46±0.13 | 2.00 |
| 69 | gi\|5453603 | T-complex protein 1 subunit beta isoform 1 | 6.01 | 57452 | 444 | 6 | 37.4 | 0.22±0.04 | 0.4±0.2 | 1.86 |
| 70 | gi\|119590106 | hCG27371 | 9.11 | 9203 | 114 | 4 | 28.6 | 0.9±0 | 1.62±0 | 1.80 |
| 71 | gi\|11935049 | keratin 1 | 8.16 | 66027 | 868 | 7 | 35.2 | 0.52±0.07 | 0.88±0 | 1.69 |
| 72 | gi\|34740335 | tubulin alpha-1B chain [Mus musculus] | 4.94 | 50120 | 487 | 6 | 29.9 | 0.47±0.33 | 0.77±0 | 1.63 |
| 73 | gi\|386854 | type II keratin subunit protein, partial | 5.31 | 52757 | 461 | 6 | 26.8 | 0.33±0.08 | 0.53±0 | 1.62 |
| 74 | gi\|401664164 | EZR-ROS1 fusion protein | 5.8 | 98886 | 380 | 6 | 9.9 | 0.12±0.05 | 0.18±0.09 | 1.54 |
| 75 | gi\|4503483 | elongation factor 2 | 6.41 | 95277 | 547 | 6 | 19.8 | 0.17±0.11 | 0.27±0.05 | 1.53 |
| 76 | gi\|375314779 | keratin 1 | 8.15 | 66026 | 820 | 7 | 35.2 | 0.52±0.07 | 0.79±0 | 1.52 |
| 77 | gi\|441674116 | PREDICTED: LOW QUALITY PROTEIN: moesin [Nomascus leucogenys] | 5.98 | 67936 | 1146 | 8 | 42.7 | 0.99±0.14 | 1.45±0 | 1.46 |

**Supplementary Table 1.** Altered phosphoproteins in antibody-depedent enhancement (ADE) of DENV2-infected U937 cells (cont.)

| **No.** | **NCBI ID** | **Protein** | **pI** | **MW (Da)** | **Identification score** | **No. of matched peptides** | **%cov** | **Mock (Mean±SD)** | **Treated (Mean±SD)** | **Ratio (Treated/Mock)** |
| --- | --- | --- | --- | --- | --- | --- | --- | --- | --- | --- |
| 78 | gi\|4503481 | elongation factor 1-gamma | 6.25 | 50087 | 374 | 6 | 25.6 | 0.35±0.15 | 0.49±0.25 | 1.40 |
| 79 | gi\|426352367 | PREDICTED: spliceosome RNA helicase DDX39B isoform 3 [Gorilla gorilla gorilla] | 8.93 | 40551 | 133 | 4 | 18.6 | 0.13±0.05 | 0.17±0.09 | 1.36 |
| 80 | gi\|74722492 | RecName: Full=Putative heat shock protein HSP 90-beta-3; AltName: Full=Heat shock protein 90-beta c; Short=Heat shock protein 90Bc | 4.71 | 68282 | 357 | 6 | 18.3 | 0.16±0.06 | 0.21±0.06 | 1.32 |
| 81 | gi\|4507677 | endoplasmin precursor | 4.76 | 92411 | 575 | 6 | 16.9 | 0.31±0.16 | 0.4±0.03 | 1.27 |
| 82 | gi\|746815999 | tubulin beta-6 chain isoform 6 | 4.75 | 34060 | 254 | 5 | 23.3 | 0.59±0 | 0.75±0 | 1.27 |
| 83 | gi\|27754056 | tubulin beta-6 chain [Mus musculus] | 4.8 | 50058 | 216 | 5 | 21.7 | 0.21±0 | 0.25±0.04 | 1.19 |
| 84 | gi\|3287489 | Hsp89-alpha-delta-N | 5.03 | 63212 | 855 | 7 | 32.8 | 0.73±0.36 | 0.84±0.42 | 1.15 |
| 85 | gi\|4758304 | protein disulfide-isomerase A4 precursor | 4.96 | 72887 | 686 | 7 | 27.1 | 0.42±0.29 | 0.47±0.27 | 1.14 |
| 86 | gi\|578827087 | PREDICTED: pyruvate kinase PKM isoform X4 | 8.18 | 65764 | 429 | 6 | 24.1 | 0.5±0.22 | 0.56±0.15 | 1.13 |
| 87 | gi\|36796 | t-complex polypeptide 1 | 6.03 | 60356 | 221 | 5 | 17.8 | 0.14±0.03 | 0.15±0.1 | 1.04 |
| 88 | gi\|159164226 | Chain A, The Solution Structure Of The Second Thioredoxin Domain Of Human Protein Disulfide-Isomerase A3 | 5.27 | 15669 | 438 | 6 | 50.7 | 2.81±1.42 | 0±0 | 0.00 |
| 89 | gi\|538260950 | Chain A, Crystal Structure Of A0-domain Of P5 From H. Sapiens | 5.88 | 12786 | 240 | 5 | 46.6 | 1.04±0 | 0±0 | 0.00 |
| 90 | gi\|119597758 | dUTP pyrophosphatase, isoform CRA_d | 9.88 | 23028 | 260 | 5 | 31.7 | 0.89±0.12 | 0±0 | 0.00 |
| 91 | gi\|4501881 | actin, alpha skeletal muscle | 5.23 | 42024 | 542 | 6 | 30.2 | 0.71±0.21 | 0±0 | 0.00 |
| 92 | gi\|4501889 | actin, gamma-enteric smooth muscle isoform 1 precursor | 5.31 | 41850 | 532 | 6 | 29.3 | 0.71±0.21 | 0±0 | 0.00 |
| 93 | gi\|14625824 | moesin/anaplastic lymphoma kinase fusion protein | 7.61 | 61833 | 703 | 7 | 27.9 | 0.61±0.35 | 0±0 | 0.00 |

**Supplementary Table 1.** Altered phosphoproteins in antibody-depedent enhancement (ADE) of DENV2-infected U937 cells (cont.)

| **No.** | **NCBI ID** | **Protein** | | **pI** | | **MW (Da)** | | **Identification score** | | **No. of matched peptides** | | **%cov** | | **Mock (Mean±SD)** | | **Treated (Mean±SD)** | | **Ratio (Treated/Mock)** | |
| --- | --- | --- | --- | --- | --- | --- | --- | --- | --- | --- | --- | --- | --- | --- | --- | --- | --- | --- | --- |
| 94 | gi\|6457378 | cytovillin 2 | | 9.32 | | 16237 | | 326 | | 5 | | 49.6 | | 0.56±0.15 | | no emPAI | | n/a | |
| 95 | gi\|119630329 | chaperonin containing TCP1, subunit 8 (theta), isoform CRA_a | | 5.54 | | 59440 | | 510 | | 6 | | 33.5 | | 0.5±0.12 | | 0±0 | | 0.00 | |
| 96 | gi\|62421170 | actin-like protein | | 6.06 | | 11549 | | 96 | | 4 | | 24.3 | | 0.5±0.2 | | no emPAI | | n/a | |
| 97 | gi\|38014278 | TUBB3 protein, partial | | 4.9 | | 45593 | | 365 | | 6 | | 26.8 | | 0.47±0.05 | | 0±0 | | 0.00 | |
| 98 | gi\|178045 | gamma-actin, partial | | 5.65 | | 25862 | | 214 | | 5 | | 20.7 | | 0.45±0.18 | | no emPAI | | n/a | |
| 99 | gi\|671527 | gamma subunit of CCT chaperonin | | 6.23 | | 60292 | | 485 | | 6 | | 28.1 | | 0.44±0.24 | | no emPAI | | n/a | |
| 100 | gi\|6470150 | BiP protein, partial | | 5.23 | | 70888 | | 562 | | 6 | | 26.9 | | 0.31±0.06 | | 0±0 | | 0.00 | |
| 101 | gi\|193244897 | beta globin | | 6.17 | | 11479 | | 89 | | 3 | | 26.7 | | 0.3±0 | | 0±0 | | 0.00 | |
| 102 | gi\|1800303 | HIV-1 Nef interacting protein, partial | | 6.92 | | 45205 | | 200 | | 5 | | 20.6 | | 0.29±.0.04 | | 0±0 | | 0.00 | |
| 103 | gi\|4502643 | T-complex protein 1 subunit zeta isoform a | | 6.23 | | 57988 | | 304 | | 5 | | 21.1 | | 0.29±0.03 | | 0±0 | | 0.00 | |
| 104 | gi\|222476525 | 60 kDa chaperonin | | 7.93 | | 19902 | | 79 | | 2 | | 15.2 | | 0.27±0.1 | | no emPAI | | n/a | |
| 105 | gi\|197115528 | immunoglobulin heavy chain variable region | 8.96 | | 12966 | | 121 | | 5 | | 28.9 | | 0.26±0 | | no emPAI | | n/a | |  |
| 106 | gi\|4204880 | heat shock protein | 5.56 | | 69952 | | 335 | | 5 | | 15.2 | | 0.23±0.14 | | no emPAI | | n/a | |  |
| 107 | gi\|530417302 | PREDICTED: alpha-actinin-4 isoform X1 | 5.31 | | 104260 | | 581 | | 6 | | 24.3 | | 0.23±0.05 | | 0±0 | | 0.00 | |  |
| 108 | gi\|296080693 | glucose-6-phosphate isomerase isoform 1 | 9.01 | | 64284 | | 302 | | 5 | | 18.1 | | 0.23±13 | | 0±0 | | 0.00 | |  |
| 109 | gi\|15214751 | EEF1A1 protein, partial | 9.56 | | 17033 | | 97 | | 4 | | 13 | | 0.2±0 | | 0±0 | | 0.00 | |  |
| 110 | gi\|9507215 | tubulin alpha-8 chain isoform 1 | 4.94 | | 50062 | | 139 | | 6 | | 12 | | 0.18±0.04 | | 0±0 | | 0.00 | |  |
| 111 | gi\|46249758 | Ezrin | 5.94 | | 69199 | | 370 | | 6 | | 13.5 | | 0.17±0.07 | | 0±0 | | 0.00 | |  |
| 112 | gi\|190447 | prosomal protein P30-33K | 6.51 | | 30208 | | 127 | | 5 | | 15.2 | | 0.17±0.06 | | no emPAI | | n/a | |  |
| 113 | gi\|386781550 | radixin isoform 1 | 6.3 | | 71005 | | 336 | | 5 | | 10.3 | | 0.16±0.07 | | 0±0 | | 0.00 | |  |
| 114 | gi\|292659561 | Chain A, Crystal Structure Of The Complex Between The Bag5 Bd5 And Hsp70 Nbd | 6.38 | | 43068 | | 180 | | 6 | | 16.8 | | 0.16±0 | | no emPAI | | n/a | |  |

**Supplementary Table 1.** Altered phosphoproteins in antibody-depedent enhancement (ADE) of DENV2-infected U937 cells (cont.)

| **No.** | **NCBI ID** | **Protein** | | **pI** | | **MW (Da)** | | **Identification score** | | **No. of matched peptides** | | **%cov** | | **Mock (Mean±SD)** | | **Treated (Mean±SD)** | | **Ratio (Treated/Mock)** | |
| --- | --- | --- | --- | --- | --- | --- | --- | --- | --- | --- | --- | --- | --- | --- | --- | --- | --- | --- | --- |
| 115 | gi\|609342 | nucleophosmin-anaplastic lymphoma kinase fusion protein | 6.44 | | 75266 | | 120 | | 5 | | 10.4 | | 0.16±0.02 | | 0±0 | | 0.00 | |  |
| 116 | gi\|3712663 | DEAD-box protein | 6.37 | | 33095 | | 131 | | 4 | | 17.3 | | 0.16±0.06 | | no emPAI | | n/a | |  |
| 117 | gi\|24119203 | tropomyosin alpha-3 chain isoform Tpm3.1cy | 4.75 | | 29015 | | 166 | | 6 | | 27.8 | | 0.15±0.06 | | 0±0 | | 0.00 | |  |
| 118 | gi\|13529302 | GANAB protein, partial | 6.78 | | 34558 | | 129 | | 4 | | 16 | | 0.15±0.05 | | no emPAI | | n/a | |  |
| 119 | gi\|167887670 | proteasome subunit alpha type 7-like protein variant 1 | 9.14 | | 23789 | | 84 | | 3 | | 18.9 | | 0.14±0 | | 0±0 | | 0.00 | |  |
| 120 | gi\|426331707 | PREDICTED: tropomyosin alpha-3 chain isoform 6 [Gorilla gorilla gorilla] | 4.73 | | 33202 | | 120 | | 5 | | 14 | | 0.14±0.05 | | no emPAI | | n/a | |  |
| 121 | gi\|190613719 | Chain B, Crystal Structure Of A Complex Of Sse1p And Hsp70, Selenomethionine- Labeled Crystals | 6.4 | | 41904 | | 259 | | 5 | | 26.4 | | 0.13±0.04 | | no emPAI | | n/a | |  |
| 122 | gi\|260268505 | FLJ00343 protein | 5.77 | | 281288 | | 1018 | | 8 | | 17.6 | | 0.12±0.02 | | no emPAI | | n/a | |  |
| 123 | gi\|62897075 | heat shock 70kDa protein 9B precursor variant | 5.87 | | 73589 | | 317 | | 5 | | 18.7 | | 0.12±0.08 | | 0±0 | | 0.00 | |  |
| 124 | gi\|119576470 | hCG2010471 | 7.7 | | 32925 | | 78 | | 2 | | 5.2 | | 0.1±0 | | 0±0 | | 0.00 | |  |
| 125 | gi\|167614506 | plastin-2 | 5.29 | | 70244 | | 197 | | 5 | | 9.9 | | 0.1±0 | | 0±0 | | 0.00 | |  |
| 126 | gi\|9230777 | LIM protein ACT | 7.77 | | 32791 | | 73 | | 2 | | 18.3 | | 0.1±0 | | 0±0 | | 0.00 | |  |
| 127 | gi\|62089222 | heat shock 70kDa protein 1A variant | 5.97 | | 77448 | | 298 | | 5 | | 18.9 | | 0.09±0.04 | | 0±0 | | 0.00 | |  |
| 128 | gi\|68533125 | ACLY variant protein | 8.24 | | 124477 | | 328 | | 5 | | 12.5 | | 0.09±0.06 | | no emPAI | | n/a | |  |
| 129 | gi\|158518381 | RecName: Full=Putative heat shock 70 kDa protein 7; AltName: Full=Heat shock 70 kDa protein B | 7.72 | | 40220 | | 128 | | 5 | | 7.9 | | 0.08±0 | | 0±0 | | 0.00 | |  |
| 130 | gi\|206725454 | phosphatidylinositol 4-phosphate 5-kinase-like protein 1 isoform 1 | 9.63 | | 44544 | | 83 | | 3 | | 7.4 | | 0.07±0 | | 0±0 | | 0.00 | |  |
| 131 | gi\|578817058 | PREDICTED: phosphatidylinositol 4-phosphate 5-kinase-like protein 1 isoform X1 | 9.48 | | 53306 | | 95 | | 4 | | 8 | | 0.06±0 | | 0±0 | | 0.00 | |  |

**Supplementary Table 1.** Altered phosphoproteins in antibody-depedent enhancement (ADE) of DENV2-infected U937 cells (cont.)

| **No.** | **NCBI ID** | **Protein** | | | **pI** | | **MW (Da)** | | **Identification score** | | **No. of matched peptides** | | **%cov** | | **Mock (Mean±SD)** | | **Treated (Mean±SD)** | | **Ratio (Treated/Mock)** | |
| --- | --- | --- | --- | --- | --- | --- | --- | --- | --- | --- | --- | --- | --- | --- | --- | --- | --- | --- | --- | --- |
| 132 | gi\|88900491 | | neutral alpha-glucosidase AB isoform 3 precursor | 5.82 | | 109369 | | 164 | | 6 | | 10.4 | | 0.06±0 | | 0±0 | | 0.00 | |  |
| 133 | gi\|4507241 | | FACT complex subunit SSRP1 | 6.45 | | 81024 | | 87 | | 3 | | 7.5 | | 0.05±0.02 | | no emPAI | | n/a | |  |
| 134 | gi\|194018511 | | keratin, type II cytoskeletal 1b | 5.73 | | 61864 | | 108 | | 4 | | 11.4 | | 0.05±0 | | 0±0 | | 0.00 | |  |
| 135 | gi\|12666531 | | putative b,b-carotene-9~,10~-dioxygenase | 8.11 | | 62842 | | 75 | | 2 | | 3.6 | | 0.05±0 | | 0±0 | | 0.00 | |  |
| 136 | gi\|10863945 | | X-ray repair cross-complementing protein 5 | 5.55 | | 82652 | | 73 | | 2 | | 2.3 | | 0.04±0 | | 0±0 | | 0.00 | |  |
| 137 | gi\|38196957 | | FASN protein, partial | 6.22 | | 84158 | | 111 | | 4 | | 4.9 | | 0.04±0 | | 0±0 | | 0.00 | |  |
| 138 | gi\|62087882 | | heat shock 70kDa protein 4 isoform a variant | 5.44 | | 87949 | | 76 | | 2 | | 5.4 | | 0.04±0 | | no emPAI | | n/a | |  |
| 139 | gi\|33438760 | | myosin heavy chain | 5.76 | | 227863 | | 247 | | 5 | | 5.2 | | 0.03±0.02 | | 0±0 | | 0.00 | |  |
| 140 | gi\|20521736 | | KIAA1027 protein | 5.8 | | 270596 | | 257 | | 5 | | 7.1 | | 0.03±0.01 | | 0±0 | | 0.00 | |  |
| 141 | gi\|4235275 | | talin | 5.75 | | 269661 | | 252 | | 5 | | 6.7 | | 0.03±0.01 | | 0±0 | | 0.00 | |  |
| 142 | gi\|178058 | | alpha-actinin | 5.4 | | 103229 | | 143 | | 5 | | 4.7 | | 0.03±0 | | 0±0 | | 0.00 | |  |
| 143 | gi\|288562972 | | Chain A, Structure Of The Human Fatty Acid Synthase Ks-Mat Didomain As A Framework For Inhibitor Design. | 5.82 | | 104302 | | 102 | | 4 | | 5.3 | | 0.03±0 | | 0±0 | | 0.00 | |  |
| 144 | gi\|393007760 | | SND1-BRAF fusion | 9.36 | | 109079 | | 90 | | 3 | | 8.7 | | 0.03±0 | | 0±0 | | 0.00 | |  |
| 145 | gi\|4579911 | | apg-1 | 5.65 | | 94446 | | 142 | | 5 | | 9.4 | | 0.03±0 | | 0±0 | | 0.00 | |  |
| 146 | gi\|459352730 | | POTE ankyrin domain family member J | 5.66 | | 117315 | | 126 | | 5 | | 4.4 | | 0.03±0 | | 0±0 | | 0.00 | |  |
| 147 | gi\|77404397 | | staphylococcal nuclease domain-containing protein 1 | 6.74 | | 101934 | | 110 | | 4 | | 9 | | 0.03±0 | | 0±0 | | 0.00 | |  |
| 148 | gi\|799177 | | 100 kDa coactivator | 6.62 | | 99628 | | 95 | | 4 | | 8 | | 0.03±0 | | 0±0 | | 0.00 | |  |
| 149 | gi\|117606360 | | PH and SEC7 domain-containing protein 3 isoform a | 5.68 | | 115863 | | 98 | | 3 | | 5.4 | | 0.03±0 | | no emPAI | | n/a | |  |

**Supplementary Table 1.** Altered phosphoproteins in antibody-depedent enhancement (ADE) of DENV2-infected U937 cells (cont.)

| **No.** | **NCBI ID** | **Protein** | | **pI** | | **MW (Da)** | | **Identification score** | | **No. of matched peptides** | | **%cov** | | **Mock (Mean±SD)** | | **Treated (Mean±SD)** | | **Ratio (Treated/Mock)** | |
| --- | --- | --- | --- | --- | --- | --- | --- | --- | --- | --- | --- | --- | --- | --- | --- | --- | --- | --- | --- |
| 150 | gi\|4758282 | ephrin type-A receptor 7 isoform 1 precursor | 5.58 | | 112024 | | 95 | | 4 | | 6.3 | | 0.03±0.01 | | no emPAI | | n/a | |  |
| 151 | gi\|30026465 | pol protein [Human endogenous retrovirus HCML-ARV] | 9.22 | | 131387 | | 70 | | 2 | | 3.9 | | 0.02±0 | | 0±0 | | 0.00 | |  |
| 152 | gi\|27451602 | tau-tubulin kinase | 6.58 | | 182353 | | 112 | | 5 | | 4.1 | | 0.02±0 | | no emPAI | | n/a | |  |
| 153 | gi\|530405585 | PREDICTED: dmX-like protein 2 isoform X3 | 5.88 | | 291483 | | 84 | | 3 | | 2.1 | | 0.01±0 | | 0±0 | | 0.00 | |  |
| 154 | gi\|348579692 | PREDICTED: tropomyosin alpha-3 chain isoformX4 [Cavia porcellus] | 4.7 | | 28761 | | 147 | | 5 | | 29.4 | | no emPAI | | 0±0 | | n/a | |  |
| 155 | gi\|426331711 | PREDICTED: tropomyosin alpha-3 chain isoform 8 [Gorilla gorilla gorilla] | 4.7 | | 33110 | | 101 | | 4 | | 15.4 | | no emPAI | | 0±0 | | n/a | |  |
| 156 | gi\|1710248 | protein disulfide isomerase-related protein 5 | 4.95 | | 46170 | | 772 | | 7 | | 31.8 | | 1.42±0.3 | | 1.34±0.45 | | 0.94 | |  |
| 157 | gi\|729433 | RecName: Full=Protein disulfide-isomerase A3; AltName: Full=58 kDa glucose-regulated protein; AltName: Full=58 kDa microsomal protein; Short=p58; AltName: Full=Disulfide isomerase ER-60; AltName: Full=Endoplasmic reticulum resident protein 57; Short | 6.23 | | 56894 | | 763 | | 7 | | 30.1 | | 1.09±0.46 | | 1.03±0.33 | | 0.94 | |  |
| 158 | gi\|220702506 | Chain A, TapasinERP57 HETERODIMER | 5.61 | | 54199 | | 1214 | | 8 | | 47 | | 2.7±1.36 | | 2.46±0.93 | | 0.91 | |  |
| 159 | gi\|395759492 | Chain A, Crystal Structure Of A Heat Shock 70kda Protein 2 (Hspa2) From Homo Sapiens At 1.80 A Resolution | 7.18 | | 42130 | | 337 | | 5 | | 31.5 | | 0.58±0.12 | | 0.53±0.18 | | 0.91 | |  |
| 160 | gi\|114794262 | Chain A, Crystal Structure Of The Bb~ Fragment Of Erp57 | 5.49 | | 28426 | | 316 | | 5 | | 33.7 | | 0.99±0.62 | | 0.83±0.33 | | 0.84 | |  |
| 161 | gi\|5729953 | nuclear migration protein nudC | 5.27 | | 38219 | | 165 | | 6 | | 12.1 | | 0.19±0.1 | | 0.15±0.04 | | 0.81 | |  |
| 162 | gi\|12667788 | myosin-9 | 5.5 | | 226392 | | 1008 | | 8 | | 15.7 | | 0.32±0.22 | | 0.25±0.17 | | 0.76 | |  |

**Supplementary Table 1.** Altered phosphoproteins in antibody-depedent enhancement (ADE) of DENV2-infected U937 cells (cont.)

| **No.** | **NCBI ID** | **Protein** | | **pI** | | **MW (Da)** | | **Identification score** | | **No. of matched peptides** | | **%cov** | | **Mock (Mean±SD)** | | **Treated (Mean±SD)** | | **Ratio (Treated/Mock)** | |
| --- | --- | --- | --- | --- | --- | --- | --- | --- | --- | --- | --- | --- | --- | --- | --- | --- | --- | --- | --- |
| 163 | gi\|159162689 | Chain A, Human Protein Disulfide Isomerase, Nmr, 40 Structures | 5.94 | | 13249 | | 433 | | 6 | | 70.8 | | 6.13±4.15 | | 4.66±3.3 | | 0.76 | |  |
| 164 | gi\|5729877 | heat shock cognate 71 kDa protein isoform 1 | 5.37 | | 70854 | | 986 | | 7 | | 33.7 | | 1.33±0.27 | | 0.95±0.67 | | 0.72 | |  |
| 165 | gi\|2443580 | dUTPase | 9.65 | | 26690 | | 283 | | 5 | | 40.1 | | 1.04±0.2 | | 0.73±0.31 | | 0.70 | |  |
| 166 | gi\|5803187 | transaldolase | 6.36 | | 37516 | | 282 | | 5 | | 29.4 | | 0.77±0.47 | | 0.53±0.13 | | 0.69 | |  |
| 167 | gi\|5902134 | coronin-1A | 6.25 | | 50994 | | 227 | | 5 | | 9.8 | | 0.37±0.09 | | 0.25±0.04 | | 0.66 | |  |
| 168 | gi\|4529892 | HSP70-2 | 5.48 | | 69982 | | 202 | | 5 | | 12.9 | | 0.13±0.03 | | 0.08±0.03 | | 0.60 | |  |
| 169 | gi\|62088022 | hypothetical protein FLJ41407 variant | 8.54 | | 59576 | | 198 | | 5 | | 8.8 | | 0.24±0.07 | | 0.14±0.03 | | 0.58 | |  |
| 170 | gi\|4503143 | cathepsin D preproprotein | 6.1 | | 44524 | | 116 | | 5 | | 15.3 | | 0.27±0.04 | | 0.15±0 | | 0.56 | |  |
| 171 | gi\|5453549 | peroxiredoxin-4 precursor | 5.86 | | 30521 | | 188 | | 6 | | 18.1 | | 0.36±0 | | 0.19±0.06 | | 0.53 | |  |
| 172 | gi\|4503423 | deoxyuridine 5~-triphosphate nucleotidohydrolase, mitochondrial isoform 2 | 6.15 | | 17737 | | 299 | | 5 | | 62.2 | | 1.87±0.4 | | 0.98±0.62 | | 0.53 | |  |
| 173 | gi\|31645 | glyceraldehyde-3-phosphate dehydrogenase | 8.26 | | 36031 | | 117 | | 5 | | 13.7 | | 0.44±0.25 | | 0.09±0 | | 0.20 | |  |
| 174 | gi\|10039443 | NEDD4-like ubiquitin ligase 1 | 5.3 | | 177156 | | 73 | | 2 | | 4.7 | | 0±0 | | no emPAI | | n/a | |  |
| 175 | gi\|10047303 | KIAA1614 protein | 9.14 | | 128609 | | 82 | | 3 | | 3.1 | | 0±0 | | no emPAI | | n/a | |  |
| 176 | gi\|10092601 | eukaryotic translation initiation factor 4 gamma 3 isoform 3 | 5.27 | | 176542 | | 106 | | 4 | | 3.8 | | 0±0 | | no emPAI | | n/a | |  |
| 177 | gi\|10443222 | CDC2L5 protein kinase | 9.7 | | 164870 | | 86 | | 3 | | 5.4 | | no emPAI | | 0±0 | | n/a | |  |
| 178 | gi\|10636871 | immunoglobulin heavy chain variable region | 8.84 | | 13187 | | 115 | | 4 | | 27.7 | | 0±0 | | no emPAI | | n/a | |  |
| 179 | gi\|109658672 | Gen homolog 1, endonuclease (Drosophila) | 8.04 | | 102872 | | 67 | | 2 | | 5.6 | | no emPAI | | 0±0 | | n/a | |  |
| 180 | gi\|109732069 | Dual oxidase 1 | 8.22 | | 177210 | | 77 | | 2 | | 4.2 | | 0±0 | | no emPAI | | n/a | |  |
| 181 | gi\|110347427 | ubiquitin carboxyl-terminal hydrolase 34 | 5.51 | | 403973 | | 101 | | 4 | | 3.5 | | no emPAI | | 0±0 | | n/a | |  |

**Supplementary Table 1.** Altered phosphoproteins in antibody-depedent enhancement (ADE) of DENV2-infected U937 cells (cont.)

| **No.** | **NCBI ID** | **Protein** | | | **pI** | | **MW (Da)** | | **Identification score** | | **No. of matched peptides** | | **%cov** | | **Mock (Mean±SD)** | | **Treated (Mean±SD)** | | **Ratio (Treated/Mock)** | |
| --- | --- | --- | --- | --- | --- | --- | --- | --- | --- | --- | --- | --- | --- | --- | --- | --- | --- | --- | --- | --- |
| 182 | gi\|110590597 | | Chain A, Apo-Human Serum Transferrin (Non-Glycosylated) | 6.58 | | 74643 | | 78 | | 3 | | 13.5 | | 0±0 | | no emPAI | | n/a | |  |
| 183 | gi\|110735402 | | receptor-type tyrosine-protein phosphatase U isoform 3 precursor | 6.46 | | 162320 | | 96 | | 4 | | 6.2 | | no emPAI | | 0±0 | | n/a | |  |
| 184 | gi\|110735439 | | Werner syndrome ATP-dependent helicase | 5.96 | | 162357 | | 107 | | 4 | | 6 | | 0±0 | | no emPAI | | n/a | |  |
| 185 | gi\|110735441 | | A disintegrin and metalloproteinase with thrombospondin motifs 16 preproprotein | 8.99 | | 136114 | | 103 | | 4 | | 9.1 | | no emPAI | | 0±0 | | n/a | |  |
| 186 | gi\|111548668 | | zinc finger protein 676 | 9.1 | | 67580 | | 66 | | 2 | | 10.7 | | 0±0 | | no emPAI | | n/a | |  |
| 187 | gi\|112420968 | | coiled-coil domain-containing protein 158 | 6.08 | | 127062 | | 86 | | 3 | | 4.9 | | no emPAI | | 0±0 | | n/a | |  |
| 188 | gi\|11244871 | | dioxin receptor repressor | 9.09 | | 77771 | | 68 | | 2 | | 10.1 | | 0±0 | | no emPAI | | n/a | |  |
| 189 | gi\|11275980 | | NOTCH 1 | 4.99 | | 272372 | | 80 | | 3 | | 3.7 | | no emPAI | | 0±0 | | n/a | |  |
| 190 | gi\|113201774 | | PAX5/ZNF521 fusion protein | 7.2 | | 172380 | | 101 | | 4 | | 6.9 | | no emPAI | | 0±0 | | n/a | |  |
| 191 | gi\|11321634 | | CD2-associated protein | 6.07 | | 71407 | | 88 | | 3 | | 11.9 | | no emPAI | | 0±0 | | n/a | |  |
| 192 | gi\|11344951 | | FYVE-finger-containing Rab5 effector protein Rabenosyn-5 | 5.36 | | 88747 | | 114 | | 4 | | 8.4 | | 0±0 | | no emPAI | | n/a | |  |
| 193 | gi\|11385642 | | CTCL tumor antigen se1-1 | 4.87 | | 80249 | | 84 | | 3 | | 7.9 | | no emPAI | | 0±0 | | n/a | |  |
| 194 | gi\|1142657 | | X2 box repressor | 6.99 | | 121063 | | 68 | | 2 | | 5 | | 0±0 | | no emPAI | | n/a | |  |
| 195 | gi\|114581541 | | PREDICTED: nostrin isoform X1 [Pan troglodytes] | 9.03 | | 57696 | | 66 | | 2 | | 10.7 | | no emPAI | | 0±0 | | n/a | |  |
| 196 | gi\|114627088 | | PREDICTED: TBC1 domain family member 13 isoform X1 [Pan troglodytes] | 5.11 | | 46496 | | 67 | | 2 | | 11.3 | | no emPAI | | 0±0 | | n/a | |  |
| 197 | gi\|114794705 | | Chain A, Crystal Structure Of An Aspartoacylase From Homo Sapiens | 6.07 | | 35716 | | 70 | | 2 | | 3.5 | | 0±0 | | no emPAI | | n/a | |  |
| 198 | gi\|115430110 | | protein FAM81B | 9.15 | | 51990 | | 70 | | 2 | | 9.7 | | 0±0 | | no emPAI | | n/a | |  |

**Supplementary Table 1.** Altered phosphoproteins in antibody-depedent enhancement (ADE) of DENV2-infected U937 cells (cont.)

| **No.** | **NCBI ID** | **Protein** | | **pI** | | **MW (Da)** | | **Identification score** | | **No. of matched peptides** | | **%cov** | | **Mock (Mean±SD)** | | **Treated (Mean±SD)** | | **Ratio (Treated/Mock)** | |
| --- | --- | --- | --- | --- | --- | --- | --- | --- | --- | --- | --- | --- | --- | --- | --- | --- | --- | --- | --- |
| 199 | gi\|115502126 | RecName: Full=Putative uncharacterized protein MRGPRG-AS1; AltName: Full=MRGPRG antisense RNA 1; AltName: Full=MRGPRG antisense gene protein 1 | 6.69 | | 16446 | | 72 | | 2 | | 15.2 | | no emPAI | | 0±0 | | n/a | |  |
| 200 | gi\|116235015 | NEF-associated factor 1 | 7.6 | | 62596 | | 135 | | 6 | | 14.9 | | no emPAI | | 0±0 | | n/a | |  |
| 201 | gi\|116283334 | BCAS1 protein | 8.8 | | 41542 | | 114 | | 4 | | 17.4 | | no emPAI | | 0±0 | | n/a | |  |
| 202 | gi\|11641411 | M-phase inducer phosphatase 2 isoform 3 | 5.87 | | 60717 | | 68 | | 2 | | 10.2 | | 0±0 | | no emPAI | | n/a | |  |
| 203 | gi\|116496599 | RBM44 protein | 7.72 | | 46657 | | 83 | | 3 | | 20.1 | | no emPAI | | 0±0 | | n/a | |  |
| 204 | gi\|1165219 | cell adhesion kinase beta | 5.91 | | 115752 | | 89 | | 3 | | 7.4 | | no emPAI | | 0±0 | | n/a | |  |
| 205 | gi\|116536083 | dual 3~,5~-cyclic-AMP and -GMP phosphodiesterase 11A isoform 1 | 5.56 | | 55664 | | 77 | | 2 | | 9 | | no emPAI | | 0±0 | | n/a | |  |
| 206 | gi\|116536085 | dual 3~,5~-cyclic-AMP and -GMP phosphodiesterase 11A isoform 4 | 6.15 | | 104685 | | 73 | | 2 | | 4.7 | | no emPAI | | 0±0 | | n/a | |  |
| 207 | gi\|116812597 | zinc finger protein 586 isoform a | 9.14 | | 46384 | | 73 | | 2 | | 15.9 | | no emPAI | | 0±0 | | n/a | |  |
| 208 | gi\|117168002 | DUSP27 protein, partial | 5.1 | | 129967 | | 73 | | 2 | | 4.8 | | no emPAI | | 0±0 | | n/a | |  |
| 209 | gi\|117606351 | F-box only protein 43 | 8.4 | | 78354 | | 70 | | 2 | | 7.2 | | 0±0 | | no emPAI | | n/a | |  |
| 210 | gi\|117676384 | protein SCAF11 | 8.69 | | 164551 | | 108 | | 4 | | 7.9 | | 0±0 | | no emPAI | | n/a | |  |
| 211 | gi\|1181079 | diacylglycerol kinase delta | 7.46 | | 130072 | | 114 | | 4 | | 8.6 | | 0±0 | | no emPAI | | n/a | |  |
| 212 | gi\|118197272 | autophagy-related protein 2 homolog B | 5.49 | | 232604 | | 92 | | 3 | | 3.5 | | no emPAI | | 0±0 | | n/a | |  |
| 213 | gi\|1185071 | bcr/abl fusion protein, partial | 8.89 | | 25406 | | 69 | | 2 | | 18.2 | | 0±0 | | no emPAI | | n/a | |  |
| 214 | gi\|11863152 | protein Jumonji isoform 1 | 9.46 | | 138648 | | 101 | | 5 | | 7 | | 0±0 | | no emPAI | | n/a | |  |
| 215 | gi\|11863156 | reversion-inducing cysteine-rich protein with Kazal motifs precursor | 6.35 | | 106386 | | 79 | | 2 | | 5 | | no emPAI | | 0±0 | | n/a | |  |
| 216 | gi\|118764173 | TDRD9 protein | 6.14 | | 99528 | | 81 | | 2 | | 8 | | no emPAI | | 0±0 | | n/a | |  |
| 217 | gi\|119568122 | spectrin repeat containing, nuclear envelope 1, isoform CRA_k | 5.38 | | 1007974 | | 173 | | 8 | | 3.4 | | 0±0 | | no emPAI | | n/a | |  |

**Supplementary Table 1.** Altered phosphoproteins in antibody-depedent enhancement (ADE) of DENV2-infected U937 cells (cont.)

| **No.** | **NCBI ID** | **Protein** | | **pI** | | **MW (Da)** | | **Identification score** | | **No. of matched peptides** | | **%cov** | | **Mock (Mean±SD)** | | **Treated (Mean±SD)** | | **Ratio (Treated/Mock)** | |
| --- | --- | --- | --- | --- | --- | --- | --- | --- | --- | --- | --- | --- | --- | --- | --- | --- | --- | --- | --- |
| 218 | gi\|119568230 | utrophin (homologous to dystrophin), isoform CRA_a | 5.18 | | 387947 | | 126 | | 5 | | 3.5 | | 0±0 | | no emPAI | | n/a | |  |
| 219 | gi\|119569073 | inhibitor of Bruton agammaglobulinemia tyrosine kinase, isoform CRA_d | 8.32 | | 132649 | | 78 | | 3 | | 6.3 | | 0±0 | | no emPAI | | n/a | |  |
| 220 | gi\|119569579 | dedicator of cytokinesis 1 | 7.27 | | 202016 | | 93 | | 4 | | 5.8 | | no emPAI | | 0±0 | | n/a | |  |
| 221 | gi\|119569886 | nebulin-related anchoring protein, isoform CRA_c | 9.36 | | 166110 | | 78 | | 3 | | 6.4 | | no emPAI | | 0±0 | | n/a | |  |
| 222 | gi\|119570064 | tripartite motif-containing 8, isoform CRA_a | 9.41 | | 52024 | | 105 | | 4 | | 15.4 | | no emPAI | | 0±0 | | n/a | |  |
| 223 | gi\|119570769 | hCG2044987 | 9.73 | | 11415 | | 73 | | 2 | | 43.7 | | 0±0 | | no emPAI | | n/a | |  |
| 224 | gi\|119571010 | hypothetical protein DKFZp434N035, isoform CRA_c | 11.2 | | 32820 | | 71 | | 2 | | 20.5 | | 0±0 | | no emPAI | | n/a | |  |
| 225 | gi\|119572228 | hCG2002991, isoform CRA_a, partial | 9.84 | | 108361 | | 68 | | 2 | | 2.2 | | 0±0 | | no emPAI | | n/a | |  |
| 226 | gi\|119572466 | tudor domain containing 3, isoform CRA_b | 9.16 | | 84035 | | 89 | | 4 | | 11.7 | | no emPAI | | 0±0 | | n/a | |  |
| 227 | gi\|119572703 | hypothetical protein LOC374920 | 8.44 | | 113765 | | 74 | | 2 | | 6.5 | | 0±0 | | no emPAI | | n/a | |  |
| 228 | gi\|119573330 | IQ motif containing GTPase activating protein 3, isoform CRA_a | 7.21 | | 179287 | | 67 | | 2 | | 2 | | 0±0 | | no emPAI | | n/a | |  |
| 229 | gi\|119573381 | lamin A/C, isoform CRA_a | 9 | | 78316 | | 97 | | 4 | | 13.3 | | 0±0 | | no emPAI | | n/a | |  |
| 230 | gi\|119574269 | GRIP and coiled-coil domain containing 2, isoform CRA_b | 4.95 | | 122142 | | 75 | | 2 | | 6.1 | | no emPAI | | 0±0 | | n/a | |  |
| 231 | gi\|119574357 | hCG1990594, isoform CRA_b | 6.1 | | 537722 | | 131 | | 6 | | 3.4 | | 0±0 | | 0±0 | | n/a | |  |
| 232 | gi\|119574585 | ankyrin 3, node of Ranvier (ankyrin G), isoform CRA_b | 5.96 | | 464238 | | 93 | | 2 | | 3.5 | | 0±0 | | 0±0 | | n/a | |  |
| 233 | gi\|119575196 | checkpoint with forkhead and ring finger domains, isoform CRA_a | 8.5 | | 49319 | | 74 | | 3 | | 13.7 | | no emPAI | | 0±0 | | n/a | |  |
| 234 | gi\|119575200 | checkpoint with forkhead and ring finger domains, isoform CRA_e | 8.5 | | 50674 | | 76 | | 2 | | 10.3 | | 0±0 | | no emPAI | | n/a | |  |

**Supplementary Table 1.** Altered phosphoproteins in antibody-depedent enhancement (ADE) of DENV2-infected U937 cells (cont.)

| **No.** | **NCBI ID** | **Protein** | **pI** | **MW (Da)** | **Identification score** | **No. of matched peptides** | **%cov** | **Mock (Mean±SD)** | **Treated (Mean±SD)** | **Ratio (Treated/Mock)** |
| --- | --- | --- | --- | --- | --- | --- | --- | --- | --- | --- |
| 235 | gi\|119575716 | human immunodeficiency virus type I enhancer binding protein 1, isoform CRA_d | 8.58 | 227217 | 76 | 2 | 3.6 | 0±0 | no emPAI | n/a |
| 236 | gi\|119575792 | kinesin family member 13A, isoform CRA_b | 5.34 | 194884 | 77 | 2 | 5.7 | 0±0 | no emPAI | n/a |
| 237 | gi\|119576092 | sterol regulatory element binding transcription factor 1, isoform CRA_a | 8.06 | 113981 | 79 | 2 | 3.3 | 0±0 | no emPAI | n/a |
| 238 | gi\|119576240 | ubiquitin specific peptidase 31, isoform CRA_b | 9.63 | 98974 | 112 | 4 | 8.3 | no emPAI | 0±0 | n/a |
| 239 | gi\|119577200 | ryanodine receptor 1 (skeletal), isoform CRA_b | 5.17 | 560473 | 108 | 4 | 2.5 | 0±0 | no emPAI | n/a |
| 240 | gi\|119577892 | hCG1995737 | 9.3 | 12176 | 72 | 2 | 34.2 | 0±0 | no emPAI | n/a |
| 241 | gi\|119578233 | PDZ domain containing RING finger 4, isoform CRA_c | 5.1 | 89041 | 68 | 2 | 8.5 | 0±0 | no emPAI | n/a |
| 242 | gi\|119578788 | unc-13 homolog B (C. elegans), isoform CRA_b | 5.64 | 220173 | 172 | 8 | 7.4 | 0±0 | no emPAI | n/a |
| 243 | gi\|119579061 | hCG1818607, isoform CRA_c | 8.18 | 193284 | 82 | 3 | 4.4 | 0±0 | no emPAI | n/a |
| 244 | gi\|119579134 | jumonji domain containing 2C, isoform CRA_a | 5.92 | 118069 | 72 | 2 | 3.6 | 0±0 | no emPAI | n/a |
| 245 | gi\|119579276 | tripartite motif-containing 14, isoform CRA_c | 7.6 | 39649 | 84 | 3 | 15.2 | 0±0 | no emPAI | n/a |
| 246 | gi\|119579455 | hCG28765, isoform CRA_b | 4.94 | 147137 | 112 | 4 | 6.9 | 0±0 | no emPAI | n/a |
| 247 | gi\|119579555 | hCG1798659 | 9.83 | 28893 | 74 | 2 | 15.4 | no emPAI | 0±0 | n/a |
| 248 | gi\|119579737 | zinc finger protein 81 (HFZ20), isoform CRA_b, partial | 8.78 | 82060 | 67 | 2 | 5.3 | no emPAI | 0±0 | n/a |
| 249 | gi\|119580396 | hypothetical protein MGC50372, isoform CRA_a | 6.24 | 219528 | 77 | 2 | 4.2 | 0±0 | no emPAI | n/a |
| 250 | gi\|119580917 | hCG2007242, isoform CRA_a | 9.18 | 128699 | 140 | 7 | 8.2 | 0±0 | no emPAI | n/a |

**Supplementary Table 1.** Altered phosphoproteins in antibody-depedent enhancement (ADE) of DENV2-infected U937 cells (cont.)

| **No.** | **NCBI ID** | **Protein** | **pI** | **MW (Da)** | **Identification score** | **No. of matched peptides** | **%cov** | **Mock (Mean±SD)** | **Treated (Mean±SD)** | **Ratio (Treated/Mock)** |
| --- | --- | --- | --- | --- | --- | --- | --- | --- | --- | --- |
| 251 | gi\|119582901 | MAM domain containing 2, isoform CRA_e, partial | 6.15 | 91022 | 85 | 3 | 9.7 | no emPAI | 0±0 | n/a |
| 252 | gi\|119583753 | RAB11 family interacting protein 1 (class I), isoform CRA_c | 9.31 | 70810 | 70 | 2 | 9.6 | no emPAI | 0±0 | n/a |
| 253 | gi\|119584770 | N-glycanase 1, isoform CRA_e | 8.93 | 77120 | 68 | 2 | 5.3 | 0±0 | no emPAI | n/a |
| 254 | gi\|119584937 | xylulokinase homolog (H. influenzae), isoform CRA_c | 5.75 | 49642 | 85 | 3 | 17.9 | 0±0 | no emPAI | n/a |
| 255 | gi\|119585179 | ALS2 C-terminal like, isoform CRA_a | 8.89 | 59345 | 67 | 2 | 9.3 | 0±0 | no emPAI | n/a |
| 256 | gi\|119585298 | collagen, type VII, alpha 1 (epidermolysis bullosa, dystrophic, dominant and recessive), isoform CRA_b | 6.34 | 260960 | 93 | 4 | 2.8 | 0±0 | no emPAI | n/a |
| 257 | gi\|119585727 | chromosome 3 open reading frame 63, isoform CRA_e | 5.57 | 188969 | 90 | 3 | 4.8 | 0±0 | no emPAI | n/a |
| 258 | gi\|119585758 | filamin B, beta (actin binding protein 278), isoform CRA_c | 5.36 | 263856 | 75 | 2 | 4.7 | no emPAI | 0±0 | n/a |
| 259 | gi\|119585791 | FLJ42117 protein, isoform CRA_e | 6.08 | 82753 | 88 | 3 | 7.8 | no emPAI | 0±0 | n/a |
| 260 | gi\|119585810 | Ca2+-dependent secretion activator, isoform CRA_b | 5.92 | 141296 | 74 | 2 | 4.8 | no emPAI | 0±0 | n/a |
| 261 | gi\|119585838 | ADAM metallopeptidase with thrombospondin type 1 motif, 9, isoform CRA_a | 7.71 | 186945 | 85 | 3 | 5.6 | no emPAI | 0±0 | n/a |
| 262 | gi\|119585936 | PDZ domain containing RING finger 3, isoform CRA_b | 5.04 | 89116 | 71 | 2 | 5.4 | no emPAI | 0±0 | n/a |
| 263 | gi\|119586923 | rotatin, isoform CRA_d | 6.27 | 247740 | 69 | 2 | 2.6 | no emPAI | 0±0 | n/a |
| 264 | gi\|119587176 | hCG1980844, isoform CRA_h | 7.89 | 270861 | 71 | 2 | 2.3 | no emPAI | 0±0 | n/a |
| 265 | gi\|119587912 | Rho guanine nucleotide exchange factor (GEF) 12, isoform CRA_a | 5.51 | 174512 | 77 | 2 | 6.7 | no emPAI | 0±0 | n/a |
| 266 | gi\|119587942 | hCG1647980, partial | 10.61 | 67787 | 66 | 2 | 8.6 | 0±0 | no emPAI | n/a |

**Supplementary Table 1.** Altered phosphoproteins in antibody-depedent enhancement (ADE) of DENV2-infected U937 cells (cont.)

| **No.** | **NCBI ID** | **Protein** | **pI** | **MW (Da)** | **Identification score** | **No. of matched peptides** | **%cov** | **Mock (Mean±SD)** | **Treated (Mean±SD)** | **Ratio (Treated/Mock)** |
| --- | --- | --- | --- | --- | --- | --- | --- | --- | --- | --- |
| 267 | gi\|119588750 | neuron navigator 2, isoform CRA_b | 9.04 | 257537 | 116 | 4 | 5.7 | no emPAI | 0±0 | n/a |
| 268 | gi\|119589500 | hypothetical protein MGC34725, isoform CRA_b | 9.82 | 26380 | 78 | 3 | 25.6 | no emPAI | 0±0 | n/a |
| 269 | gi\|119590685 | amyotrophic lateral sclerosis 2 (juvenile) chromosome region, candidate 11, isoform CRA_c, partial | 7.34 | 208871 | 116 | 4 | 6.1 | no emPAI | 0±0 | n/a |
| 270 | gi\|119590707 | hCG1776789, isoform CRA_a, partial | 8.47 | 117071 | 105 | 4 | 7.1 | 0±0 | no emPAI | n/a |
| 271 | gi\|119591149 | aortic preferentially expressed gene 1, isoform CRA_b, partial | 9.99 | 94917 | 69 | 2 | 6.5 | 0±0 | no emPAI | n/a |
| 272 | gi\|119591469 | hCG2012694, isoform CRA_b | 6.38 | 193921 | 72 | 2 | 3.3 | no emPAI | 0±0 | n/a |
| 273 | gi\|119591597 | ankyrin repeat and MYND domain containing 1, isoform CRA_e | 6.19 | 122863 | 96 | 4 | 5.4 | no emPAI | 0±0 | n/a |
| 274 | gi\|119592047 | kinesin family member 1B, isoform CRA_a | 5.42 | 188346 | 103 | 4 | 4.8 | 0±0 | no emPAI | n/a |
| 275 | gi\|119592487 | hCG37184, isoform CRA_a | 9.48 | 75693 | 72 | 2 | 9.8 | no emPAI | 0±0 | n/a |
| 276 | gi\|119594765 | synovial apoptosis inhibitor 1, synoviolin, isoform CRA_c | 5.93 | 41296 | 66 | 2 | 8.5 | no emPAI | 0±0 | n/a |
| 277 | gi\|119595177 | SH3 and multiple ankyrin repeat domains 2, isoform CRA_a | 6.02 | 178068 | 96 | 4 | 4.1 | no emPAI | 0±0 | n/a |
| 278 | gi\|119595335 | hCG1811370 | 6.54 | 126254 | 95 | 4 | 6.7 | 0±0 | no emPAI | n/a |
| 279 | gi\|119595779 | laminin, alpha 5, isoform CRA_b | 6.59 | 402001 | 143 | 6 | 4.9 | 0±0 | no emPAI | n/a |
| 280 | gi\|119596116 | protein kinase C binding protein 1, isoform CRA_g | 6.21 | 131336 | 108 | 4 | 6.7 | no emPAI | 0±0 | n/a |
| 281 | gi\|119597125 | lemur tyrosine kinase 2, isoform CRA_a | 4.64 | 204081 | 96 | 4 | 4.3 | no emPAI | 0±0 | n/a |
| 282 | gi\|119597267 | A kinase (PRKA) anchor protein (yotiao) 9, isoform CRA_b | 5.06 | 245678 | 125 | 5 | 4.2 | no emPAI | 0±0 | n/a |
| 283 | gi\|119597269 | A kinase (PRKA) anchor protein (yotiao) 9, isoform CRA_d | 4.95 | 455804 | 169 | 8 | 3.5 | 0±0 | no emPAI | n/a |

**Supplementary Table 1.** Altered phosphoproteins in antibody-depedent enhancement (ADE) of DENV2-infected U937 cells (cont.)

| **No.** | **NCBI ID** | | **Protein** | | **pI** | | **MW (Da)** | | **Identification score** | | **No. of matched peptides** | | **%cov** | | **Mock (Mean±SD)** | | **Treated (Mean±SD)** | | **Ratio (Treated/Mock)** | |
| --- | --- | --- | --- | --- | --- | --- | --- | --- | --- | --- | --- | --- | --- | --- | --- | --- | --- | --- | --- | --- |
| 284 | gi\|119597958 | SAFB-like, transcription modulator, isoform CRA_b | | 9.35 | | 131534 | | 153 | | 6 | | 9.8 | | 0±0 | | no emPAI | | n/a | |  |
| 285 | gi\|119598044 | lactamase, beta, isoform CRA_b | | 8.68 | | 60655 | | 73 | | 2 | | 11.5 | | 0±0 | | no emPAI | | n/a | |  |
| 286 | gi\|119598094 | zinc finger protein 609, isoform CRA_c | | 8.69 | | 145865 | | 91 | | 3 | | 4.2 | | no emPAI | | 0±0 | | n/a | |  |
| 287 | gi\|119598622 | SUMO1/sentrin/SMT3 specific peptidase 2, isoform CRA_c | | 9.37 | | 58488 | | 80 | | 3 | | 6.3 | | no emPAI | | 0±0 | | n/a | |  |
| 288 | gi\|119598780 | hCG16256, isoform CRA_a | | 6.26 | | 119256 | | 108 | | 4 | | 7.6 | | 0±0 | | no emPAI | | n/a | |  |
| 289 | gi\|119598995 | hypothetical protein FLJ23049 | | 5.23 | | 92282 | | 83 | | 3 | | 9.1 | | 0±0 | | no emPAI | | n/a | |  |
| 290 | gi\|119599362 | hCG27481, isoform CRA_b | | 8.6 | | 115190 | | 72 | | 2 | | 7.2 | | no emPAI | | 0±0 | | n/a | |  |
| 291 | gi\|119599593 | nephronophthisis 3 (adolescent) | | 6.65 | | 116331 | | 69 | | 2 | | 8.7 | | 0±0 | | no emPAI | | n/a | |  |
| 292 | gi\|119600833 | hCG2044005 | | 11.58 | | 26057 | | 67 | | 2 | | 21.2 | | 0±0 | | no emPAI | | n/a | |  |
| 293 | gi\|119600956 | LIM domain 7, isoform CRA_b | | 8.16 | | 156637 | | 82 | | 3 | | 6 | | no emPAI | | 0±0 | | n/a | |  |
| 294 | gi\|119601372 | hCG1796718, partial | | 9.06 | | 17856 | | 71 | | 2 | | 19.9 | | 0±0 | | no emPAI | | n/a | |  |
| 295 | gi\|119601857 | hCG21479, isoform CRA_b | | 5.87 | | 228075 | | 121 | | 5 | | 3.7 | | no emPAI | | 0±0 | | n/a | |  |
| 296 | gi\|119601937 | KIAA1409 | | 5.79 | | 298705 | | 90 | | 3 | | 2.1 | | 0±0 | | 0±0 | | n/a | |  |
| 297 | gi\|119602039 | chromosome 14 open reading frame 103 | | 5.24 | | 172400 | | 73 | | 2 | | 4.3 | | no emPAI | | 0±0 | | n/a | |  |
| 298 | gi\|119602165 | dynein, cytoplasmic 1, heavy chain 1, isoform CRA_a, partial | | 6.12 | | 525614 | | 79 | | 2 | | 2.7 | | no emPAI | | 0±0 | | n/a | |  |
| 299 | gi\|119602167 | dynein, cytoplasmic 1, heavy chain 1, isoform CRA_c | | 5.97 | | 485655 | | 115 | | 4 | | 3.4 | | 0±0 | | no emPAI | | n/a | |  |
| 300 | gi\|119603141 | zinc finger protein 423 | | 7.82 | | 136152 | | 78 | | 3 | | 6.1 | | no emPAI | | 0±0 | | n/a | |  |
| 301 | gi\|119603200 | chromodomain helicase DNA binding protein 9, isoform CRA_b | | 6.57 | | 314490 | | 73 | | 2 | | 2.9 | | no emPAI | | 0±0 | | n/a | |  |
| 302 | gi\|119603743 | reelin, isoform CRA_b | | 5.36 | | 335068 | | 86 | | 3 | | 2.9 | | 0±0 | | no emPAI | | n/a | |  |
| 303 | gi\|119604405 | hCG21930, isoform CRA_c | | 5.09 | | 1451863 | | 271 | | 5 | | 3.8 | | 0±0 | | no emPAI | | n/a | |  |

**Supplementary Table 1.** Altered phosphoproteins in antibody-depedent enhancement (ADE) of DENV2-infected U937 cells (cont.)

| **No.** | **NCBI ID** | | **Protein** | | **pI** | | **MW (Da)** | | **Identification score** | | **No. of matched peptides** | | **%cov** | | **Mock (Mean±SD)** | | **Treated (Mean±SD)** | | **Ratio (Treated/Mock)** | |
| --- | --- | --- | --- | --- | --- | --- | --- | --- | --- | --- | --- | --- | --- | --- | --- | --- | --- | --- | --- | --- |
| 304 | gi\|119604470 | similar to Complement C3 precursor, isoform CRA_b | | 6.09 | | 180277 | | 96 | | 4 | | 5.1 | | 0±0 | | no emPAI | | n/a | |  |
| 305 | gi\|119604679 | zinc finger protein 443 | | 9.3 | | 77454 | | 77 | | 2 | | 11.5 | | no emPAI | | 0±0 | | n/a | |  |
| 306 | gi\|119604724 | microtubule associated serine/threonine kinase 1, isoform CRA_b | | 8.85 | | 172484 | | 120 | | 5 | | 6.9 | | 0±0 | | no emPAI | | n/a | |  |
| 307 | gi\|119605950 | CASK interacting protein 1, isoform CRA_b | | 9.54 | | 115406 | | 93 | | 4 | | 6.5 | | 0±0 | | no emPAI | | n/a | |  |
| 308 | gi\|119606211 | RAB11 family interacting protein 3 (class II), isoform CRA_a | | 4.25 | | 47464 | | 73 | | 2 | | 5.9 | | no emPAI | | 0±0 | | n/a | |  |
| 309 | gi\|119606213 | RAB11 family interacting protein 3 (class II), isoform CRA_c | | 4.77 | | 55175 | | 85 | | 3 | | 7.4 | | no emPAI | | 0±0 | | n/a | |  |
| 310 | gi\|119606839 | hCG2042943, partial | | 5.7 | | 287064 | | 120 | | 5 | | 3.2 | | 0±0 | | no emPAI | | n/a | |  |
| 311 | gi\|119607043 | hCG1644292 | | 6.44 | | 78091 | | 70 | | 2 | | 5.9 | | 0±0 | | no emPAI | | n/a | |  |
| 312 | gi\|119607157 | hCG2036697, isoform CRA_a, partial | | 7.3 | | 79949 | | 72 | | 2 | | 4.4 | | 0±0 | | no emPAI | | n/a | |  |
| 313 | gi\|119607236 | chromodomain helicase DNA binding protein 7, isoform CRA_a | | 6.02 | | 337359 | | 67 | | 2 | | 2.7 | | 0±0 | | no emPAI | | n/a | |  |
| 314 | gi\|119607256 | hCG1988300, isoform CRA_a | | 5.09 | | 47127 | | 73 | | 2 | | 10.3 | | no emPAI | | 0±0 | | n/a | |  |
| 315 | gi\|119607352 | chromosome 8 open reading frame 34, isoform CRA_b | | 9.24 | | 36234 | | 80 | | 3 | | 22.4 | | 0±0 | | no emPAI | | n/a | |  |
| 316 | gi\|119608372 | phosphatidic acid phosphatase type 2 domain containing 3, isoform CRA_a, partial | | 9.53 | | 35300 | | 72 | | 2 | | 9.5 | | 0±0 | | no emPAI | | n/a | |  |
| 317 | gi\|119608392 | Rap guanine nucleotide exchange factor (GEF) 1, isoform CRA_b | | 5.82 | | 123307 | | 73 | | 2 | | 4 | | 0±0 | | no emPAI | | n/a | |  |
| 318 | gi\|119608583 | potassium channel, subfamily T, member 1, isoform CRA_a | | 7.77 | | 141674 | | 77 | | 2 | | 2.6 | | no emPAI | | 0±0 | | n/a | |  |
| 319 | gi\|119608737 | ATP-binding cassette, sub-family A (ABC1), member 2, isoform CRA_a | | 6.3 | | 219430 | | 71 | | 2 | | 4.7 | | no emPAI | | 0±0 | | n/a | |  |

**Supplementary Table 1.** Altered phosphoproteins in antibody-depedent enhancement (ADE) of DENV2-infected U937 cells (cont.)

| **No.** | **NCBI ID** | | **Protein** | | **pI** | | **MW (Da)** | | **Identification score** | | **No. of matched peptides** | | **%cov** | | **Mock (Mean±SD)** | | **Treated (Mean±SD)** | | **Ratio (Treated/Mock)** | |
| --- | --- | --- | --- | --- | --- | --- | --- | --- | --- | --- | --- | --- | --- | --- | --- | --- | --- | --- | --- | --- |
| 320 | gi\|119608880 | hCG1979429, isoform CRA_a | | 9.32 | | 98055 | | 84 | | 3 | | 9.7 | | no emPAI | | 0±0 | | n/a | |  |
| 321 | gi\|119609248 | dual-specificity tyrosine-(Y)-phosphorylation regulated kinase 4, isoform CRA_b | | 9.45 | | 72149 | | 79 | | 2 | | 9.5 | | 0±0 | | no emPAI | | n/a | |  |
| 322 | gi\|119609306 | calcium channel, voltage-dependent, L type, alpha 1C subunit, isoform CRA_f | | 5.89 | | 238780 | | 115 | | 4 | | 3.8 | | 0±0 | | no emPAI | | n/a | |  |
| 323 | gi\|119610388 | dynein, axonemal, heavy polypeptide 9, isoform CRA_e | | 6.09 | | 255523 | | 70 | | 2 | | 3.2 | | no emPAI | | 0±0 | | n/a | |  |
| 324 | gi\|119610730 | NACHT, leucine rich repeat and PYD (pyrin domain) containing 1, isoform CRA_d, partial | | 6.5 | | 180965 | | 72 | | 2 | | 3.6 | | no emPAI | | 0±0 | | n/a | |  |
| 325 | gi\|119611190 | hCG1640003 | | 5.18 | | 120992 | | 66 | | 2 | | 4.6 | | no emPAI | | 0±0 | | n/a | |  |
| 326 | gi\|119611477 | centrosomal protein 350kDa, isoform CRA_b | | 5.51 | | 249124 | | 122 | | 5 | | 5.8 | | no emPAI | | 0±0 | | n/a | |  |
| 327 | gi\|119611839 | Jumonji, AT rich interactive domain 1B (RBP2-like), isoform CRA_b, partial | | 6.98 | | 189351 | | 72 | | 2 | | 3.8 | | 0±0 | | no emPAI | | n/a | |  |
| 328 | gi\|119612206 | sperm associated antigen 1, isoform CRA_a | | 6.24 | | 48001 | | 79 | | 2 | | 9.1 | | no emPAI | | 0±0 | | n/a | |  |
| 329 | gi\|119612425 | WD repeat domain 67, isoform CRA_b, partial | | 9.04 | | 117404 | | 71 | | 2 | | 5.2 | | no emPAI | | 0±0 | | n/a | |  |
| 330 | gi\|119612562 | thyroglobulin, isoform CRA_a, partial | | 5.66 | | 124171 | | 67 | | 2 | | 5.5 | | 0±0 | | no emPAI | | n/a | |  |
| 331 | gi\|119612929 | spectrin, beta, non-erythrocytic 5 | | 6.23 | | 416566 | | 121 | | 5 | | 3.2 | | 0±0 | | no emPAI | | n/a | |  |
| 332 | gi\|119613012 | microtubule-associated protein 1A, isoform CRA_a | | 4.86 | | 329580 | | 110 | | 4 | | 2.9 | | no emPAI | | 0±0 | | n/a | |  |
| 333 | gi\|119613102 | hCG2026745, isoform CRA_a | | 11.8 | | 34089 | | 87 | | 3 | | 19.3 | | no emPAI | | 0±0 | | n/a | |  |
| 334 | gi\|119613287 | hCG1643124 | | 5.98 | | 145171 | | 84 | | 3 | | 2.6 | | no emPAI | | 0±0 | | n/a | |  |
| 335 | gi\|119613524 | hCG1745555, isoform CRA_b | | 5.89 | | 353399 | | 76 | | 2 | | 2.8 | | 0±0 | | no emPAI | | n/a | |  |
| 336 | gi\|119613631 | hCG201263, isoform CRA_c, partial | | 8.98 | | 81934 | | 68 | | 2 | | 5 | | 0±0 | | no emPAI | | n/a | |  |

**Supplementary Table 1.** Altered phosphoproteins in antibody-depedent enhancement (ADE) of DENV2-infected U937 cells (cont.)

| **No.** | **NCBI ID** | | **Protein** | | **pI** | | **MW (Da)** | | **Identification score** | | **No. of matched peptides** | | **%cov** | | **Mock (Mean±SD)** | | **Treated (Mean±SD)** | | **Ratio (Treated/Mock)** | |
| --- | --- | --- | --- | --- | --- | --- | --- | --- | --- | --- | --- | --- | --- | --- | --- | --- | --- | --- | --- | --- |
| 337 | gi\|119614762 | ubiquitin specific peptidase 32, isoform CRA_a | | 6.38 | | 110840 | | 99 | | 4 | | 6.7 | | no emPAI | | 0±0 | | n/a | |  |
| 338 | gi\|119615270 | zinc finger, UBR1 type 1, isoform CRA_a | | 7.4 | | 261495 | | 102 | | 4 | | 4.4 | | no emPAI | | 0±0 | | n/a | |  |
| 339 | gi\|119615773 | BTG3 associated nuclear protein, isoform CRA_d | | 6.86 | | 39565 | | 67 | | 2 | | 7.7 | | 0±0 | | no emPAI | | n/a | |  |
| 340 | gi\|119616245 | cardiomyopathy associated 5 | | 4.83 | | 292454 | | 122 | | 5 | | 3.6 | | 0±0 | | no emPAI | | n/a | |  |
| 341 | gi\|119616471 | calpastatin, isoform CRA_b | | 5.01 | | 78823 | | 83 | | 3 | | 10 | | 0±0 | | no emPAI | | n/a | |  |
| 342 | gi\|119617203 | hCG21937, isoform CRA_b | | 5.93 | | 61821 | | 77 | | 2 | | 10.9 | | 0±0 | | no emPAI | | n/a | |  |
| 343 | gi\|119617245 | hCG2016179, isoform CRA_f | | 7.31 | | 99837 | | 66 | | 2 | | 4.9 | | 0±0 | | no emPAI | | n/a | |  |
| 344 | gi\|119617397 | low density lipoprotein-related protein 1 (alpha-2-macroglobulin receptor), isoform CRA_b | | 5.16 | | 497696 | | 163 | | 8 | | 5.1 | | no emPAI | | 0±0 | | n/a | |  |
| 345 | gi\|119618674 | F-box and leucine-rich repeat protein 10, isoform CRA_c, partial | | 9.19 | | 104618 | | 71 | | 2 | | 8.1 | | 0±0 | | no emPAI | | n/a | |  |
| 346 | gi\|119619136 | hCG1790904, isoform CRA_b, partial | | 9.67 | | 37271 | | 69 | | 2 | | 12 | | no emPAI | | 0±0 | | n/a | |  |
| 347 | gi\|119620540 | hCG1784313, isoform CRA_c | | 7.04 | | 214993 | | 96 | | 4 | | 6.3 | | no emPAI | | 0±0 | | n/a | |  |
| 348 | gi\|119620841 | latent transforming growth factor beta binding protein 1, isoform CRA_b | | 5.35 | | 174065 | | 78 | | 3 | | 4.8 | | 0±0 | | no emPAI | | n/a | |  |
| 349 | gi\|119620843 | latent transforming growth factor beta binding protein 1, isoform CRA_d | | 5.99 | | 107531 | | 73 | | 2 | | 6.3 | | 0±0 | | no emPAI | | n/a | |  |
| 350 | gi\|119620848 | baculoviral IAP repeat-containing 6 (apollon), isoform CRA_a | | 5.67 | | 527047 | | 127 | | 5 | | 2.2 | | 0±0 | | no emPAI | | n/a | |  |
| 351 | gi\|119621306 | neuroblastoma-amplified protein, isoform CRA_b | | 5.6 | | 264631 | | 90 | | 3 | | 4.9 | | no emPAI | | 0±0 | | n/a | |  |
| 352 | gi\|119621600 | hCG1646477, partial | | 8.63 | | 53091 | | 71 | | 2 | | 9 | | no emPAI | | 0±0 | | n/a | |  |
| 353 | gi\|119621962 | hCG1647671, isoform CRA_b | | 6.47 | | 106478 | | 68 | | 2 | | 5.6 | | 0±0 | | no emPAI | | n/a | |  |

**Supplementary Table 1.** Altered phosphoproteins in antibody-depedent enhancement (ADE) of DENV2-infected U937 cells (cont.)

| **No.** | **NCBI ID** | | **Protein** | | **pI** | | **MW (Da)** | | **Identification score** | | **No. of matched peptides** | | **%cov** | | **Mock (Mean±SD)** | | **Treated (Mean±SD)** | | **Ratio (Treated/Mock)** | |
| --- | --- | --- | --- | --- | --- | --- | --- | --- | --- | --- | --- | --- | --- | --- | --- | --- | --- | --- | --- | --- |
| 354 | gi\|119622364 | zinc finger protein 592, isoform CRA_c | | 8.16 | | 137997 | | 88 | | 3 | | 7.3 | | no emPAI | | 0±0 | | n/a | |  |
| 355 | gi\|119623948 | euchromatic histone-lysine N-methyltransferase 2, isoform CRA_d | | 5.42 | | 127284 | | 67 | | 2 | | 4.8 | | 0±0 | | no emPAI | | n/a | |  |
| 356 | gi\|119624234 | hCG17664, partial | | 8.69 | | 29849 | | 66 | | 2 | | 22.4 | | no emPAI | | 0±0 | | n/a | |  |
| 357 | gi\|119624415 | chromosome 6 open reading frame 130, isoform CRA_a | | 7.68 | | 9660 | | 73 | | 2 | | 22.1 | | 0±0 | | no emPAI | | n/a | |  |
| 358 | gi\|119625009 | hCG2025689 | | 8.94 | | 27365 | | 78 | | 3 | | 13.9 | | 0±0 | | no emPAI | | n/a | |  |
| 359 | gi\|119625101 | hCG17055 | | 9.52 | | 20576 | | 71 | | 2 | | 25 | | no emPAI | | 0±0 | | n/a | |  |
| 360 | gi\|119625271 | glutamate receptor, ionotropic, AMPA 2, isoform CRA_b | | 8 | | 103043 | | 111 | | 4 | | 11.2 | | no emPAI | | 0±0 | | n/a | |  |
| 361 | gi\|119625349 | dachsous 2 (Drosophila), isoform CRA_c | | 4.63 | | 305792 | | 87 | | 3 | | 1.9 | | no emPAI | | 0±0 | | n/a | |  |
| 362 | gi\|119625579 | PHD finger protein 17, isoform CRA_e | | 5.44 | | 56802 | | 67 | | 2 | | 9.9 | | no emPAI | | 0±0 | | n/a | |  |
| 363 | gi\|119625783 | oligophrenin 1 | | 7.87 | | 90100 | | 65 | | 2 | | 6.3 | | no emPAI | | 0±0 | | n/a | |  |
| 364 | gi\|119625810 | zinc finger CCCH-type containing 12B, isoform CRA_a, partial | | 7.18 | | 95624 | | 107 | | 4 | | 5.9 | | no emPAI | | 0±0 | | n/a | |  |
| 365 | gi\|119626372 | protein tyrosine phosphatase, non-receptor type 13 (APO-1/CD95 (Fas)-associated phosphatase), isoform CRA_c | | 5.88 | | 255765 | | 90 | | 3 | | 5 | | no emPAI | | 0±0 | | n/a | |  |
| 366 | gi\|119626424 | hect domain and RLD 6, isoform CRA_b | | 8.3 | | 116041 | | 65 | | 2 | | 3.8 | | 0±0 | | no emPAI | | n/a | |  |
| 367 | gi\|119626574 | centromere protein E, 312kDa, isoform CRA_a | | 5.47 | | 311956 | | 100 | | 4 | | 2.6 | | 0±0 | | no emPAI | | n/a | |  |
| 368 | gi\|119626575 | centromere protein E, 312kDa, isoform CRA_b, partial | | 5.44 | | 311794 | | 110 | | 4 | | 4.8 | | no emPAI | | 0±0 | | n/a | |  |
| 369 | gi\|119626610 | hCG2036814, isoform CRA_a | | 8.88 | | 58095 | | 66 | | 2 | | 7.2 | | 0±0 | | no emPAI | | n/a | |  |
| 370 | gi\|119626680 | alpha-kinase 1, isoform CRA_b | | 5.85 | | 138917 | | 86 | | 3 | | 5.9 | | 0±0 | | no emPAI | | n/a | |  |
| 371 | gi\|119626878 | DEP domain containing 1, isoform CRA_c | | 8.71 | | 83760 | | 72 | | 2 | | 6.6 | | 0±0 | | no emPAI | | n/a | |  |

**Supplementary Table 1.** Altered phosphoproteins in antibody-depedent enhancement (ADE) of DENV2-infected U937 cells (cont.)

| **No.** | **NCBI ID** | | **Protein** | | **pI** | | **MW (Da)** | | **Identification score** | | **No. of matched peptides** | | **%cov** | | **Mock (Mean±SD)** | | **Treated (Mean±SD)** | | **Ratio (Treated/Mock)** | |
| --- | --- | --- | --- | --- | --- | --- | --- | --- | --- | --- | --- | --- | --- | --- | --- | --- | --- | --- | --- | --- |
| 372 | gi\|119626879 | DEP domain containing 1, isoform CRA_d | | 8.75 | | 66910 | | 70 | | 2 | | 6.6 | | 0±0 | | no emPAI | | n/a | |  |
| 373 | gi\|119626973 | KIAA1799 protein, isoform CRA_a | | 9.29 | | 37261 | | 76 | | 2 | | 11.1 | | no emPAI | | 0±0 | | n/a | |  |
| 374 | gi\|119626989 | hCG24180 | | 6.75 | | 148014 | | 71 | | 2 | | 5.3 | | 0±0 | | no emPAI | | n/a | |  |
| 375 | gi\|119627353 | microtubule associated serine/threonine kinase 2, isoform CRA_a | | 8.41 | | 196242 | | 67 | | 2 | | 2.6 | | no emPAI | | 0±0 | | n/a | |  |
| 376 | gi\|119627682 | microtubule-actin crosslinking factor 1, isoform CRA_d | | 5.16 | | 452806 | | 170 | | 8 | | 4.3 | | no emPAI | | 0±0 | | n/a | |  |
| 377 | gi\|119628421 | myosin X, isoform CRA_a | | 5.79 | | 201722 | | 69 | | 2 | | 4 | | 0±0 | | no emPAI | | n/a | |  |
| 378 | gi\|119628893 | hCG2020187 | | 10.75 | | 8289 | | 74 | | 2 | | 27.5 | | no emPAI | | 0±0 | | n/a | |  |
| 379 | gi\|119629082 | A kinase (PRKA) anchor protein 11, isoform CRA_b | | 5.68 | | 162928 | | 111 | | 4 | | 5.4 | | 0±0 | | no emPAI | | n/a | |  |
| 380 | gi\|119629403 | dedicator of cytokinesis 9, isoform CRA_e | | 8.25 | | 210673 | | 116 | | 4 | | 5.8 | | no emPAI | | 0±0 | | n/a | |  |
| 381 | gi\|119629404 | dedicator of cytokinesis 9, isoform CRA_f | | 8.18 | | 238521 | | 84 | | 3 | | 6.1 | | 0±0 | | no emPAI | | n/a | |  |
| 382 | gi\|119629468 | hCG2011852 | | 9 | | 784513 | | 297 | | 5 | | 7.5 | | 0±0 | | no emPAI | | n/a | |  |
| 383 | gi\|119629561 | hCG2013041, isoform CRA_a | | 10.99 | | 38483 | | 66 | | 2 | | 21.4 | | no emPAI | | 0±0 | | n/a | |  |
| 384 | gi\|119631910 | nebulin, isoform CRA_g | | 9.1 | | 772492 | | 184 | | 8 | | 5.3 | | no emPAI | | 0±0 | | n/a | |  |
| 385 | gi\|119633228 | MORC family CW-type zinc finger protein 4 | | 8.35 | | 101885 | | 67 | | 2 | | 7.6 | | no emPAI | | 0±0 | | n/a | |  |
| 386 | gi\|11968023 | zinc finger protein 106 isoform 1 | | 6.73 | | 208754 | | 72 | | 2 | | 4.6 | | no emPAI | | 0±0 | | n/a | |  |
| 387 | gi\|12052818 | hypothetical protein | | 8.13 | | 73348 | | 85 | | 3 | | 8.4 | | no emPAI | | 0±0 | | n/a | |  |
| 388 | gi\|12053097 | hypothetical protein | | 8.8 | | 71766 | | 67 | | 2 | | 8.1 | | 0±0 | | no emPAI | | n/a | |  |
| 389 | gi\|12053245 | hypothetical protein | | 5.57 | | 162989 | | 66 | | 2 | | 2.9 | | 0±0 | | no emPAI | | n/a | |  |

**Supplementary Table 1.** Altered phosphoproteins in antibody-depedent enhancement (ADE) of DENV2-infected U937 cells (cont.)

| **No.** | **NCBI ID** | | **Protein** | | **pI** | | **MW (Da)** | | **Identification score** | | **No. of matched peptides** | | **%cov** | | **Mock (Mean±SD)** | | **Treated (Mean±SD)** | | **Ratio (Treated/Mock)** | |
| --- | --- | --- | --- | --- | --- | --- | --- | --- | --- | --- | --- | --- | --- | --- | --- | --- | --- | --- | --- | --- |
| 390 | gi\|12053709 | a disintegrin-like and metalloprotease (reprolysin type) with thrombospondin type 1 motif, 12 | | 8.25 | | 177430 | | 72 | | 2 | | 3.3 | | no emPAI | | 0±0 | | n/a | |  |
| 391 | gi\|12053795 | retinoid-acid induced protein 1 | | 9.06 | | 191526 | | 74 | | 2 | | 2.7 | | 0±0 | | no emPAI | | n/a | |  |
| 392 | gi\|1205999 | YRRM, partial | | 11.73 | | 4474 | | 83 | | 3 | | 37.2 | | no emPAI | | 0±0 | | n/a | |  |
| 393 | gi\|12081909 | semaphorin Y | | 8.36 | | 99596 | | 67 | | 2 | | 3.2 | | no emPAI | | 0±0 | | n/a | |  |
| 394 | gi\|121114283 | serine/threonine-protein kinase PLK4 isoform 1 | | 8.79 | | 108903 | | 78 | | 3 | | 6.2 | | no emPAI | | 0±0 | | n/a | |  |
| 395 | gi\|1212917 | axonal transporter of synaptic vesicles | | 5.93 | | 190964 | | 90 | | 3 | | 3.7 | | no emPAI | | 0±0 | | n/a | |  |
| 396 | gi\|121583483 | 1-phosphatidylinositol 3-phosphate 5-kinase isoform 2 | | 6.24 | | 236986 | | 105 | | 4 | | 4.1 | | 0±0 | | no emPAI | | n/a | |  |
| 397 | gi\|122065170 | RecName: Full=Dedicator of cytokinesis protein 7 | | 6.34 | | 242407 | | 77 | | 2 | | 3.8 | | 0±0 | | no emPAI | | n/a | |  |
| 398 | gi\|122937195 | perilipin-4 | | 8.92 | | 134349 | | 67 | | 2 | | 9.9 | | 0±0 | | no emPAI | | n/a | |  |
| 399 | gi\|122937255 | calmodulin-regulated spectrin-associated protein 3 isoform 1 | | 8.74 | | 137793 | | 77 | | 2 | | 4.3 | | no emPAI | | 0±0 | | n/a | |  |
| 400 | gi\|122937396 | coiled-coil domain-containing protein 150 | | 6.58 | | 128681 | | 94 | | 4 | | 5.5 | | no emPAI | | 0±0 | | n/a | |  |
| 401 | gi\|124028529 | symplekin | | 5.82 | | 141059 | | 77 | | 2 | | 3.1 | | 0±0 | | no emPAI | | n/a | |  |
| 402 | gi\|124297123 | ADAMTS12 protein | | 8.44 | | 168370 | | 78 | | 3 | | 4 | | no emPAI | | 0±0 | | n/a | |  |
| 403 | gi\|12597635 | B-cell lymphoma/leukemia 11B isoform 2 | | 6.05 | | 88420 | | 75 | | 2 | | 6 | | 0±0 | | no emPAI | | n/a | |  |
| 404 | gi\|12655125 | KIF1B protein | | 6.59 | | 64933 | | 80 | | 3 | | 10.5 | | 0±0 | | no emPAI | | n/a | |  |
| 405 | gi\|12697196 | huntingtin interacting protein 1 | | 5.48 | | 231031 | | 82 | | 3 | | 3.6 | | no emPAI | | 0±0 | | n/a | |  |
| 406 | gi\|12698017 | KIAA1736 protein | | 6.74 | | 136143 | | 116 | | 4 | | 5.2 | | 0±0 | | no emPAI | | n/a | |  |
| 407 | gi\|12698061 | KIAA1758 protein | | 8.24 | | 180806 | | 83 | | 3 | | 4.6 | | 0±0 | | no emPAI | | n/a | |  |
| 408 | gi\|12963885 | prostate antigen PARIS-1 | | 6.05 | | 104100 | | 77 | | 2 | | 7.2 | | no emPAI | | 0±0 | | n/a | |  |

**Supplementary Table 1.** Altered phosphoproteins in antibody-depedent enhancement (ADE) of DENV2-infected U937 cells (cont.)

| **No.** | **NCBI ID** | | **Protein** | | **pI** | | **MW (Da)** | | **Identification score** | | **No. of matched peptides** | | **%cov** | | **Mock (Mean±SD)** | | **Treated (Mean±SD)** | | **Ratio (Treated/Mock)** | |
| --- | --- | --- | --- | --- | --- | --- | --- | --- | --- | --- | --- | --- | --- | --- | --- | --- | --- | --- | --- | --- |
| 409 | gi\|129886 | RecName: Full=Aggrecan core protein; AltName: Full=Cartilage-specific proteoglycan core protein; Short=CSPCP; AltName: Full=Chondroitin sulfate proteoglycan core protein 1; Short=Chondroitin sulfate proteoglycan 1; Contains: RecName: Full=Aggrecan c | | 4.1 | | 250040 | | 78 | | 3 | | 2.4 | | 0±0 | | no emPAI | | n/a | |  |
| 410 | gi\|1314244 | complement C4B precursor | | 7.39 | | 188230 | | 83 | | 3 | | 6.8 | | no emPAI | | 0±0 | | n/a | |  |
| 411 | gi\|13183793 | CECR2 protein | | 6.56 | | 164111 | | 80 | | 3 | | 3.6 | | 0±0 | | no emPAI | | n/a | |  |
| 412 | gi\|13236565 | centromere protein O isoform 1 | | 7.63 | | 33764 | | 68 | | 2 | | 14.3 | | no emPAI | | 0±0 | | n/a | |  |
| 413 | gi\|13276641 | hypothetical protein | | 7.01 | | 61748 | | 74 | | 2 | | 9.6 | | no emPAI | | 0±0 | | n/a | |  |
| 414 | gi\|133777088 | SNRNP200 protein, partial | | 6.05 | | 216046 | | 100 | | 4 | | 4.1 | | 0±0 | | no emPAI | | n/a | |  |
| 415 | gi\|134104091 | Chain A, Crystal Structure Of Human Pyridoxal 5~-Phosphate Phosphatase | | 6.13 | | 31726 | | 92 | | 3 | | 12.1 | | 0±0 | | no emPAI | | n/a | |  |
| 416 | gi\|1353796 | Ig heavy chain variable region, partial | | 8.86 | | 16220 | | 70 | | 2 | | 39.6 | | 0±0 | | no emPAI | | n/a | |  |
| 417 | gi\|13560797 | ubiquitin specific protease | | 6.21 | | 143849 | | 102 | | 4 | | 5.5 | | no emPAI | | 0±0 | | n/a | |  |
| 418 | gi\|13603889 | ring finger protein 17 short isoform | | 5.83 | | 50445 | | 77 | | 2 | | 10 | | 0±0 | | no emPAI | | n/a | |  |
| 419 | gi\|13603893 | testis protein TEX15 | | 5.82 | | 315165 | | 171 | | 7 | | 6.1 | | no emPAI | | 0±0 | | n/a | |  |
| 420 | gi\|13606056 | DNA dependent protein kinase catalytic subunit | | 6.85 | | 465266 | | 100 | | 4 | | 2.9 | | no emPAI | | 0±0 | | n/a | |  |
| 421 | gi\|1374698 | nuclear protein, NP220 | | 6.01 | | 220492 | | 96 | | 4 | | 3.9 | | 0±0 | | no emPAI | | n/a | |  |
| 422 | gi\|13752754 | zinc finger 1111 | | 9.32 | | 95710 | | 77 | | 2 | | 5.5 | | no emPAI | | 0±0 | | n/a | |  |
| 423 | gi\|139394648 | DNA polymerase theta | | 7.07 | | 289437 | | 82 | | 3 | | 4.5 | | 0±0 | | no emPAI | | n/a | |  |
| 424 | gi\|14017821 | KIAA1802 protein | | 8.75 | | 90082 | | 76 | | 2 | | 7.2 | | 0±0 | | no emPAI | | n/a | |  |
| 425 | gi\|14017831 | KIAA1807 protein | | 8.25 | | 79517 | | 90 | | 3 | | 10.4 | | 0±0 | | no emPAI | | n/a | |  |

**Supplementary Table 1.** Altered phosphoproteins in antibody-depedent enhancement (ADE) of DENV2-infected U937 cells (cont.)

| **No.** | **NCBI ID** | **Protein** | | **pI** | | **MW (Da)** | | **Identification score** | | **No. of matched peptides** | | **%cov** | | **Mock (Mean±SD)** | | **Treated (Mean±SD)** | | **Ratio (Treated/Mock)** | |
| --- | --- | --- | --- | --- | --- | --- | --- | --- | --- | --- | --- | --- | --- | --- | --- | --- | --- | --- | --- |
| 426 | gi\|14017927 | KIAA1855 protein | 5.48 | | 137227 | | 137 | | 6 | | 9.1 | | 0±0 | | no emPAI | | n/a | |  |
| 427 | gi\|1405348 | zinc-finger DNA-binding protein | 6.41 | | 162103 | | 69 | | 2 | | 4.2 | | no emPAI | | 0±0 | | n/a | |  |
| 428 | gi\|14133213 | KIAA0732 protein | 7.54 | | 163012 | | 101 | | 4 | | 7.4 | | no emPAI | | 0±0 | | n/a | |  |
| 429 | gi\|14133235 | KIAA1093 protein | 6.6 | | 183156 | | 84 | | 3 | | 5.4 | | 0±0 | | no emPAI | | n/a | |  |
| 430 | gi\|14133239 | KIAA1121 protein | 5.64 | | 156557 | | 70 | | 2 | | 4.1 | | no emPAI | | 0±0 | | n/a | |  |
| 431 | gi\|14133241 | KIAA1124 protein | 6.11 | | 199084 | | 73 | | 2 | | 2 | | no emPAI | | 0±0 | | n/a | |  |
| 432 | gi\|14133247 | KIAA1250 protein | 6.22 | | 197087 | | 111 | | 4 | | 3.2 | | no emPAI | | 0±0 | | n/a | |  |
| 433 | gi\|14141161 | heterogeneous nuclear ribonucleoprotein U isoform b | 5.6 | | 88924 | | 184 | | 8 | | 12.9 | | no emPAI | | 0±0 | | n/a | |  |
| 434 | gi\|14149738 | neurolysin, mitochondrial | 6.21 | | 80600 | | 67 | | 2 | | 8.2 | | 0±0 | | no emPAI | | n/a | |  |
| 435 | gi\|14150134 | MICOS complex subunit MIC25 | 9.01 | | 26441 | | 72 | | 2 | | 15.7 | | 0±0 | | no emPAI | | n/a | |  |
| 436 | gi\|14165425 | protocadherin alpha-9 isoform 1 precursor | 4.94 | | 102339 | | 85 | | 3 | | 6.3 | | no emPAI | | 0±0 | | n/a | |  |
| 437 | gi\|14211816 | HBV pX associated protein 8 large isoform | 4.94 | | 162906 | | 107 | | 4 | | 7.5 | | no emPAI | | 0±0 | | n/a | |  |
| 438 | gi\|14249168 | proline/serine-rich coiled-coil protein 1 isoform a | 11.29 | | 35611 | | 65 | | 2 | | 14.7 | | 0±0 | | no emPAI | | n/a | |  |
| 439 | gi\|14249180 | protein Wnt-5b precursor | 8.8 | | 40297 | | 83 | | 3 | | 14.8 | | no emPAI | | 0±0 | | n/a | |  |
| 440 | gi\|14275558 | cyclin B3 | 6.2 | | 157800 | | 71 | | 2 | | 4 | | 0±0 | | no emPAI | | n/a | |  |
| 441 | gi\|14289323 | LIP isoform of BLIP | 5.84 | | 269282 | | 79 | | 2 | | 3.4 | | no emPAI | | 0±0 | | n/a | |  |
| 442 | gi\|1435038 | facioscapulohumeral muscular dystrophy | 11.87 | | 88973 | | 66 | | 2 | | 7.2 | | no emPAI | | 0±0 | | n/a | |  |
| 443 | gi\|145207314 | DPY19L2 protein, partial | 9.08 | | 80079 | | 94 | | 4 | | 7.1 | | no emPAI | | 0±0 | | n/a | |  |
| 444 | gi\|145558891 | RecName: Full=Arf-GAP with GTPase, ANK repeat and PH domain-containing protein 9; Short=AGAP-9; AltName: Full=Centaurin-gamma-like family member 6 | 8.15 | | 77922 | | 74 | | 2 | | 7.8 | | no emPAI | | 0±0 | | n/a | |  |

**Supplementary Table 1.** Altered phosphoproteins in antibody-depedent enhancement (ADE) of DENV2-infected U937 cells (cont.)

| **No.** | **NCBI ID** | **Protein** | | **pI** | | **MW (Da)** | | **Identification score** | | **No. of matched peptides** | | **%cov** | | **Mock (Mean±SD)** | | **Treated (Mean±SD)** | | **Ratio (Treated/Mock)** | |
| --- | --- | --- | --- | --- | --- | --- | --- | --- | --- | --- | --- | --- | --- | --- | --- | --- | --- | --- | --- |
| 445 | gi\|1469868 | KIAA0143 | 6.55 | | 98798 | | 87 | | 3 | | 4.6 | | no emPAI | | 0±0 | | n/a | |  |
| 446 | gi\|1477466 | vacuolar protein sorting homolog h-vps45 | 8.24 | | 64911 | | 72 | | 2 | | 11.2 | | 0±0 | | no emPAI | | n/a | |  |
| 447 | gi\|1477565 | p532 | 5.68 | | 531849 | | 66 | | 2 | | 1.7 | | no emPAI | | 0±0 | | n/a | |  |
| 448 | gi\|148664197 | coiled-coil domain-containing protein 40 isoform 1 | 5.21 | | 130033 | | 78 | | 3 | | 4.4 | | 0±0 | | no emPAI | | n/a | |  |
| 449 | gi\|1498229 | RNA helicase | 9.26 | | 68416 | | 82 | | 3 | | 11.8 | | 0±0 | | no emPAI | | n/a | |  |
| 450 | gi\|15026974 | obscurin | 5.54 | | 721240 | | 122 | | 4 | | 2.7 | | no emPAI | | 0±0 | | n/a | |  |
| 451 | gi\|15029372 | sorbin polypeptide | 8.03 | | 45945 | | 68 | | 2 | | 18.3 | | 0±0 | | no emPAI | | n/a | |  |
| 452 | gi\|1504026 | KIAA0223 | 5.77 | | 127266 | | 72 | | 2 | | 8.6 | | no emPAI | | 0±0 | | n/a | |  |
| 453 | gi\|1504030 | KIAA0225 | 5.85 | | 227903 | | 68 | | 2 | | 2.5 | | 0±0 | | no emPAI | | n/a | |  |
| 454 | gi\|15080775 | protein kinase NYD-SP5 | 9.19 | | 117302 | | 71 | | 2 | | 8.3 | | 0±0 | | no emPAI | | n/a | |  |
| 455 | gi\|15126774 | AHCTF1 protein, partial | 10.1 | | 25734 | | 84 | | 3 | | 18.3 | | no emPAI | | 0±0 | | n/a | |  |
| 456 | gi\|1518269 | TGN46 | 5.39 | | 45882 | | 65 | | 2 | | 12.1 | | 0±0 | | no emPAI | | n/a | |  |
| 457 | gi\|151946807 | ubiquitously transcribed tetratricopeptide repeat protein Y-linked transcript variant 95 | 7.85 | | 64274 | | 93 | | 4 | | 9.9 | | 0±0 | | no emPAI | | n/a | |  |
| 458 | gi\|151946813 | ubiquitously transcribed tetratricopeptide repeat protein Y-linked transcript variant 213 | 8.17 | | 129841 | | 66 | | 2 | | 3.7 | | no emPAI | | 0±0 | | n/a | |  |
| 459 | gi\|152060496 | RecName: Full=RNA-binding protein 44; AltName: Full=RNA-binding motif protein 44 | 5.48 | | 117911 | | 95 | | 4 | | 11.2 | | no emPAI | | 0±0 | | n/a | |  |
| 460 | gi\|15293789 | olfactory receptor | 8.93 | | 24584 | | 70 | | 2 | | 6.3 | | no emPAI | | 0±0 | | n/a | |  |
| 461 | gi\|1531548 | KIAA0083 | 8.25 | | 122343 | | 70 | | 2 | | 3.4 | | no emPAI | | 0±0 | | n/a | |  |
| 462 | gi\|15341763 | SWI/SNF related, matrix associated, actin dependent regulator of chromatin, subfamily c, member 2 | 5.4 | | 124798 | | 78 | | 3 | | 5.1 | | 0±0 | | no emPAI | | n/a | |  |

**Supplementary Table 1.** Altered phosphoproteins in antibody-depedent enhancement (ADE) of DENV2-infected U937 cells (cont.)

| **No.** | **NCBI ID** | **Protein** | | **pI** | | **MW (Da)** | | **Identification score** | | **No. of matched peptides** | | **%cov** | | **Mock (Mean±SD)** | | **Treated (Mean±SD)** | | **Ratio (Treated/Mock)** | |
| --- | --- | --- | --- | --- | --- | --- | --- | --- | --- | --- | --- | --- | --- | --- | --- | --- | --- | --- | --- |
| 463 | gi\|153791560 | zinc finger protein 799 | 9.29 | | 74240 | | 71 | | 2 | | 12 | | no emPAI | | 0±0 | | n/a | |  |
| 464 | gi\|153792259 | zinc finger protein 778 isoform 2 | 8.77 | | 81910 | | 75 | | 2 | | 10.6 | | no emPAI | | 0±0 | | n/a | |  |
| 465 | gi\|153945846 | inositol 1,4,5-trisphosphate receptor type 3 | 6.05 | | 303912 | | 117 | | 5 | | 4.7 | | 0±0 | | no emPAI | | n/a | |  |
| 466 | gi\|15395290 | axonemal beta heavy chain dynein type 11 | 6.03 | | 520649 | | 73 | | 2 | | 1.1 | | no emPAI | | 0±0 | | n/a | |  |
| 467 | gi\|154089856 | collagen type VI alpha 6 | 9.24 | | 100185 | | 85 | | 3 | | 8.3 | | 0±0 | | no emPAI | | n/a | |  |
| 468 | gi\|15421201 | SHAP-A | 8.46 | | 78250 | | 67 | | 2 | | 6.4 | | no emPAI | | 0±0 | | n/a | |  |
| 469 | gi\|1546900 | imogen 38 | 9.38 | | 45290 | | 79 | | 3 | | 17 | | 0±0 | | no emPAI | | n/a | |  |
| 470 | gi\|15529978 | zinc finger protein 622 | 5.8 | | 54237 | | 83 | | 3 | | 9.4 | | 0±0 | | no emPAI | | n/a | |  |
| 471 | gi\|155969693 | myomesin-3 | 5.89 | | 162088 | | 103 | | 4 | | 6.4 | | no emPAI | | 0±0 | | n/a | |  |
| 472 | gi\|1574998 | canalicular multispecific organic anion transporter | 8.53 | | 174170 | | 68 | | 2 | | 3 | | 0±0 | | no emPAI | | n/a | |  |
| 473 | gi\|157671929 | phosphatidylinositol 4-phosphate 3-kinase C2 domain-containing subunit alpha | 8.25 | | 190559 | | 65 | | 2 | | 3.3 | | 0±0 | | no emPAI | | n/a | |  |
| 474 | gi\|157830361 | Chain A, Human Serum Albumin In A Complex With Myristic Acid And Tri- Iodobenzoic Acid | 5.69 | | 65993 | | 80 | | 3 | | 13.3 | | 0±0 | | no emPAI | | n/a | |  |
| 475 | gi\|158420731 | chromodomain-helicase-DNA-binding protein 3 isoform 3 | 5.88 | | 232891 | | 79 | | 3 | | 3.4 | | 0±0 | | no emPAI | | n/a | |  |
| 476 | gi\|158513802 | RecName: Full=Putative protein FAM90A10P | 9.76 | | 49604 | | 73 | | 2 | | 5.2 | | 0±0 | | no emPAI | | n/a | |  |
| 477 | gi\|1585676 | acyl-CoA/cholesterol acyltransferase | 9.19 | | 64805 | | 65 | | 2 | | 3.5 | | no emPAI | | 0±0 | | n/a | |  |
| 478 | gi\|158939816 | RecName: Full=Uncharacterized protein CSNK1G2-AS1; AltName: Full=CSNK1G2 antisense RNA 1; AltName: Full=CSNK1G2 antisense gene protein 1 | 11.82 | | 16060 | | 66 | | 2 | | 17.6 | | 0±0 | | no emPAI | | n/a | |  |

**Supplementary Table 1.** Altered phosphoproteins in antibody-depedent enhancement (ADE) of DENV2-infected U937 cells (cont.)

| **No.** | **NCBI ID** | | **Protein** | | **pI** | | **MW (Da)** | | **Identification score** | | **No. of matched peptides** | | **%cov** | | **Mock (Mean±SD)** | | **Treated (Mean±SD)** | | **Ratio (Treated/Mock)** | |
| --- | --- | --- | --- | --- | --- | --- | --- | --- | --- | --- | --- | --- | --- | --- | --- | --- | --- | --- | --- | --- |
| 479 | gi\|159163976 | Chain A, Solution Structure Of The 14th Filamin Domain From Human Filamin C | | 5.17 | | 11731 | | 70 | | 2 | | 21.7 | | 0±0 | | no emPAI | | n/a | |  |
| 480 | gi\|160948599 | integrator complex subunit 1 | | 5.77 | | 244143 | | 80 | | 3 | | 2.4 | | no emPAI | | 0±0 | | n/a | |  |
| 481 | gi\|161761037 | Chain A, Crystal Structure Of Human Kynurenine Aminotransferase Ii | | 6.27 | | 47304 | | 66 | | 2 | | 7.3 | | 0±0 | | no emPAI | | n/a | |  |
| 482 | gi\|161761238 | Chain A, Crystal Structure Of Human Sulfotransferase Sult1c1 In Complex With Pap | | 7.26 | | 34783 | | 71 | | 2 | | 14.2 | | 0±0 | | no emPAI | | n/a | |  |
| 483 | gi\|16215696 | Sak | | 8.79 | | 109016 | | 103 | | 4 | | 9.9 | | 0±0 | | no emPAI | | n/a | |  |
| 484 | gi\|1628415 | vasopressin activated calcium mobilizing receptor-like protein | | 8.08 | | 90886 | | 81 | | 3 | | 7.2 | | 0±0 | | no emPAI | | n/a | |  |
| 485 | gi\|1666075 | ubiquitin hydrolase | | 5.51 | | 289438 | | 88 | | 3 | | 3.4 | | no emPAI | | 0±0 | | n/a | |  |
| 486 | gi\|168176987 | Chain A, Solution Structure Of The Human Abl2 Sh2 Domain | | 8.11 | | 12773 | | 68 | | 2 | | 19.3 | | no emPAI | | 0±0 | | n/a | |  |
| 487 | gi\|1685034 | beige protein homolog | | 6.15 | | 428852 | | 116 | | 4 | | 3 | | 0±0 | | no emPAI | | n/a | |  |
| 488 | gi\|168983559 | FYVE domain containing 26 zinc finger variant protein | | 5.98 | | 285736 | | 70 | | 2 | | 2.2 | | 0±0 | | no emPAI | | n/a | |  |
| 489 | gi\|16933557 | protocadherin-16 precursor | | 4.79 | | 345971 | | 89 | | 3 | | 1.9 | | no emPAI | | 0±0 | | n/a | |  |
| 490 | gi\|170016091 | teneurin-2 | | 6.23 | | 306691 | | 105 | | 4 | | 2.2 | | no emPAI | | 0±0 | | n/a | |  |
| 491 | gi\|17066105 | Titin | | 6.01 | | 3813840 | | 318 | | 12 | | 3.1 | | 0±0 | | no emPAI | | n/a | |  |
| 492 | gi\|172045554 | RecName: Full=Uncharacterized protein KIAA1107 | | 5.83 | | 155586 | | 103 | | 4 | | 7 | | no emPAI | | 0±0 | | n/a | |  |
| 493 | gi\|17225572 | KIAA1008 protein | | 6.69 | | 108907 | | 66 | | 2 | | 3.8 | | 0±0 | | no emPAI | | n/a | |  |
| 494 | gi\|17225574 | LIM domain only 7 | | 7.9 | | 153589 | | 83 | | 3 | | 5.4 | | 0±0 | | no emPAI | | n/a | |  |
| 495 | gi\|17505907 | probable ATP-dependent RNA helicase DDX31 isoform 1 | | 10.01 | | 94029 | | 72 | | 2 | | 4.7 | | 0±0 | | no emPAI | | n/a | |  |
| 496 | gi\|178872 | androgen receptor | | 6.07 | | 98825 | | 120 | | 5 | | 8.3 | | no emPAI | | 0±0 | | n/a | |  |

**Supplementary Table 1.** Altered phosphoproteins in antibody-depedent enhancement (ADE) of DENV2-infected U937 cells (cont.)

| **No.** | **NCBI ID** | | **Protein** | | **pI** | | **MW (Da)** | | **Identification score** | | **No. of matched peptides** | | **%cov** | | **Mock (Mean±SD)** | | **Treated (Mean±SD)** | | **Ratio (Treated/Mock)** | |
| --- | --- | --- | --- | --- | --- | --- | --- | --- | --- | --- | --- | --- | --- | --- | --- | --- | --- | --- | --- | --- |
| 497 | gi\|18079216 | caskin-1 | | 9.21 | | 149722 | | 90 | | 3 | | 6.4 | | 0±0 | | no emPAI | | n/a | |  |
| 498 | gi\|18105042 | GRB2-associated-binding protein 2 isoform a | | 8.54 | | 74411 | | 68 | | 2 | | 6.5 | | no emPAI | | 0±0 | | n/a | |  |
| 499 | gi\|181608 | desmoplakin, partial | | 6.68 | | 201237 | | 74 | | 2 | | 4.6 | | no emPAI | | 0±0 | | n/a | |  |
| 500 | gi\|181857 | dystrophin | | 5.64 | | 426411 | | 126 | | 5 | | 3.7 | | no emPAI | | 0±0 | | n/a | |  |
| 501 | gi\|1836081 | 76 kda membrane-linked metalloproteinase [human, myeloma cells, Peptide, 660 aa] | | 5.71 | | 72711 | | 85 | | 3 | | 12.1 | | 0±0 | | no emPAI | | n/a | |  |
| 502 | gi\|1840467 | bacterial MutS homolog | | 6.43 | | 152761 | | 126 | | 5 | | 7.1 | | 0±0 | | no emPAI | | n/a | |  |
| 503 | gi\|184394 | HRX | | 9.23 | | 431677 | | 135 | | 6 | | 4.4 | | 0±0 | | no emPAI | | n/a | |  |
| 504 | gi\|184569 | interferon-gamma induced protein | | 9.4 | | 82063 | | 75 | | 2 | | 7.4 | | no emPAI | | 0±0 | | n/a | |  |
| 505 | gi\|1846005 | collagen type XII alpha-1 | | 5.37 | | 332987 | | 83 | | 3 | | 3.4 | | 0±0 | | no emPAI | | n/a | |  |
| 506 | gi\|18490136 | ACOXL protein, partial | | 9.36 | | 36689 | | 82 | | 3 | | 12.9 | | no emPAI | | 0±0 | | n/a | |  |
| 507 | gi\|18676440 | FLJ00107 protein | | 6.84 | | 139057 | | 105 | | 4 | | 7.8 | | no emPAI | | 0±0 | | n/a | |  |
| 508 | gi\|18676552 | FLJ00173 protein | | 9.13 | | 139877 | | 82 | | 3 | | 4.7 | | no emPAI | | 0±0 | | n/a | |  |
| 509 | gi\|18676694 | FLJ00246 protein | | 4.96 | | 157713 | | 65 | | 2 | | 4.6 | | 0±0 | | no emPAI | | n/a | |  |
| 510 | gi\|189028874 | RecName: Full=Leucine-rich repeat-containing protein 9 | | 7.66 | | 166805 | | 67 | | 2 | | 3.6 | | 0±0 | | no emPAI | | n/a | |  |
| 511 | gi\|18916806 | KIAA1962 protein | | 8.91 | | 85259 | | 68 | | 2 | | 5.9 | | 0±0 | | no emPAI | | n/a | |  |
| 512 | gi\|189209 | nidogen | | 5.16 | | 136403 | | 77 | | 2 | | 3.6 | | no emPAI | | 0±0 | | n/a | |  |
| 513 | gi\|190165 | polyposis locus-encoded protein | | 7.75 | | 311466 | | 144 | | 6 | | 7.3 | | no emPAI | | 0±0 | | n/a | |  |
| 514 | gi\|190359882 | RecName: Full=Membrane-associated guanylate kinase, WW and PDZ domain-containing protein 3; AltName: Full=Membrane-associated guanylate kinase inverted 3; Short=MAGI-3 | | 8.17 | | 165506 | | 106 | | 4 | | 9.2 | | no emPAI | | 0±0 | | n/a | |  |

**Supplementary Table 1.** Altered phosphoproteins in antibody-depedent enhancement (ADE) of DENV2-infected U937 cells (cont.)

| **No.** | **NCBI ID** | | **Protein** | | **pI** | | **MW (Da)** | | **Identification score** | | **No. of matched peptides** | | **%cov** | | **Mock (Mean±SD)** | | **Treated (Mean±SD)** | | **Ratio (Treated/Mock)** | |
| --- | --- | --- | --- | --- | --- | --- | --- | --- | --- | --- | --- | --- | --- | --- | --- | --- | --- | --- | --- | --- |
| 515 | gi\|190396 | profilaggrin, partial | | 10.42 | | 106392 | | 72 | | 2 | | 8 | | no emPAI | | 0±0 | | n/a | |  |
| 516 | gi\|19115954 | dynein heavy chain 5, axonemal | | 5.79 | | 528684 | | 105 | | 4 | | 3.3 | | 0±0 | | no emPAI | | n/a | |  |
| 517 | gi\|1926311 | myosin I beta | | 9.51 | | 117965 | | 79 | | 3 | | 5.3 | | 0±0 | | no emPAI | | n/a | |  |
| 518 | gi\|193806213 | RecName: Full=Putative metallothionein MT1DP | | 8.37 | | 4979 | | 86 | | 3 | | 81.6 | | no emPAI | | 0±0 | | n/a | |  |
| 519 | gi\|19421557 | chromodomain helicase DNA binding protein 5 | | 5.9 | | 304903 | | 65 | | 2 | | 2.4 | | 0±0 | | no emPAI | | n/a | |  |
| 520 | gi\|195972871 | 1-phosphatidylinositol 4,5-bisphosphate phosphodiesterase eta-1 isoform a | | 7.87 | | 189104 | | 66 | | 2 | | 4.7 | | no emPAI | | 0±0 | | n/a | |  |
| 521 | gi\|197245440 | uncharacterized protein KIAA1107 | | 5.86 | | 149353 | | 135 | | 6 | | 8.9 | | 0±0 | | no emPAI | | n/a | |  |
| 522 | gi\|197313748 | histone-lysine N-methyltransferase SETD2 | | 5.8 | | 287418 | | 78 | | 3 | | 3.1 | | no emPAI | | 0±0 | | n/a | |  |
| 523 | gi\|19880521 | DOC-2/DAB2 interactive protein | | 6.35 | | 106685 | | 81 | | 3 | | 4.1 | | no emPAI | | 0±0 | | n/a | |  |
| 524 | gi\|19923084 | polycystic kidney disease protein 1-like 1 | | 6.64 | | 315233 | | 66 | | 2 | | 2.8 | | 0±0 | | no emPAI | | n/a | |  |
| 525 | gi\|203098334 | MYCBP-associated protein | | 8.35 | | 111856 | | 78 | | 3 | | 6.6 | | no emPAI | | 0±0 | | n/a | |  |
| 526 | gi\|203282279 | Chain A, Structure Of Human Haspin Kinase Domain | | 6.49 | | 38004 | | 93 | | 4 | | 8.6 | | no emPAI | | 0±0 | | n/a | |  |
| 527 | gi\|20379126 | small GTP binding protein TC10 | | 6.3 | | 23388 | | 90 | | 3 | | 16.4 | | 0±0 | | no emPAI | | n/a | |  |
| 528 | gi\|2052060 | WNT10B | | 9.79 | | 33239 | | 90 | | 3 | | 22.8 | | no emPAI | | 0±0 | | n/a | |  |
| 529 | gi\|20520995 | KIAA0311 | | 4.89 | | 257079 | | 93 | | 4 | | 3.1 | | 0±0 | | no emPAI | | n/a | |  |
| 530 | gi\|20521003 | KIAA0335 | | 9.38 | | 166789 | | 100 | | 4 | | 6.2 | | 0±0 | | no emPAI | | n/a | |  |
| 531 | gi\|20521690 | KIAA0904 protein | | 9.45 | | 168608 | | 69 | | 2 | | 3.7 | | 0±0 | | no emPAI | | n/a | |  |
| 532 | gi\|20521710 | KIAA0960 protein | | 7.32 | | 168095 | | 80 | | 3 | | 4.1 | | no emPAI | | 0±0 | | n/a | |  |
| 533 | gi\|20521718 | KIAA0979 protein | | 8.77 | | 168310 | | 76 | | 2 | | 4.5 | | no emPAI | | 0±0 | | n/a | |  |
| 534 | gi\|20521746 | KIAA1058 protein | | 7.26 | | 239265 | | 86 | | 3 | | 5.6 | | 0±0 | | no emPAI | | n/a | |  |
| 535 | gi\|20521750 | KIAA1064 protein | | 5.87 | | 141395 | | 88 | | 3 | | 5.8 | | 0±0 | | no emPAI | | n/a | |  |

**Supplementary Table 1.** Altered phosphoproteins in antibody-depedent enhancement (ADE) of DENV2-infected U937 cells (cont.)

| **No.** | **NCBI ID** | | **Protein** | | **pI** | | **MW (Da)** | | **Identification score** | | **No. of matched peptides** | | **%cov** | | **Mock (Mean±SD)** | | **Treated (Mean±SD)** | | **Ratio (Treated/Mock)** | |
| --- | --- | --- | --- | --- | --- | --- | --- | --- | --- | --- | --- | --- | --- | --- | --- | --- | --- | --- | --- | --- |
| 536 | gi\|20521756 | KIAA1078 protein | | 6.64 | | 154092 | | 108 | | 4 | | 8.9 | | 0±0 | | no emPAI | | n/a | |  |
| 537 | gi\|20521860 | KIAA0250 | | 9.18 | | 125772 | | 76 | | 2 | | 3.6 | | 0±0 | | no emPAI | | n/a | |  |
| 538 | gi\|207028821 | RNA-binding protein 44 | | 5.48 | | 118042 | | 85 | | 3 | | 9.7 | | 0±0 | | no emPAI | | n/a | |  |
| 539 | gi\|2072961 | putative p150 | | 9.68 | | 148984 | | 78 | | 3 | | 4.8 | | no emPAI | | 0±0 | | n/a | |  |
| 540 | gi\|2072977 | putative p150 | | 9.68 | | 148786 | | 71 | | 2 | | 3.7 | | no emPAI | | 0±0 | | n/a | |  |
| 541 | gi\|2078529 | Hlark | | 8.54 | | 40914 | | 68 | | 2 | | 13.7 | | no emPAI | | 0±0 | | n/a | |  |
| 542 | gi\|20799308 | voltage-gated potassium channel EAG2 | | 7.51 | | 111835 | | 77 | | 2 | | 4.1 | | no emPAI | | 0±0 | | n/a | |  |
| 543 | gi\|211939388 | Chain A, Crystal Structure Of Human Cgi121 | | 6.36 | | 19676 | | 79 | | 3 | | 15.3 | | 0±0 | | no emPAI | | n/a | |  |
| 544 | gi\|21245124 | adenosine deaminase domain-containing protein 1 isoform 1 | | 9.14 | | 64370 | | 83 | | 3 | | 9.4 | | 0±0 | | no emPAI | | n/a | |  |
| 545 | gi\|2125814 | serine/threonine protein kinase | | 8.84 | | 108887 | | 81 | | 3 | | 6.2 | | no emPAI | | 0±0 | | n/a | |  |
| 546 | gi\|2135479 | Ins P4-binding protein - human | | 7.92 | | 94581 | | 99 | | 4 | | 5.1 | | 0±0 | | no emPAI | | n/a | |  |
| 547 | gi\|21361659 | importin-9 | | 4.71 | | 115889 | | 70 | | 2 | | 3.8 | | 0±0 | | no emPAI | | n/a | |  |
| 548 | gi\|21361831 | partitioning defective 3 homolog isoform 1 | | 7.41 | | 151330 | | 91 | | 3 | | 4.1 | | no emPAI | | 0±0 | | n/a | |  |
| 549 | gi\|2143260 | phosphoinositide 3-kinase | | 8.24 | | 190617 | | 78 | | 3 | | 3.2 | | 0±0 | | no emPAI | | n/a | |  |
| 550 | gi\|21493037 | A-kinase anchor protein 4 isoform 1 | | 6.56 | | 94417 | | 89 | | 3 | | 8.9 | | 0±0 | | no emPAI | | n/a | |  |
| 551 | gi\|215261376 | Chain C, Crystal Structure Of The Pyruvate Dehydrogenase (E1p) Component Of Human Pyruvate Dehydrogenase Complex | | 6.94 | | 42488 | | 71 | | 2 | | 12.8 | | no emPAI | | 0±0 | | n/a | |  |
| 552 | gi\|21595423 | PHTF2 protein | | 6.41 | | 35285 | | 77 | | 2 | | 15.5 | | 0±0 | | no emPAI | | n/a | |  |
| 553 | gi\|21617878 | zinc finger 298 | | 8.56 | | 169092 | | 126 | | 5 | | 7.1 | | 0±0 | | no emPAI | | n/a | |  |
| 554 | gi\|21670711 | immunoglobulin heavy chain VHDJ region | | 8.6 | | 12941 | | 66 | | 2 | | 37.5 | | no emPAI | | 0±0 | | n/a | |  |

**Supplementary Table 1.** Altered phosphoproteins in antibody-depedent enhancement (ADE) of DENV2-infected U937 cells (cont.)

| **No.** | **NCBI ID** | | | **Protein** | | | **pI** | | | **MW (Da)** | | | **Identification score** | | | **No. of matched peptides** | | | **%cov** | | | **Mock (Mean±SD)** | | | **Treated (Mean±SD)** | | | **Ratio (Treated/Mock)** | | |
| --- | --- | --- | --- | --- | --- | --- | --- | --- | --- | --- | --- | --- | --- | --- | --- | --- | --- | --- | --- | --- | --- | --- | --- | --- | --- | --- | --- | --- | --- | --- |
| 555 | gi\|21706544 | KIAA1683 protein | | | 11.5 | | | 49162 | | | 69 | | | 2 | | | 7.7 | | | 0±0 | | | no emPAI | | | n/a | | |  |  |
| 556 | gi\|21724160 | | gastric cancer antigen Ga50 | | | 9.15 | | | 52755 | | | 71 | | | 2 | | | 8.3 | | | no emPAI | | | 0±0 | | | n/a | | |  |
| 557 | gi\|21740248 | | hypothetical protein | | | 6.5 | | | 118674 | | | 131 | | | 6 | | | 9.5 | | | 0±0 | | | no emPAI | | | n/a | | |  |
| 558 | gi\|21748558 | | FLJ00353 protein | | | 6.21 | | | 200216 | | | 69 | | | 2 | | | 2.5 | | | 0±0 | | | no emPAI | | | n/a | | |  |
| 559 | gi\|218047373 | | Eyes shut homolog | | | 5.52 | | | 350718 | | | 91 | | | 3 | | | 3.9 | | | no emPAI | | | 0±0 | | | n/a | | |  |
| 560 | gi\|2181869 | | DFFRY | | | 5.59 | | | 235613 | | | 90 | | | 3 | | | 3.9 | | | no emPAI | | | 0±0 | | | n/a | | |  |
| 561 | gi\|218505750 | | UPF0577 protein KIAA1324-like isoform 1 precursor | | | 5.76 | | | 113768 | | | 67 | | | 2 | | | 5.1 | | | 0±0 | | | no emPAI | | | n/a | | |  |
| 562 | gi\|21928632 | | seven transmembrane helix receptor | | | 8.44 | | | 99797 | | | 65 | | | 2 | | | 9.6 | | | no emPAI | | | 0±0 | | | n/a | | |  |
| 563 | gi\|219518067 | | SAFB protein | | | 5.3 | | | 102518 | | | 65 | | | 2 | | | 6.2 | | | 0±0 | | | no emPAI | | | n/a | | |  |
| 564 | gi\|219841908 | | COL22A1 protein | | | 7.01 | | | 129612 | | | 71 | | | 2 | | | 5.1 | | | no emPAI | | | 0±0 | | | n/a | | |  |
| 565 | gi\|219842343 | | metabotropic glutamate receptor 5 isoform a precursor | | | 8.07 | | | 132383 | | | 108 | | | 4 | | | 8.8 | | | 0±0 | | | no emPAI | | | n/a | | |  |
| 566 | gi\|221307607 | | ranBP2-like and GRIP domain-containing protein 3 | | | 5.95 | | | 197364 | | | 94 | | | 4 | | | 3 | | | 0±0 | | | no emPAI | | | n/a | | |  |
| 567 | gi\|22137761 | | Phospholipase C-like 2 | | | 5.95 | | | 113676 | | | 73 | | | 2 | | | 6.9 | | | no emPAI | | | 0±0 | | | n/a | | |  |
| 568 | gi\|222831647 | | serine-rich coiled-coil domain-containing protein 1 isoform 1 | | | 7.88 | | | 99449 | | | 80 | | | 3 | | | 9.1 | | | 0±0 | | | no emPAI | | | n/a | | |  |
| 569 | gi\|222875760 | | toll-like receptor 5 | | | 6.21 | | | 97576 | | | 82 | | | 3 | | | 5.7 | | | 0±0 | | | no emPAI | | | n/a | | |  |
| 570 | gi\|223459690 | | MGA protein | | | 6.39 | | | 314940 | | | 106 | | | 4 | | | 4 | | | no emPAI | | | 0±0 | | | n/a | | |  |
| 571 | gi\|22382105 | | Plexin A4 | | | 6.79 | | | 58069 | | | 78 | | | 3 | | | 8.8 | | | no emPAI | | | 0±0 | | | n/a | | |  |
| 572 | gi\|223890219 | | RNA-binding motif protein, X-linked-like-3 | | | 9.19 | | | 114868 | | | 105 | | | 4 | | | 13.4 | | | no emPAI | | | 0±0 | | | n/a | | |  |
| 573 | gi\|224451032 | | uncharacterized protein KIAA1683 isoform a | | | 10.2 | | | 147172 | | | 68 | | | 2 | | | 6.4 | | | 0±0 | | | no emPAI | | | n/a | | |  |
| 574 | gi\|2246558 | | talin-related protein | | | 4.74 | | | 20182 | | | 91 | | | 4 | | | 15.4 | | | no emPAI | | | 0±0 | | | n/a | | |  |

**Supplementary Table 1.** Altered phosphoproteins in antibody-depedent enhancement (ADE) of DENV2-infected U937 cells (cont.)

| **No.** | **NCBI ID** | | **Protein** | | **pI** | | **MW (Da)** | | **Identification score** | | **No. of matched peptides** | | **%cov** | | **Mock (Mean±SD)** | | **Treated (Mean±SD)** | | **Ratio (Treated/Mock)** | |
| --- | --- | --- | --- | --- | --- | --- | --- | --- | --- | --- | --- | --- | --- | --- | --- | --- | --- | --- | --- | --- |
| 575 | gi\|224808238 | B cell antibody heavy chain variable region, partial | | 9.3 | | 13564 | | 71 | | 2 | | 44.8 | | no emPAI | | 0±0 | | n/a | |  |
| 576 | gi\|22597106 | SWI/SNF chromatin remodeling complex subunit OSA2 | | 6.23 | | 229741 | | 66 | | 2 | | 3.2 | | no emPAI | | 0±0 | | n/a | |  |
| 577 | gi\|226246537 | zinc finger protein 407 isoform 3 | | 6.53 | | 183965 | | 87 | | 3 | | 5.4 | | no emPAI | | 0±0 | | n/a | |  |
| 578 | gi\|22671723 | hypothetical protein | | 8.22 | | 103856 | | 66 | | 2 | | 4.3 | | 0±0 | | no emPAI | | n/a | |  |
| 579 | gi\|22748931 | F-box/LRR-repeat protein 14 | | 9.15 | | 45857 | | 84 | | 3 | | 15.3 | | 0±0 | | no emPAI | | n/a | |  |
| 580 | gi\|22902196 | Thymine-DNA glycosylase | | 6.36 | | 45982 | | 73 | | 2 | | 10.5 | | 0±0 | | no emPAI | | n/a | |  |
| 581 | gi\|22902472 | Izumo sperm-egg fusion 1 | | 6.11 | | 38933 | | 65 | | 2 | | 9.4 | | 0±0 | | no emPAI | | n/a | |  |
| 582 | gi\|23097321 | zinc finger protein 383 | | 8.21 | | 54577 | | 90 | | 3 | | 10.5 | | 0±0 | | no emPAI | | n/a | |  |
| 583 | gi\|23097323 | zinc finger protein 92 isoform 2 | | 9.4 | | 68442 | | 70 | | 2 | | 10.4 | | no emPAI | | 0±0 | | n/a | |  |
| 584 | gi\|23272879 | SET and MYND domain containing 4 | | 6.33 | | 89093 | | 110 | | 4 | | 10 | | no emPAI | | 0±0 | | n/a | |  |
| 585 | gi\|23273923 | Coiled-coil domain containing 13 | | 8.93 | | 80804 | | 85 | | 3 | | 7 | | 0±0 | | no emPAI | | n/a | |  |
| 586 | gi\|2343289 | NMDAR1 subunit isoform 4b | | 8.98 | | 105981 | | 66 | | 2 | | 5.2 | | 0±0 | | no emPAI | | n/a | |  |
| 587 | gi\|23957299 | ATP-binding cassette transporter family A member 12 | | 7.52 | | 264796 | | 65 | | 2 | | 1.8 | | 0±0 | | no emPAI | | n/a | |  |
| 588 | gi\|239985422 | pyruvate dehyrogenase phosphatase catalytic subunit 1 isoform 2 | | 6.35 | | 63655 | | 66 | | 2 | | 8.5 | | no emPAI | | 0±0 | | n/a | |  |
| 589 | gi\|241913483 | Chain A, Crystal Structure Of Hedgehog-Interacting Protein (Hhip) | | 6.89 | | 53834 | | 84 | | 3 | | 14.1 | | no emPAI | | 0±0 | | n/a | |  |
| 590 | gi\|2429079 | Laminin-5 beta3 chain | | 7.31 | | 129492 | | 69 | | 2 | | 5.7 | | no emPAI | | 0±0 | | n/a | |  |
| 591 | gi\|24307913 | zinc finger protein 234 | | 8.96 | | 80507 | | 89 | | 3 | | 9.4 | | no emPAI | | 0±0 | | n/a | |  |
| 592 | gi\|2432000 | Munc13 | | 5.7 | | 180569 | | 156 | | 6 | | 6.2 | | 0±0 | | no emPAI | | n/a | |  |
| 593 | gi\|24431985 | piwi-like protein 2 | | 9.16 | | 109778 | | 69 | | 2 | | 3.9 | | no emPAI | | 0±0 | | n/a | |  |
| 594 | gi\|2454510 | PSD-95/SAP90-associated protein-2, partial | | 7.26 | | 105348 | | 74 | | 2 | | 10.5 | | 0±0 | | no emPAI | | n/a | |  |

**Supplementary Table 1.** Altered phosphoproteins in antibody-depedent enhancement (ADE) of DENV2-infected U937 cells (cont.)

| **No.** | **NCBI ID** | | **Protein** | | **pI** | | **MW (Da)** | | **Identification score** | | **No. of matched peptides** | | **%cov** | | **Mock (Mean±SD)** | | **Treated (Mean±SD)** | | **Ratio (Treated/Mock)** | |
| --- | --- | --- | --- | --- | --- | --- | --- | --- | --- | --- | --- | --- | --- | --- | --- | --- | --- | --- | --- | --- |
| 595 | gi\|246466 | hIRS-1 | | 8.91 | | 131694 | | 76 | | 2 | | 7.8 | | 0±0 | | no emPAI | | n/a | |  |
| 596 | gi\|2467369 | SCP-1 | | 5.84 | | 113740 | | 73 | | 2 | | 4.7 | | no emPAI | | 0±0 | | n/a | |  |
| 597 | gi\|247425769 | immunoglobulin heavy chain variable region | | 8.98 | | 13556 | | 71 | | 2 | | 8.1 | | 0±0 | | no emPAI | | n/a | |  |
| 598 | gi\|24899176 | KIAA2005 protein | | 8.19 | | 184765 | | 74 | | 2 | | 4.3 | | 0±0 | | no emPAI | | n/a | |  |
| 599 | gi\|24899180 | KIAA2007 protein | | 8.73 | | 65727 | | 77 | | 2 | | 7.2 | | 0±0 | | no emPAI | | n/a | |  |
| 600 | gi\|24899210 | KIAA2023 protein | | 8.73 | | 125860 | | 117 | | 5 | | 6.7 | | no emPAI | | 0±0 | | n/a | |  |
| 601 | gi\|25014054 | RecName: Full=Sodium channel protein type 3 subunit alpha; AltName: Full=Sodium channel protein brain III subunit alpha; AltName: Full=Sodium channel protein type III subunit alpha; AltName: Full=Voltage-gated sodium channel subtype III; AltName: Fu | | 5.6 | | 226147 | | 77 | | 2 | | 4.3 | | 0±0 | | no emPAI | | n/a | |  |
| 602 | gi\|253722490 | Chain P, Human Plasminogen Activator Inhibitor-2.[loop (66-98) Deletion Mutant] Complexed With Peptide N-acetyl-teaaagmggvmtgr-oh | | 3.99 | | 1419 | | 65 | | 2 | | 100 | | no emPAI | | 0±0 | | n/a | |  |
| 603 | gi\|253735775 | rho guanine nucleotide exchange factor 2 isoform 1 | | 6.89 | | 111473 | | 81 | | 3 | | 8.1 | | no emPAI | | 0±0 | | n/a | |  |
| 604 | gi\|25535933 | KIAA0411 protein [imported] - human | | 6.14 | | 121711 | | 83 | | 3 | | 7.3 | | 0±0 | | no emPAI | | n/a | |  |
| 605 | gi\|255733129 | metabotropic glutamate receptor 5 variant F | | 8.52 | | 112483 | | 77 | | 2 | | 7.6 | | 0±0 | | no emPAI | | n/a | |  |
| 606 | gi\|256574792 | putative ankyrin repeat domain-containing protein 31 | | 5.82 | | 210685 | | 90 | | 3 | | 3.5 | | no emPAI | | 0±0 | | n/a | |  |
| 607 | gi\|257480833 | LIM domain only 7 protein | | 6.86 | | 186126 | | 85 | | 3 | | 4.2 | | 0±0 | | no emPAI | | n/a | |  |
| 608 | gi\|25955554 | RASGRF1 protein | | 7.56 | | 133820 | | 67 | | 2 | | 5 | | no emPAI | | 0±0 | | n/a | |  |

**Supplementary Table 1.** The altered phosphoproteins in antibody-depedent enhancement (ADE) of DENV2-infected U937 cells (cont.)

| **No.** | **NCBI ID** | **Protein** | **pI** | **MW (Da)** | **Identification score** | **No. of matched peptides** | **%cov** | **Mock (Mean±SD)** | **Treated (Mean±SD)** | **Ratio (Treated/Mock)** |
| --- | --- | --- | --- | --- | --- | --- | --- | --- | --- | --- |
| 609 | gi\|26051231 | serine beta-lactamase-like protein LACTB, mitochondrial isoform a precursor | 8.71 | 60655 | 71 | 2 | 10.6 | 0±0 | no emPAI | n/a |
| 610 | gi\|261278699 | Chain A, Human Sod1 A4v Metal-Free Variant | 5.54 | 15934 | 72 | 2 | 22.7 | 0±0 | no emPAI | n/a |
| 611 | gi\|261862237 | disks large homolog 3 isoform c | 8.7 | 42085 | 70 | 2 | 4.9 | 0±0 | no emPAI | n/a |
| 612 | gi\|262359911 | AF4/FMR2 family member 1 isoform 1 | 9.29 | 132302 | 90 | 3 | 4.8 | no emPAI | 0±0 | n/a |
| 613 | gi\|26251774 | MAP7D3 protein, partial | 9.54 | 101602 | 77 | 2 | 8 | no emPAI | 0±0 | n/a |
| 614 | gi\|27369027 | immunoglobulin kappa light chain variable region | 9.01 | 12063 | 73 | 2 | 8 | no emPAI | 0±0 | n/a |
| 615 | gi\|27369048 | immunoglobulin lambda light chain variable region | 6.75 | 14540 | 70 | 2 | 18.6 | no emPAI | 0±0 | n/a |
| 616 | gi\|27371076 | Kelch-like 8 (Drosophila) | 6.18 | 68817 | 99 | 4 | 7.7 | 0±0 | no emPAI | n/a |
| 617 | gi\|2738149 | kinesin-like motor protein KIF1C | 6.32 | 122995 | 105 | 4 | 4.5 | no emPAI | 0±0 | n/a |
| 618 | gi\|27436920 | C-Jun-amino-terminal kinase-interacting protein 4 isoform 3 | 5.07 | 144592 | 66 | 2 | 3.2 | 0±0 | no emPAI | n/a |
| 619 | gi\|27451604 | UBR1 E3a ligase | 5.75 | 195154 | 111 | 4 | 4.6 | no emPAI | 0±0 | n/a |
| 620 | gi\|27469519 | DENN/MADD domain containing 4A | 7.07 | 209095 | 79 | 3 | 3.1 | 0±0 | no emPAI | n/a |
| 621 | gi\|27477095 | histone-lysine N-methyltransferase, H3 lysine-36 and H4 lysine-20 specific isoform a | 8.83 | 267171 | 131 | 6 | 3.5 | no emPAI | 0±0 | n/a |
| 622 | gi\|27526775 | steerin1 protein | 8.37 | 198434 | 155 | 7 | 6.3 | no emPAI | 0±0 | n/a |
| 623 | gi\|27597090 | transcription elongation factor SPT6 | 4.81 | 198949 | 66 | 2 | 3.7 | 0±0 | no emPAI | n/a |
| 624 | gi\|27805669 | RecName: Full=Pantothenate kinase 4; Short=hPanK4; AltName: Full=Pantothenic acid kinase 4 | 5.88 | 85937 | 97 | 4 | 10.9 | 0±0 | no emPAI | n/a |
| 625 | gi\|28173564 | keratin, type II cytoskeletal 73 | 6.93 | 58887 | 76 | 2 | 10.7 | 0±0 | no emPAI | n/a |

**Supplementary Table 1.** Altered phosphoproteins in antibody-depedent enhancement (ADE) of DENV2-infected U937 cells (cont.)

| **No.** | **NCBI ID** | **Protein** | **pI** | **MW (Da)** | **Identification score** | **No. of matched peptides** | **%cov** | **Mock (Mean±SD)** | **Treated (Mean±SD)** | **Ratio (Treated/Mock)** |
| --- | --- | --- | --- | --- | --- | --- | --- | --- | --- | --- |
| 626 | gi\|284052 | complement component C3b - human (fragments) | 4.49 | 25280 | 81 | 3 | 24.2 | no emPAI | 0±0 | n/a |
| 627 | gi\|288188943 | immunoglobulin epsilon variable region, partial | 9.22 | 12627 | 88 | 3 | 28.7 | no emPAI | 0±0 | n/a |
| 628 | gi\|28933422 | transforming acidic coiled coil 2, long isoform | 4.72 | 309213 | 123 | 6 | 4.2 | no emPAI | 0±0 | n/a |
| 629 | gi\|28933438 | fibrocystin L | 5.71 | 465453 | 68 | 2 | 1.8 | no emPAI | 0±0 | n/a |
| 630 | gi\|289547589 | 1-phosphatidylinositol 4,5-bisphosphate phosphodiesterase beta-4 isoform a | 6.37 | 136020 | 72 | 2 | 4.1 | 0±0 | no emPAI | n/a |
| 631 | gi\|29126954 | RTTN protein, partial | 6.08 | 210004 | 91 | 3 | 3.5 | 0±0 | no emPAI | n/a |
| 632 | gi\|291621660 | dmX-like protein 2 isoform 3 | 6.12 | 269977 | 81 | 3 | 2.4 | no emPAI | 0±0 | n/a |
| 633 | gi\|293597574 | transient receptor potential cation channel subfamily M member 6 isoform c | 7.86 | 230808 | 66 | 2 | 3.3 | 0±0 | no emPAI | n/a |
| 634 | gi\|29421196 | KIAA1476 protein | 6.14 | 237463 | 81 | 3 | 3.7 | no emPAI | 0±0 | n/a |
| 635 | gi\|296278200 | partitioning defective 3 homolog isoform 8 | 7.66 | 138247 | 79 | 3 | 4.6 | no emPAI | 0±0 | n/a |
| 636 | gi\|296453070 | RecName: Full=Zinc finger protein 229 | 8.88 | 93647 | 97 | 4 | 8.1 | no emPAI | 0±0 | n/a |
| 637 | gi\|29691197 | VEGFR3 long form | 5.84 | 152685 | 87 | 3 | 4.3 | 0±0 | no emPAI | n/a |
| 638 | gi\|297718104 | xeroderma pigmentosum complementation group C | 8.96 | 105817 | 114 | 4 | 7.9 | 0±0 | no emPAI | n/a |
| 639 | gi\|29789445 | desmoglein-4 isoform 2 preproprotein | 4.42 | 113751 | 68 | 2 | 6.7 | 0±0 | no emPAI | n/a |
| 640 | gi\|298111 | XP-G factor | 5.19 | 133247 | 68 | 2 | 3 | 0±0 | no emPAI | n/a |
| 641 | gi\|300193049 | protein FAM193B | 6.84 | 87493 | 66 | 2 | 4.3 | 0±0 | no emPAI | n/a |
| 642 | gi\|300508350 | Chain A, Crystal Structure Of The Kinase Domain Of Human Traf2- And Nck-Interacting Kinase With Wee1chk1 Inhibitor | 7.16 | 37160 | 75 | 2 | 13.8 | 0±0 | no emPAI | n/a |
| 643 | gi\|30089664 | ABC A13 | 5.99 | 575806 | 101 | 4 | 1.9 | 0±0 | no emPAI | n/a |

**Supplementary Table 1.** Altered phosphoproteins in antibody-depedent enhancement (ADE) of DENV2-infected U937 cells (cont.)

| **No.** | **NCBI ID** | **Protein** | **pI** | **MW (Da)** | **Identification score** | **No. of matched peptides** | **%cov** | **Mock (Mean±SD)** | **Treated (Mean±SD)** | **Ratio (Treated/Mock)** |
| --- | --- | --- | --- | --- | --- | --- | --- | --- | --- | --- |
| 644 | gi\|3021392 | nuclear protein SDK3 | 6.54 | 80908 | 70 | 2 | 8 | no emPAI | 0±0 | n/a |
| 645 | gi\|3043646 | KIAA0561 protein | 8.25 | 142918 | 77 | 2 | 4.3 | 0±0 | no emPAI | n/a |
| 646 | gi\|304562956 | immunoglobulin gamma 1 heavy chain variable region | 9.32 | 14619 | 70 | 2 | 35.8 | no emPAI | 0±0 | n/a |
| 647 | gi\|306991739 | Chain P, 14-3-3 Sigma In Complex With Yap Ps127-Peptide | 9.63 | 1076 | 80 | 3 | 100 | 0±0 | no emPAI | n/a |
| 648 | gi\|30722289 | hypothetical protein | 9.19 | 98291 | 100 | 4 | 11.7 | no emPAI | 0±0 | n/a |
| 649 | gi\|307506 | thrombospondin 2 | 4.63 | 129872 | 80 | 3 | 3.3 | 0±0 | no emPAI | n/a |
| 650 | gi\|3075509 | serum-inducible kinase | 8.52 | 78172 | 103 | 4 | 7.4 | 0±0 | no emPAI | n/a |
| 651 | gi\|30794488 | kinesin-like protein KIF27 isoform A | 6.9 | 160184 | 70 | 2 | 3.9 | no emPAI | 0±0 | n/a |
| 652 | gi\|308153472 | RecName: Full=PH-interacting protein; Short=PHIP; AltName: Full=IRS-1 PH domain-binding protein; AltName: Full=WD repeat-containing protein 11 | 9.02 | 206560 | 91 | 3 | 3.4 | 0±0 | no emPAI | n/a |
| 653 | gi\|308153585 | RecName: Full=LIM domain only protein 7; Short=LMO-7; AltName: Full=F-box only protein 20; AltName: Full=LOMP | 8.34 | 192576 | 76 | 2 | 5.5 | no emPAI | 0±0 | n/a |
| 654 | gi\|308387374 | cAMP-specific 3~,5~-cyclic phosphodiesterase 4D isoform PDE4D5 | 5.03 | 84375 | 71 | 2 | 5.4 | no emPAI | 0±0 | n/a |
| 655 | gi\|3098675 | cell cycle regulatory protein p95 | 6.66 | 84905 | 108 | 4 | 11.1 | 0±0 | no emPAI | n/a |
| 656 | gi\|310689690 | Chain A, Crystal Structure Of The Complex Of The Interacting Regions Of Dock2 And Elmo1 | 7.03 | 21027 | 78 | 2 | 7.1 | 0±0 | no emPAI | n/a |
| 657 | gi\|31071795 | CENTRIOLIN | 5.44 | 268734 | 74 | 2 | 4.1 | no emPAI | 0±0 | n/a |
| 658 | gi\|3126878 | M4 protein deletion mutant | 8.9 | 73512 | 119 | 5 | 17.9 | no emPAI | 0±0 | n/a |
| 659 | gi\|3135792 | FIM protein | 6.27 | 155053 | 76 | 2 | 5.4 | 0±0 | no emPAI | n/a |

**Supplementary Table 1.** Altered phosphoproteins in antibody-depedent enhancement (ADE) of DENV2-infected U937 cells (cont.)

| **No.** | **NCBI ID** | **Protein** | **pI** | **MW (Da)** | **Identification score** | **No. of matched peptides** | **%cov** | **Mock (Mean±SD)** | **Treated (Mean±SD)** | **Ratio (Treated/Mock)** |
| --- | --- | --- | --- | --- | --- | --- | --- | --- | --- | --- |
| 660 | gi\|31377568 | signal peptide, CUB and EGF-like domain-containing protein 3 isoform 1 precursor | 7.85 | 109210 | 75 | 2 | 8.4 | 0±0 | no emPAI | n/a |
| 661 | gi\|31718 | membrane protein | 5.51 | 151132 | 111 | 4 | 7.8 | no emPAI | 0±0 | n/a |
| 662 | gi\|31873280 | hypothetical protein | 5.3 | 115641 | 67 | 2 | 5.4 | 0±0 | no emPAI | n/a |
| 663 | gi\|31873308 | hypothetical protein | 8.51 | 122861 | 83 | 3 | 6.3 | no emPAI | 0±0 | n/a |
| 664 | gi\|31873332 | hypothetical protein | 5.72 | 190180 | 83 | 3 | 3.6 | 0±0 | no emPAI | n/a |
| 665 | gi\|31873759 | hypothetical protein | 8.06 | 102221 | 82 | 3 | 7.9 | 0±0 | no emPAI | n/a |
| 666 | gi\|31873949 | hypothetical protein | 9.49 | 65920 | 65 | 2 | 5.2 | 0±0 | no emPAI | n/a |
| 667 | gi\|31874109 | hypothetical protein | 5.75 | 268577 | 67 | 2 | 4.3 | no emPAI | 0±0 | n/a |
| 668 | gi\|31874561 | hypothetical protein | 4.98 | 108550 | 68 | 2 | 4.5 | no emPAI | 0±0 | n/a |
| 669 | gi\|3192971 | gastric mucin | 6.51 | 147690 | 77 | 2 | 3.7 | 0±0 | no emPAI | n/a |
| 670 | gi\|31958 | glutaminyl-tRNA synthetase | 7.77 | 162923 | 92 | 3 | 7.4 | no emPAI | 0±0 | n/a |
| 671 | gi\|31982906 | cingulin-like protein 1 | 5.51 | 148989 | 73 | 2 | 3.4 | 0±0 | no emPAI | n/a |
| 672 | gi\|323432777 | immunoglobulin variable region | 5.35 | 15582 | 82 | 3 | 13.8 | no emPAI | 0±0 | n/a |
| 673 | gi\|323462149 | methyl-CpG-binding domain protein 1 isoform 7 | 9.49 | 69572 | 90 | 3 | 7.1 | 0±0 | no emPAI | n/a |
| 674 | gi\|32400196 | hypothetical protein | 8.54 | 153764 | 77 | 2 | 6 | 0±0 | no emPAI | n/a |
| 675 | gi\|3242214 | DRIM protein | 7.07 | 318223 | 128 | 6 | 3.8 | no emPAI | 0±0 | n/a |
| 676 | gi\|3243075 | melastatin 1 | 6.4 | 174302 | 67 | 2 | 3.1 | 0±0 | no emPAI | n/a |
| 677 | gi\|326632820 | sialophosphoprotein variant A | 3.29 | 72789 | 66 | 2 | 5.8 | no emPAI | 0±0 | n/a |
| 678 | gi\|32699565 | RecName: Full=Unconventional myosin-XVIIIb | 6.49 | 285008 | 122 | 6 | 4.8 | 0±0 | no emPAI | n/a |
| 679 | gi\|3288457 | PI3-kinase | 6.54 | 165989 | 88 | 3 | 3.7 | no emPAI | 0±0 | n/a |
| 680 | gi\|33150576 | nuclear receptor co-repressor | 6.51 | 258722 | 85 | 3 | 4.1 | 0±0 | no emPAI | n/a |

**Supplementary Table 1.** Altered phosphoproteins in antibody-depedent enhancement (ADE) of DENV2-infected U937 cells (cont.)

| **No.** | **NCBI ID** | **Protein** | **pI** | **MW (Da)** | **Identification score** | **No. of matched peptides** | **%cov** | **Mock (Mean±SD)** | **Treated (Mean±SD)** | **Ratio (Treated/Mock)** |
| --- | --- | --- | --- | --- | --- | --- | --- | --- | --- | --- |
| 681 | gi\|33243958 | AT-hook transcription factor | 5.91 | 155054 | 78 | 2 | 4.9 | 0±0 | no emPAI | n/a |
| 682 | gi\|3327082 | KIAA0634 protein | 5.5 | 145332 | 75 | 2 | 6.1 | no emPAI | 0±0 | n/a |
| 683 | gi\|3327188 | KIAA0687 protein | 7.9 | 134588 | 113 | 4 | 4.7 | DIV/0 | no emPAI | n/a |
| 684 | gi\|33324437 | ATPase Na+/K+ transporting alpha 4 | 5.56 | 110872 | 80 | 3 | 3.9 | no emPAI | 0±0 | n/a |
| 685 | gi\|33341236 | polytrophin | 5.33 | 788036 | 148 | 7 | 3.8 | no emPAI | 0±0 | n/a |
| 686 | gi\|333944606 | Chain A, Crystal Structure Of Human L-3- Hydroxyacyl-Coa Dehydrogenase (Ec1.1.1.35) From Mitochondria At The Resolution 2.0 A, Northeast Structural Genomics Consortium Target Hr487, Mitochondrial Protein Partnership | 8.88 | 35290 | 96 | 4 | 20.7 | DIV/0 | no emPAI | n/a |
| 687 | gi\|33667109 | dehydrogenase/reductase SDR family member 2, mitochondrial isoform 1 | 10.01 | 31476 | 72 | 2 | 17.3 | 0±0 | no emPAI | n/a |
| 688 | gi\|338443 | beta-spectrin | 5.41 | 274461 | 95 | 4 | 3.9 | no emPAI | 0±0 | n/a |
| 689 | gi\|33873840 | SUPT16H protein, partial | 8.44 | 71952 | 84 | 3 | 7.3 | 0±0 | no emPAI | n/a |
| 690 | gi\|33988322 | MAST2 protein | 5.7 | 88689 | 77 | 2 | 6.4 | no emPAI | 0±0 | n/a |
| 691 | gi\|340545472 | mysterin | 6.04 | 591003 | 81 | 3 | 1.3 | no emPAI | 0±0 | n/a |
| 692 | gi\|340780678 | Chain A, Crystal Structure Of Human Set Domain-Containing Protein3 | 6.44 | 56695 | 97 | 4 | 16.9 | 0±0 | no emPAI | n/a |
| 693 | gi\|34192116 | Armadillo repeat containing 2 | 8.46 | 96021 | 80 | 3 | 6.6 | no emPAI | 0±0 | n/a |
| 694 | gi\|34226 | laminin A chain | 5.72 | 288131 | 89 | 3 | 3.8 | 0±0 | no emPAI | n/a |
| 695 | gi\|34303900 | KIAA1516 protein | 6.11 | 258785 | 88 | 3 | 2.5 | 0±0 | no emPAI | n/a |
| 696 | gi\|34328022 | KIAA1689 protein | 5.05 | 293912 | 80 | 3 | 2.2 | 0±0 | no emPAI | n/a |
| 697 | gi\|34335253 | disks large-associated protein 4 isoform a | 6.92 | 107501 | 100 | 4 | 8.8 | 0±0 | no emPAI | n/a |
| 698 | gi\|34339 | LDL-receptor related precursor (AA -19 to 4525) | 5.16 | 504245 | 95 | 4 | 4.2 | 0±0 | no emPAI | n/a |

**Supplementary Table 1.** Altered phosphoproteins in antibody-depedent enhancement (ADE) of DENV2-infected U937 cells (cont.)

| **No.** | **NCBI ID** | **Protein** | **pI** | **MW (Da)** | **Identification score** | **No. of matched peptides** | **%cov** | **Mock (Mean±SD)** | **Treated (Mean±SD)** | **Ratio (Treated/Mock)** |
| --- | --- | --- | --- | --- | --- | --- | --- | --- | --- | --- |
| 699 | gi\|34364998 | hypothetical protein | 8.56 | 55316 | 67 | 2 | 8.7 | 0±0 | no emPAI | n/a |
| 700 | gi\|34365016 | hypothetical protein | 5.44 | 257230 | 82 | 3 | 5 | no emPAI | 0±0 | n/a |
| 701 | gi\|34365308 | hypothetical protein | 9.25 | 79373 | 69 | 2 | 3.9 | 0±0 | no emPAI | n/a |
| 702 | gi\|343791837 | anti-HIV-1 immunoglobulin heavy chain variable region, partial | 9.1 | 15931 | 78 | 3 | 18.3 | 0±0 | no emPAI | n/a |
| 703 | gi\|343792807 | anti-HIV-1 immunoglobulin heavy chain variable region, partial | 9.69 | 14128 | 77 | 3 | 13.4 | 0±0 | no emPAI | n/a |
| 704 | gi\|343793157 | anti-HIV-1 immunoglobulin heavy chain variable region, partial | 8.96 | 14297 | 75 | 2 | 18.1 | 0±0 | no emPAI | n/a |
| 705 | gi\|345199283 | vigilin isoform b | 6.44 | 137885 | 67 | 2 | 1.8 | 0±0 | no emPAI | n/a |
| 706 | gi\|345199335 | cilia- and flagella-associated protein 57 isoform a | 5.75 | 148936 | 69 | 2 | 4.9 | 0±0 | no emPAI | n/a |
| 707 | gi\|3478639 | delta-adaptin, partial CDS | 8.66 | 126308 | 85 | 3 | 7.8 | no emPAI | 0±0 | n/a |
| 708 | gi\|347948577 | Chain B, Crystal Structure Of Active Caspase-6 In Complex With Ac-Veid-Cho Inhibitor | 8.93 | 12388 | 100 | 4 | 17.6 | no emPAI | 0±0 | n/a |
| 709 | gi\|35121 | NuMA protein | 5.72 | 236154 | 85 | 3 | 4.7 | 0±0 | no emPAI | n/a |
| 710 | gi\|3522970 | Trio | 5.96 | 323902 | 74 | 2 | 2.8 | no emPAI | 0±0 | n/a |
| 711 | gi\|355332900 | Chain A, Crystal Structure Of Armadillo Repeat Domain Of Apc | 6.67 | 37668 | 89 | 3 | 13.1 | no emPAI | 0±0 | n/a |
| 712 | gi\|359385708 | cilia- and flagella-associated protein 46 | 7.07 | 303308 | 87 | 3 | 2 | no emPAI | 0±0 | n/a |
| 713 | gi\|3603443 | fibrousheathin I | 5.97 | 94556 | 94 | 4 | 9.7 | no emPAI | 0±0 | n/a |
| 714 | gi\|365733568 | rho GTPase-activating protein 22 isoform 2 | 8.39 | 77408 | 96 | 4 | 8.9 | 0±0 | no emPAI | n/a |
| 715 | gi\|36953836 | neural interleukin 16 precursor protein | 8.41 | 141593 | 91 | 3 | 7.4 | 0±0 | no emPAI | n/a |
| 716 | gi\|37174 | thyroglobulin | 5.46 | 304581 | 70 | 2 | 3.4 | 0±0 | no emPAI | n/a |
| 717 | gi\|37227 | tenascin | 4.8 | 240565 | 92 | 4 | 4 | 0±0 | no emPAI | n/a |

**Supplementary Table 1.** Altered phosphoproteins in antibody-depedent enhancement (ADE) of DENV2-infected U937 cells (cont.)

| **No.** | **NCBI ID** | **Protein** | **pI** | **MW (Da)** | **Identification score** | **No. of matched peptides** | **%cov** | **Mock (Mean±SD)** | **Treated (Mean±SD)** | **Ratio (Treated/Mock)** |
| --- | --- | --- | --- | --- | --- | --- | --- | --- | --- | --- |
| 718 | gi\|37231 | DNA topoisomerase II | 8.22 | 182578 | 144 | 7 | 5.6 | 0±0 | no emPAI | n/a |
| 719 | gi\|37779176 | transformation-related protein 4 | 12.05 | 14158 | 74 | 2 | 29.8 | 0±0 | no emPAI | n/a |
| 720 | gi\|37788781 | phosphatase TPTE gamma isoform | 8.92 | 60150 | 69 | 2 | 6.2 | no emPAI | 0±0 | n/a |
| 721 | gi\|378548421 | RecName: Full=Kinesin-like protein KIF28P; AltName: Full=Kinesin-like protein 6 | 8.68 | 108185 | 81 | 3 | 8.1 | no emPAI | 0±0 | n/a |
| 722 | gi\|378925625 | titin isoform N2BA | 6.02 | 3813652 | 294 | 10 | 3.5 | no emPAI | 0±0 | n/a |
| 723 | gi\|37993630 | phosphatidylinositol transfer protein membrane-associated 1 | 5.64 | 134662 | 88 | 3 | 6 | no emPAI | 0±0 | n/a |
| 724 | gi\|38114865 | IFFO1 protein, partial | 4.35 | 33566 | 78 | 2 | 18.1 | no emPAI | 0±0 | n/a |
| 725 | gi\|3820492 | dopamine D2 receptor | 9.55 | 50596 | 80 | 3 | 6.8 | no emPAI | 0±0 | n/a |
| 726 | gi\|38348370 | insulin growth factor-like family member 1 precursor | 8.22 | 12354 | 70 | 2 | 19.1 | 0±0 | no emPAI | n/a |
| 727 | gi\|383867627 | truncated profilaggrin | 9.28 | 430162 | 127 | 6 | 4.9 | no emPAI | 0±0 | n/a |
| 728 | gi\|38570142 | MAGUK p55 subfamily member 5 isoform 1 | 5.77 | 77246 | 67 | 2 | 9 | no emPAI | 0±0 | n/a |
| 729 | gi\|38638917 | ankyrin repeat-containing protein | 6.66 | 297888 | 77 | 2 | 3.8 | no emPAI | 0±0 | n/a |
| 730 | gi\|38679971 | acetyl-CoA carboxylase 1 isoform 3 | 6.14 | 259521 | 86 | 3 | 3.5 | no emPAI | 0±0 | n/a |
| 731 | gi\|386864 | MT1A, partial | 8.38 | 6128 | 67 | 2 | 75.4 | 0±0 | no emPAI | n/a |
| 732 | gi\|38708321 | DNA helicase INO80 | 9.53 | 176643 | 95 | 3 | 5.8 | no emPAI | 0±0 | n/a |
| 733 | gi\|3882289 | KIAA0784 protein | 6.65 | 120235 | 71 | 2 | 5.7 | 0±0 | no emPAI | n/a |
| 734 | gi\|3882329 | KIAA0804 protein | 5.81 | 138168 | 80 | 3 | 3.4 | no emPAI | 0±0 | n/a |
| 735 | gi\|388604347 | Chain B, Crystal Structure Of The Spop Btb Domain Complexed With The Cul3 N- Terminal Domain | 5.47 | 42108 | 80 | 3 | 12.6 | no emPAI | 0±0 | n/a |
| 736 | gi\|390608639 | baculoviral IAP repeat-containing protein 2 isoform 2 | 6.02 | 64057 | 96 | 4 | 9.5 | no emPAI | 0±0 | n/a |

**Supplementary Table 1.** Altered phosphoproteins in antibody-depedent enhancement (ADE) of DENV2-infected U937 cells (cont.)

| **No.** | **NCBI ID** | **Protein** | **pI** | **MW (Da)** | **Identification score** | **No. of matched peptides** | **%cov** | **Mock (Mean±SD)** | **Treated (Mean±SD)** | **Ratio (Treated/Mock)** |
| --- | --- | --- | --- | --- | --- | --- | --- | --- | --- | --- |
| 737 | gi\|392933927 | transcriptional repressor CTCFL isoform 3 | 8.82 | 79958 | 73 | 2 | 8.3 | 0±0 | no emPAI | n/a |
| 738 | gi\|39963074 | PRP8 pre-mRNA processing factor 8 homolog (S. cerevisiae) | 8.94 | 273408 | 66 | 2 | 3 | no emPAI | 0±0 | n/a |
| 739 | gi\|39963533 | MAP7D3 protein, partial | 9.36 | 89971 | 82 | 3 | 9.7 | no emPAI | 0±0 | n/a |
| 740 | gi\|39985995 | transcription factor GTF2IRD2 beta isoform 1 | 5.15 | 24903 | 66 | 2 | 12.4 | 0±0 | no emPAI | n/a |
| 741 | gi\|4003503 | laminin beta-4 chain precursor | 5.93 | 193315 | 72 | 2 | 3.2 | no emPAI | 0±0 | n/a |
| 742 | gi\|40217847 | U5 small nuclear ribonucleoprotein 200 kDa helicase | 5.73 | 244353 | 97 | 4 | 3.7 | 0±0 | no emPAI | n/a |
| 743 | gi\|40225392 | ANAPC7 protein, partial | 4.96 | 50908 | 71 | 2 | 8.6 | no emPAI | 0±0 | n/a |
| 744 | gi\|402550544 | Chain A, Cid Of Human Rprd1b | 9.3 | 15495 | 103 | 4 | 12.6 | no emPAI | 0±0 | n/a |
| 745 | gi\|404211881 | obscurin isoform IC | 5.7 | 972395 | 159 | 8 | 3.3 | no emPAI | 0±0 | n/a |
| 746 | gi\|40556393 | protein Jade-1 isoform 1 | 8.2 | 95473 | 78 | 3 | 7.5 | no emPAI | 0±0 | n/a |
| 747 | gi\|405715 | giantin | 4.95 | 375848 | 76 | 2 | 2.8 | 0±0 | no emPAI | n/a |
| 748 | gi\|407099 | 190kD protein | 6.1 | 162351 | 75 | 2 | 5.2 | 0±0 | no emPAI | n/a |
| 749 | gi\|407727 | AML1-MTG8 fusion protein | 8.54 | 83126 | 106 | 4 | 8.9 | 0±0 | no emPAI | n/a |
| 750 | gi\|40786420 | immunoglobulin-like domain-containing receptor 2 precursor | 8.43 | 71155 | 76 | 2 | 7.7 | no emPAI | 0±0 | n/a |
| 751 | gi\|40788207 | KIAA0296 | 7.05 | 201709 | 91 | 3 | 4 | no emPAI | 0±0 | n/a |
| 752 | gi\|40788912 | KIAA0211 | 8.32 | 142775 | 88 | 3 | 6.1 | no emPAI | 0±0 | n/a |
| 753 | gi\|40788915 | KIAA0218 | 6.79 | 86813 | 80 | 3 | 12.9 | no emPAI | 0±0 | n/a |
| 754 | gi\|40789062 | KIAA0055 | 8.7 | 127704 | 88 | 3 | 7.8 | no emPAI | 0±0 | n/a |
| 755 | gi\|40789066 | KIAA0004 | 5.56 | 150325 | 98 | 4 | 4.1 | no emPAI | 0±0 | n/a |
| 756 | gi\|407954645 | malignant cell derived RNA helicase | 9.67 | 81986 | 95 | 4 | 8.9 | no emPAI | 0±0 | n/a |
| 757 | gi\|40795897 | hornerin precursor | 10.04 | 282199 | 85 | 3 | 4.2 | no emPAI | 0±0 | n/a |

**Supplementary Table 1.** Altered phosphoproteins in antibody-depedent enhancement (ADE) of DENV2-infected U937 cells (cont.)

| **No.** | **NCBI ID** | **Protein** | **pI** | **MW (Da)** | **Identification score** | **No. of matched peptides** | **%cov** | **Mock (Mean±SD)** | **Treated (Mean±SD)** | **Ratio (Treated/Mock)** |
| --- | --- | --- | --- | --- | --- | --- | --- | --- | --- | --- |
| 758 | gi\|40889610 | Chain A, Crystal Structure Of Human Tryptophanyl-Trna Synthetase | 6.03 | 53466 | 97 | 4 | 15.1 | no emPAI | 0±0 | n/a |
| 759 | gi\|4093196 | attractin-2 | 6.65 | 141369 | 98 | 4 | 4.2 | 0±0 | no emPAI | n/a |
| 760 | gi\|409426 | very low density lipoprotein receptor | 4.64 | 96059 | 82 | 3 | 8.8 | 0±0 | no emPAI | n/a |
| 761 | gi\|410169930 | PREDICTED: splicing factor, arginine/serine-rich 19-like | 12.17 | 24708 | 79 | 3 | 17.4 | 0±0 | no emPAI | n/a |
| 762 | gi\|4104413 | Eph-like receptor tyrosine kinase hEphB1b | 6.7 | 108869 | 69 | 2 | 4.3 | no emPAI | 0±0 | n/a |
| 763 | gi\|41054864 | regulating synaptic membrane exocytosis protein 1 isoform 1 | 9.68 | 188956 | 116 | 4 | 7.3 | no emPAI | 0±0 | n/a |
| 764 | gi\|41322908 | plectin isoform 1e | 5.61 | 513393 | 275 | 10 | 5.2 | no emPAI | 0±0 | n/a |
| 765 | gi\|415821 | antigen of the monoclonal antibody Ki-67 | 9.51 | 319300 | 154 | 7 | 6.1 | 0±0 | no emPAI | n/a |
| 766 | gi\|42544121 | vacuolar protein sorting-associated protein 13C isoform 1A | 6.17 | 417013 | 82 | 3 | 3.3 | 0±0 | no emPAI | n/a |
| 767 | gi\|42558476 | FER-1-like protein 4 | 5.68 | 230046 | 84 | 3 | 3.4 | 0±0 | no emPAI | n/a |
| 768 | gi\|4262541 | archvillin | 6.55 | 247553 | 105 | 4 | 4.3 | 0±0 | no emPAI | n/a |
| 769 | gi\|42627291 | hypothetical protein | 10.06 | 131746 | 114 | 4 | 10.1 | no emPAI | 0±0 | n/a |
| 770 | gi\|428698118 | Chain A, Crystal Structure Of Human D-xylulokinase In Complex With D- Xylulose And Adenosine Diphosphate | 5.55 | 58212 | 68 | 2 | 13 | 0±0 | no emPAI | n/a |
| 771 | gi\|430765482 | tumor suppressor p53, partial | 8.35 | 11885 | 72 | 2 | 14 | no emPAI | 0±0 | n/a |
| 772 | gi\|4322304 | translation initiation factor IF2 | 5.39 | 138860 | 66 | 2 | 3.6 | no emPAI | 0±0 | n/a |
| 773 | gi\|4335941 | leucine aminopeptidase | 7.58 | 56014 | 99 | 5 | 17 | no emPAI | 0±0 | n/a |
| 774 | gi\|434753 | KIAA0030 | 5.32 | 102766 | 123 | 5 | 7.4 | no emPAI | 0±0 | n/a |
| 775 | gi\|434765 | ORF | 9.49 | 74466 | 105 | 4 | 16.3 | no emPAI | 0±0 | n/a |
| 776 | gi\|437972 | fibrillin-2 | 4.73 | 314131 | 102 | 4 | 4 | 0±0 | no emPAI | n/a |

**Supplementary Table 1.** Altered phosphoproteins in antibody-depedent enhancement (ADE) of DENV2-infected U937 cells (cont.)

| **No.** | **NCBI ID** | **Protein** | **pI** | **MW (Da)** | **Identification score** | **No. of matched peptides** | **%cov** | **Mock (Mean±SD)** | **Treated (Mean±SD)** | **Ratio (Treated/Mock)** |
| --- | --- | --- | --- | --- | --- | --- | --- | --- | --- | --- |
| 777 | gi\|44680114 | proteasome assembly chaperone 1 isoform b | 7.96 | 30268 | 68 | 2 | 21.7 | 0±0 | no emPAI | n/a |
| 778 | gi\|44917604 | SLIT-ROBO Rho GTPase-activating protein 1 | 6.36 | 124186 | 87 | 2 | 4.7 | 0±0 | no emPAI | n/a |
| 779 | gi\|4501913 | disintegrin and metalloproteinase domain-containing protein 23 preproprotein | 7.85 | 91867 | 72 | 2 | 5.4 | no emPAI | 0±0 | n/a |
| 780 | gi\|4503037 | protein CREG1 precursor | 7.05 | 24059 | 95 | 4 | 28.2 | no emPAI | 0±0 | n/a |
| 781 | gi\|4504125 | glutamate receptor ionotropic, NMDA 2A isoform 1 precursor | 6.67 | 165177 | 65 | 2 | 5.1 | 0±0 | no emPAI | n/a |
| 782 | gi\|4504779 | integrin beta-8 precursor | 7.06 | 85575 | 80 | 3 | 7.8 | 0±0 | no emPAI | n/a |
| 783 | gi\|4504831 | potassium voltage-gated channel subfamily H member 1 isoform 2 | 7.95 | 108528 | 69 | 2 | 4.8 | no emPAI | 0±0 | n/a |
| 784 | gi\|4505531 | oxysterol-binding protein 1 | 6.9 | 89365 | 80 | 3 | 8.6 | no emPAI | 0±0 | n/a |
| 785 | gi\|4506787 | ras GTPase-activating-like protein IQGAP1 | 6.08 | 189134 | 96 | 4 | 4.4 | 0±0 | no emPAI | n/a |
| 786 | gi\|4506857 | fractalkine isoform 1 precursor | 6.08 | 42176 | 67 | 2 | 7.3 | 0±0 | no emPAI | n/a |
| 787 | gi\|4507651 | tropomyosin alpha-4 chain isoform Tpm4.2cy | 4.67 | 28504 | 78 | 2 | 20.2 | no emPAI | 0±0 | n/a |
| 788 | gi\|4530441 | thyroid hormone receptor-associated protein complex component TRAP150 | 10.16 | 108629 | 99 | 4 | 6.2 | 0±0 | no emPAI | n/a |
| 789 | gi\|4557565 | DNA excision repair protein ERCC-6 | 8.28 | 168311 | 75 | 2 | 4.4 | 0±0 | no emPAI | n/a |
| 790 | gi\|4566495 | topoisomerase I-binding RS protein | 9.57 | 118938 | 68 | 2 | 7.4 | 0±0 | no emPAI | n/a |
| 791 | gi\|457879 | voltage-gated sodium channel | 5.53 | 227770 | 77 | 2 | 3.1 | 0±0 | no emPAI | n/a |
| 792 | gi\|4589590 | KIAA0973 protein | 8.83 | 172166 | 81 | 2 | 3.9 | 0±0 | no emPAI | n/a |
| 793 | gi\|459214610 | SLC34A2-ROS1 fusion protein | 6.79 | 141210 | 68 | 2 | 2.8 | 0±0 | no emPAI | n/a |
| 794 | gi\|459214612 | CD74-ROS1_C6;R32 fusion protein | 6.36 | 90949 | 68 | 2 | 4.6 | 0±0 | no emPAI | n/a |

**Supplementary Table 1.** Altered phosphoproteins in antibody-depedent enhancement (ADE) of DENV2-infected U937 cells (cont.)

| **No.** | **NCBI ID** | **Protein** | **pI** | **MW (Da)** | **Identification score** | **No. of matched peptides** | **%cov** | **Mock (Mean±SD)** | **Treated (Mean±SD)** | **Ratio (Treated/Mock)** |
| --- | --- | --- | --- | --- | --- | --- | --- | --- | --- | --- |
| 795 | gi\|459215033 | zinc finger protein 541 | 8.28 | 145496 | 92 | 4 | 8.2 | no emPAI | 0±0 | n/a |
| 796 | gi\|459642365 | dynein heavy chain 11, axonemal | 6.03 | 520038 | 119 | 5 | 3.8 | 0±0 | no emPAI | n/a |
| 797 | gi\|46250431 | NFKB repressing factor | 8.94 | 77655 | 85 | 3 | 7.5 | no emPAI | 0±0 | n/a |
| 798 | gi\|46277638 | NMDA receptor subunit 3B | 9.32 | 112771 | 76 | 2 | 5.8 | no emPAI | 0±0 | n/a |
| 799 | gi\|46325239 | microcephalin | 7.25 | 41968 | 98 | 4 | 14.1 | 0±0 | no emPAI | n/a |
| 800 | gi\|46621647 | PHLDB2 protein, partial | 7.17 | 125788 | 127 | 6 | 9.3 | no emPAI | 0±0 | n/a |
| 801 | gi\|467977 | N-ethylmaleimide-sensitive factor, partial | 6.28 | 83344 | 88 | 3 | 8.4 | no emPAI | 0±0 | n/a |
| 802 | gi\|472339087 | ankyrin repeat domain-containing protein 62 | 6.25 | 106379 | 68 | 2 | 4.8 | 0±0 | no emPAI | n/a |
| 803 | gi\|474982 | C3G protein | 5.6 | 120534 | 85 | 3 | 4.3 | 0±0 | no emPAI | n/a |
| 804 | gi\|4757966 | testis-specific chromodomain protein Y 1 isoform b | 9.39 | 61978 | 73 | 2 | 11.6 | no emPAI | 0±0 | n/a |
| 805 | gi\|4758128 | serine/threonine-protein kinase DCLK1 isoform 1 | 8.84 | 81049 | 98 | 4 | 8.4 | no emPAI | 0±0 | n/a |
| 806 | gi\|4758650 | kinesin heavy chain isoform 5C | 5.86 | 109427 | 67 | 2 | 4.8 | no emPAI | 0±0 | n/a |
| 807 | gi\|4759310 | bestrophin-1 isoform 1 | 6.43 | 67640 | 71 | 2 | 4.3 | no emPAI | 0±0 | n/a |
| 808 | gi\|4759344 | centromere/kinetochore protein zw10 homolog | 5.89 | 88773 | 66 | 2 | 5.5 | 0±0 | no emPAI | n/a |
| 809 | gi\|4826902 | serpin B10 | 5.8 | 45374 | 95 | 4 | 14.1 | 0±0 | no emPAI | n/a |
| 810 | gi\|48525351 | calcium-independent phospholipase A2-gamma isoform 1 | 9.31 | 88421 | 126 | 6 | 10.7 | no emPAI | 0±0 | n/a |
| 811 | gi\|4884132 | hypothetical protein | 6.66 | 55597 | 87 | 3 | 13.2 | 0±0 | no emPAI | n/a |
| 812 | gi\|49257509 | Hypothetical protein LOC100132288 | 5.6 | 14841 | 80 | 3 | 29.5 | 0±0 | no emPAI | n/a |
| 813 | gi\|49456619 | CNN2 | 6.95 | 33707 | 69 | 2 | 12.3 | 0±0 | no emPAI | n/a |
| 814 | gi\|495866 | collagen type VII | 5.89 | 292164 | 92 | 4 | 2.9 | 0±0 | no emPAI | n/a |
| 815 | gi\|49660012 | sarcomeric tropomyosin kappa | 4.65 | 32631 | 65 | 2 | 14.1 | no emPAI | 0±0 | n/a |

**Supplementary Table 1.** Altered phosphoproteins in antibody-depedent enhancement (ADE) of DENV2-infected U937 cells (cont.)

| **No.** | **NCBI ID** | **Protein** | **pI** | **MW (Da)** | **Identification score** | **No. of matched peptides** | **%cov** | **Mock (Mean±SD)** | **Treated (Mean±SD)** | **Ratio (Treated/Mock)** |
| --- | --- | --- | --- | --- | --- | --- | --- | --- | --- | --- |
| 816 | gi\|5032115 | protein sprouty homolog 2 | 8.78 | 34666 | 74 | 2 | 7.9 | no emPAI | 0±0 | n/a |
| 817 | gi\|5032189 | tumor suppressor p53-binding protein 1 isoform 3 | 4.62 | 213443 | 71 | 2 | 2.7 | no emPAI | 0±0 | n/a |
| 818 | gi\|5032315 | dystrophin Dp427p2 isoform | 5.6 | 412144 | 98 | 4 | 2.5 | 0±0 | no emPAI | n/a |
| 819 | gi\|50348617 | microtubule-associated tumor suppressor 1 isoform 2 | 6.55 | 135577 | 77 | 3 | 6 | 0±0 | no emPAI | n/a |
| 820 | gi\|50348626 | microtubule-associated tumor suppressor 1 isoform 4 | 8.53 | 58952 | 101 | 4 | 14.3 | 0±0 | no emPAI | n/a |
| 821 | gi\|5051743 | Centrosome- and Golgi-localized PKN-associated protein (CG-NAP) | 4.95 | 451560 | 206 | 10 | 4.4 | no emPAI | 0±0 | n/a |
| 822 | gi\|505538 | Dsc1b precursor | 5.25 | 99982 | 82 | 3 | 5.5 | 0±0 | no emPAI | n/a |
| 823 | gi\|50659098 | inactive serine protease PAMR1 isoform a precursor | 7.57 | 81889 | 99 | 4 | 8.4 | no emPAI | 0±0 | n/a |
| 824 | gi\|508185 | voltage-dependent L-type Ca channel alpha 1 subunit | 6.3 | 238923 | 66 | 2 | 2.7 | 0±0 | no emPAI | n/a |
| 825 | gi\|50949443 | hypothetical protein | 8.5 | 63167 | 84 | 3 | 11 | no emPAI | 0±0 | n/a |
| 826 | gi\|50949586 | hypothetical protein | 6.03 | 96137 | 86 | 3 | 4.9 | 0±0 | no emPAI | n/a |
| 827 | gi\|50949603 | hypothetical protein | 5.83 | 158036 | 103 | 4 | 5.2 | no emPAI | 0±0 | n/a |
| 828 | gi\|50949951 | hypothetical protein | 5.74 | 125296 | 67 | 2 | 4.3 | 0±0 | no emPAI | n/a |
| 829 | gi\|50950047 | hypothetical protein | 8.62 | 89086 | 91 | 3 | 7.8 | no emPAI | 0±0 | n/a |
| 830 | gi\|5106956 | FH1/FH2 domain-containing protein FHOS | 6.07 | 126419 | 94 | 4 | 4.6 | 0±0 | no emPAI | n/a |
| 831 | gi\|51094908 | similar to hypothetical protein 4932412H11 | 7.61 | 74107 | 85 | 3 | 7.4 | no emPAI | 0±0 | n/a |
| 832 | gi\|51094935 | LOC401385 | 11.65 | 27323 | 68 | 2 | 18.3 | no emPAI | 0±0 | n/a |
| 833 | gi\|512125774 | Chain A, A Cryptic Tog Domain With A Distinct Architecture Underlies Clasp- Dependent Bipolar Spindle Formation | 7.02 | 30617 | 69 | 2 | 10.3 | no emPAI | 0±0 | n/a |

**Supplementary Table 1.** Altered phosphoproteins in antibody-depedent enhancement (ADE) of DENV2-infected U937 cells (cont.)

| **No.** | **NCBI ID** | **Protein** | **pI** | **MW (Da)** | **Identification score** | **No. of matched peptides** | **%cov** | **Mock (Mean±SD)** | **Treated (Mean±SD)** | **Ratio (Treated/Mock)** |
| --- | --- | --- | --- | --- | --- | --- | --- | --- | --- | --- |
| 834 | gi\|512126376 | DIET1 | 5.06 | 240897 | 111 | 4 | 3.4 | 0±0 | no emPAI | n/a |
| 835 | gi\|51476214 | hypothetical protein | 6.25 | 144177 | 70 | 2 | 4.2 | 0±0 | no emPAI | n/a |
| 836 | gi\|51476507 | hypothetical protein | 6.1 | 133790 | 68 | 2 | 4 | no emPAI | 0±0 | n/a |
| 837 | gi\|51491277 | hypothetical protein | 8.14 | 273261 | 88 | 3 | 5 | 0±0 | no emPAI | n/a |
| 838 | gi\|515031 | protein-tyrosine-phosphatase | 6.01 | 274805 | 67 | 2 | 2.8 | 0±0 | no emPAI | n/a |
| 839 | gi\|51558724 | A disintegrin and metalloproteinase with thrombospondin motifs 12 preproprotein | 8.25 | 177560 | 77 | 2 | 4.8 | 0±0 | no emPAI | n/a |
| 840 | gi\|516758 | diacylglycerol kinase gamma | 6.29 | 88939 | 83 | 3 | 6.4 | no emPAI | 0±0 | n/a |
| 841 | gi\|519668683 | RecName: Full=Putative zinc finger protein 66; AltName: Full=Zinc finger protein 66 pseudogene | 9.46 | 65831 | 80 | 3 | 13.8 | no emPAI | 0±0 | n/a |
| 842 | gi\|52545752 | hypothetical protein | 9.04 | 96117 | 77 | 3 | 4.2 | no emPAI | 0±0 | n/a |
| 843 | gi\|52545857 | hypothetical protein | 9.05 | 108747 | 103 | 4 | 10.3 | no emPAI | 0±0 | n/a |
| 844 | gi\|5262584 | hypothetical protein | 8.21 | 74548 | 81 | 3 | 12.6 | 0±0 | no emPAI | n/a |
| 845 | gi\|530360577 | PREDICTED: arginine-glutamic acid dipeptide repeats protein isoform X3 | 7.6 | 141453 | 90 | 3 | 4.2 | no emPAI | 0±0 | n/a |
| 846 | gi\|530365709 | PREDICTED: zinc finger CCCH domain-containing protein 11A isoform X5 | 8.83 | 51602 | 92 | 4 | 12.7 | no emPAI | 0±0 | n/a |
| 847 | gi\|530367230 | PREDICTED: pleckstrin homology domain-containing family H member 2 isoform X1 | 5.74 | 87555 | 79 | 3 | 9.4 | no emPAI | 0±0 | n/a |
| 848 | gi\|530367595 | PREDICTED: latent-transforming growth factor beta-binding protein 1 isoform X5 | 7.71 | 134017 | 86 | 3 | 4.4 | no emPAI | 0±0 | n/a |
| 849 | gi\|530368633 | PREDICTED: ankyrin repeat domain-containing protein 36A isoform X2 | 8.97 | 166735 | 72 | 2 | 4.1 | no emPAI | 0±0 | n/a |

**Supplementary Table 1.** Altered phosphoproteins in antibody-depedent enhancement (ADE) of DENV2-infected U937 cells (cont.)

| **No.** | **NCBI ID** | **Protein** | **pI** | **MW (Da)** | **Identification score** | **No. of matched peptides** | **%cov** | **Mock (Mean±SD)** | **Treated (Mean±SD)** | **Ratio (Treated/Mock)** |
| --- | --- | --- | --- | --- | --- | --- | --- | --- | --- | --- |
| 850 | gi\|530369213 | PREDICTED: nephrocystin-1 isoform X1 | 5.34 | 87372 | 66 | 2 | 5.3 | no emPAI | 0±0 | n/a |
| 851 | gi\|530369278 | PREDICTED: protein IWS1 homolog isoform X1 | 4.58 | 92416 | 95 | 4 | 9.6 | no emPAI | 0±0 | n/a |
| 852 | gi\|530369358 | PREDICTED: RANBP2-like and GRIP domain-containing protein 8 isoform X3 | 6.11 | 186659 | 90 | 3 | 4.6 | 0±0 | no emPAI | n/a |
| 853 | gi\|530369947 | PREDICTED: uridine phosphorylase 2 isoform X2 | 6.75 | 31304 | 75 | 2 | 18.9 | 0±0 | no emPAI | n/a |
| 854 | gi\|530370359 | PREDICTED: microtubule-associated protein 2 isoform X5 | 4.85 | 211325 | 78 | 2 | 4.4 | 0±0 | no emPAI | n/a |
| 855 | gi\|530370475 | PREDICTED: nebulin isoform X27 | 9.07 | 928636 | 116 | 4 | 3.4 | 0±0 | no emPAI | n/a |
| 856 | gi\|530370760 | PREDICTED: sodium channel protein type 9 subunit alpha isoform X1 | 6.55 | 226226 | 120 | 5 | 5.6 | no emPAI | 0±0 | n/a |
| 857 | gi\|530370802 | PREDICTED: ras-associated and pleckstrin homology domains-containing protein 1 isoform X1 | 9 | 141091 | 70 | 2 | 5.3 | no emPAI | 0±0 | n/a |
| 858 | gi\|530371485 | PREDICTED: leucine-rich repeat flightless-interacting protein 1 isoform X13 | 4.69 | 105817 | 77 | 3 | 5.1 | 0±0 | no emPAI | n/a |
| 859 | gi\|530376284 | PREDICTED: biorientation of chromosomes in cell division protein 1-like 1 isoform X1 | 5.05 | 335341 | 95 | 4 | 3.9 | no emPAI | 0±0 | n/a |
| 860 | gi\|530376585 | PREDICTED: LIM and calponin homology domains-containing protein 1 isoform X8 | 6.28 | 172941 | 130 | 6 | 9.6 | 0±0 | no emPAI | n/a |
| 861 | gi\|530376595 | PREDICTED: LIM and calponin homology domains-containing protein 1 isoform X13 | 6.19 | 152495 | 144 | 6 | 10.8 | 0±0 | no emPAI | n/a |
| 862 | gi\|530377140 | PREDICTED: WD repeat-containing protein 17 isoform X4 | 5.96 | 135459 | 80 | 3 | 6.9 | 0±0 | no emPAI | n/a |

**Supplementary Table 1.** Altered phosphoproteins in antibody-depedent enhancement (ADE) of DENV2-infected U937 cells (cont.)

| **No.** | **NCBI ID** | **Protein** | **pI** | **MW (Da)** | **Identification score** | **No. of matched peptides** | **%cov** | **Mock (Mean±SD)** | **Treated (Mean±SD)** | **Ratio (Treated/Mock)** |
| --- | --- | --- | --- | --- | --- | --- | --- | --- | --- | --- |
| 863 | gi\|530377410 | PREDICTED: WD repeat and FYVE domain-containing protein 3 isoform X1 | 6.31 | 396951 | 79 | 3 | 3 | 0±0 | no emPAI | n/a |
| 864 | gi\|530378259 | PREDICTED: sorting nexin-25 isoform X1 | 6.47 | 114138 | 67 | 2 | 5.9 | no emPAI | 0±0 | n/a |
| 865 | gi\|530378724 | PREDICTED: dynein heavy chain 5, axonemal isoform X1 | 5.73 | 526805 | 112 | 4 | 2.8 | no emPAI | 0±0 | n/a |
| 866 | gi\|530379749 | PREDICTED: dmX-like protein 1 isoform X5 | 5.98 | 327482 | 96 | 4 | 2.4 | 0±0 | no emPAI | n/a |
| 867 | gi\|530379795 | PREDICTED: inositol hexakisphosphate and diphosphoinositol-pentakisphosphate kinase 2 isoform X4 | 8.36 | 134084 | 79 | 3 | 7.7 | 0±0 | no emPAI | n/a |
| 868 | gi\|530379906 | PREDICTED: colorectal mutant cancer protein isoform X1 | 5.35 | 95288 | 94 | 4 | 7.9 | 0±0 | no emPAI | n/a |
| 869 | gi\|530382406 | PREDICTED: bone morphogenetic protein 5 isoform X3 | 9.59 | 39491 | 67 | 2 | 11.2 | no emPAI | 0±0 | n/a |
| 870 | gi\|530382418 | PREDICTED: dystonin isoform X1 | 5.22 | 887829 | 161 | 8 | 3.2 | no emPAI | 0±0 | n/a |
| 871 | gi\|530382446 | PREDICTED: dystonin isoform X15 | 5.12 | 825471 | 129 | 6 | 2.2 | 0±0 | no emPAI | n/a |
| 872 | gi\|530382843 | PREDICTED: cullin-7 isoform X1 | 5.7 | 196369 | 93 | 4 | 4.3 | no emPAI | 0±0 | n/a |
| 873 | gi\|530383083 | PREDICTED: regulating synaptic membrane exocytosis protein 1 isoform X7 | 9.34 | 150115 | 114 | 4 | 8 | no emPAI | 0±0 | n/a |
| 874 | gi\|530384572 | PREDICTED: radial spoke head 10 homolog B isoform X2 | 7.06 | 68646 | 82 | 3 | 7.3 | no emPAI | 0±0 | n/a |
| 875 | gi\|530386305 | PREDICTED: coiled-coil domain-containing protein 136 isoform X2 | 5.07 | 139306 | 101 | 4 | 6.6 | no emPAI | 0±0 | n/a |
| 876 | gi\|530389736 | PREDICTED: FRAS1-related extracellular matrix protein 1 isoform X2 | 8.13 | 80293 | 79 | 3 | 7.3 | 0±0 | no emPAI | n/a |
| 877 | gi\|530390557 | PREDICTED: disabled homolog 2-interacting protein isoform X2 | 8.93 | 128956 | 95 | 4 | 5.4 | no emPAI | 0±0 | n/a |

**Supplementary Table 1.** Altered phosphoproteins in antibody-depedent enhancement (ADE) of DENV2-infected U937 cells (cont.)

| **No.** | **NCBI ID** | **Protein** | **pI** | **MW (Da)** | **Identification score** | **No. of matched peptides** | **%cov** | **Mock (Mean±SD)** | **Treated (Mean±SD)** | **Ratio (Treated/Mock)** |
| --- | --- | --- | --- | --- | --- | --- | --- | --- | --- | --- |
| 878 | gi\|530390803 | PREDICTED: structural maintenance of chromosomes protein 5 isoform X2 | 8.56 | 125918 | 68 | 2 | 7 | 0±0 | no emPAI | n/a |
| 879 | gi\|530390905 | PREDICTED: cip1-interacting zinc finger protein isoform X1 | 6.65 | 110327 | 68 | 2 | 3.6 | no emPAI | 0±0 | n/a |
| 880 | gi\|530392207 | PREDICTED: protein FAM208B isoform X1 | 5.74 | 296481 | 168 | 8 | 4.6 | no emPAI | 0±0 | n/a |
| 881 | gi\|530392267 | PREDICTED: sickle tail protein homolog isoform X5 | 6.68 | 214651 | 102 | 4 | 5 | 0±0 | no emPAI | n/a |
| 882 | gi\|530392887 | PREDICTED: pro-neuregulin-3, membrane-bound isoform isoform X1 | 7.79 | 77852 | 109 | 4 | 7.5 | 0±0 | no emPAI | n/a |
| 883 | gi\|530394148 | PREDICTED: F-box/WD repeat-containing protein 4 isoform X1 | 9.44 | 66882 | 69 | 2 | 12 | no emPAI | 0±0 | n/a |
| 884 | gi\|530396566 | PREDICTED: C2 domain-containing protein 3 isoform X1 | 6.69 | 260313 | 109 | 4 | 4.3 | 0±0 | no emPAI | n/a |
| 885 | gi\|530396906 | PREDICTED: synaptotagmin-like protein 2 isoform X1 | 5.85 | 247164 | 69 | 2 | 4.1 | 0±0 | no emPAI | n/a |
| 886 | gi\|530399429 | PREDICTED: transmembrane and TPR repeat-containing protein 1 isoform X1 | 9.06 | 108699 | 74 | 2 | 4.2 | 0±0 | no emPAI | n/a |
| 887 | gi\|530402285 | PREDICTED: neurobeachin isoform X5 | 5.74 | 295038 | 100 | 4 | 3.7 | 0±0 | no emPAI | n/a |
| 888 | gi\|530405884 | PREDICTED: unconventional myosin-Va isoform X2 | 8.76 | 218600 | 110 | 4 | 4.8 | no emPAI | 0±0 | n/a |
| 889 | gi\|530406265 | PREDICTED: ras-specific guanine nucleotide-releasing factor 1 isoform X1 | 8.44 | 144651 | 70 | 2 | 5.9 | no emPAI | 0±0 | n/a |
| 890 | gi\|530406610 | PREDICTED: probable E3 ubiquitin-protein ligase HERC1 isoform X6 | 5.71 | 524891 | 124 | 5 | 2.7 | 0±0 | no emPAI | n/a |
| 891 | gi\|530406713 | PREDICTED: kinesin-like protein KIF23 isoform X2 | 8.62 | 106443 | 70 | 2 | 6.8 | no emPAI | 0±0 | n/a |
| 892 | gi\|530406998 | PREDICTED: A-kinase anchor protein 13 isoform X11 | 5.13 | 292181 | 65 | 2 | 1.7 | 0±0 | no emPAI | n/a |

**Supplementary Table 1.** Altered phosphoproteins in antibody-depedent enhancement (ADE) of DENV2-infected U937 cells (cont.)

| **No.** | **NCBI ID** | **Protein** | **pI** | **MW (Da)** | **Identification score** | **No. of matched peptides** | **%cov** | **Mock (Mean±SD)** | **Treated (Mean±SD)** | **Ratio (Treated/Mock)** |
| --- | --- | --- | --- | --- | --- | --- | --- | --- | --- | --- |
| 893 | gi\|530407006 | PREDICTED: A-kinase anchor protein 13 isoform X15 | 6.88 | 171825 | 80 | 3 | 3 | 0±0 | no emPAI | n/a |
| 894 | gi\|530407857 | PREDICTED: serine/arginine repetitive matrix protein 2 isoform X2 | 12.05 | 299351 | 243 | 10 | 9.9 | no emPAI | 0±0 | n/a |
| 895 | gi\|530410559 | PREDICTED: nucleoredoxin isoform X2 | 4.88 | 36990 | 67 | 2 | 5.2 | 0±0 | no emPAI | n/a |
| 896 | gi\|530411554 | PREDICTED: cyclin N-terminal domain-containing protein 1 isoform X1 | 8.83 | 35965 | 73 | 3 | 6.2 | 0±0 | 0±0 | n/a |
| 897 | gi\|530413745 | PREDICTED: SET-binding protein isoform X2 | 9.76 | 169140 | 87 | 3 | 5.7 | no emPAI | 0±0 | n/a |
| 898 | gi\|530413971 | PREDICTED: putative Polycomb group protein ASXL3 isoform X1 | 5.82 | 241951 | 68 | 2 | 3.8 | no emPAI | 0±0 | n/a |
| 899 | gi\|530414289 | PREDICTED: transcription factor 4 isoform X2 | 8.73 | 80938 | 77 | 3 | 8.5 | 0±0 | 0±0 | n/a |
| 900 | gi\|530415042 | PREDICTED: dedicator of cytokinesis protein 6 isoform X1 | 6.39 | 236337 | 98 | 4 | 4.5 | no emPAI | 0±0 | n/a |
| 901 | gi\|530420806 | PREDICTED: protein DENND6B isoform X2 | 9.3 | 71434 | 91 | 3 | 7.3 | no emPAI | 0±0 | n/a |
| 902 | gi\|530421811 | PREDICTED: transcription initiation factor TFIID subunit 1 isoform X2 | 4.92 | 218279 | 95 | 4 | 4.5 | 0±0 | no emPAI | n/a |
| 903 | gi\|530422336 | PREDICTED: dedicator of cytokinesis protein 11 isoform X2 | 7.51 | 235728 | 86 | 3 | 3.9 | 0±0 | no emPAI | n/a |
| 904 | gi\|530426352 | PREDICTED: E3 ubiquitin-protein ligase HUWE1 isoform X5 | 5.08 | 479853 | 153 | 7 | 6.2 | no emPAI | 0±0 | n/a |
| 905 | gi\|530427149 | PREDICTED: rap guanine nucleotide exchange factor 1 isoform X2 | 5.6 | 139819 | 120 | 5 | 7.8 | no emPAI | 0±0 | n/a |
| 906 | gi\|530427503 | PREDICTED: heterogeneous nuclear ribonucleoprotein M isoform X2 | 8.74 | 75586 | 113 | 4 | 15.6 | no emPAI | 0±0 | n/a |

**Supplementary Table 1.** Altered phosphoproteins in antibody-depedent enhancement (ADE) of DENV2-infected U937 cells (cont.)

| **No.** | **NCBI ID** | **Protein** | **pI** | **MW (Da)** | **Identification score** | **No. of matched peptides** | **%cov** | **Mock (Mean±SD)** | **Treated (Mean±SD)** | **Ratio (Treated/Mock)** |
| --- | --- | --- | --- | --- | --- | --- | --- | --- | --- | --- |
| 907 | gi\|530436592 | PREDICTED: arf-GAP with GTPase, ANK repeat and PH domain-containing protein 5-like isoform X3 | 6.2 | 75632 | 71 | 2 | 10.1 | no emPAI | 0±0 | n/a |
| 908 | gi\|532056 | protein-tyrosine-phosphatase | 8 | 133203 | 68 | 2 | 2.3 | 0±0 | no emPAI | n/a |
| 909 | gi\|532691770 | proteasome subunit alpha type-6 isoform b | 5.18 | 18803 | 66 | 2 | 22.8 | no emPAI | 0±0 | n/a |
| 910 | gi\|5360204 | A-kinase anchor protein | 4.9 | 256505 | 109 | 4 | 3.6 | no emPAI | 0±0 | n/a |
| 911 | gi\|537361056 | minor histocompatibility protein HA-1 isoform 4 | 5.58 | 112401 | 73 | 3 | 8.7 | no emPAI | 0±0 | n/a |
| 912 | gi\|53830059 | anchor protein | 6.37 | 452956 | 142 | 6 | 2.9 | 0±0 | no emPAI | n/a |
| 913 | gi\|54112382 | lysine-specific demethylase 2B isoform a | 8.85 | 152517 | 74 | 2 | 7 | 0±0 | no emPAI | n/a |
| 914 | gi\|54112403 | chromodomain-helicase-DNA-binding protein 7 | 5.95 | 335717 | 67 | 2 | 2.8 | no emPAI | 0±0 | n/a |
| 915 | gi\|5419846 | hypothetical protein | 6.01 | 164374 | 75 | 2 | 6.3 | 0±0 | no emPAI | n/a |
| 916 | gi\|54292123 | lysosomal-trafficking regulator | 6.15 | 428866 | 131 | 6 | 5.9 | no emPAI | 0±0 | n/a |
| 917 | gi\|54292714 | MTM | 8.37 | 4995 | 81 | 3 | 83.7 | 0±0 | no emPAI | n/a |
| 918 | gi\|544346129 | SUN domain-containing ossification factor isoform 3 precursor | 5.33 | 98223 | 68 | 2 | 4.9 | 0±0 | no emPAI | n/a |
| 919 | gi\|544346136 | SPATS2-like protein isoform d | 9.69 | 65111 | 77 | 3 | 9 | 0±0 | no emPAI | n/a |
| 920 | gi\|544346311 | oxysterol-binding protein 2 isoform c | 6.38 | 84691 | 92 | 4 | 12.8 | no emPAI | 0±0 | n/a |
| 921 | gi\|544399844 | PREDICTED: SLIT-ROBO Rho GTPase-activating protein 2 isoform X2 [Macaca fascicularis] | 6.39 | 110557 | 66 | 2 | 3.7 | no emPAI | 0±0 | n/a |
| 922 | gi\|548923931 | rab effector MyRIP isoform b | 5.57 | 88511 | 86 | 3 | 8.4 | no emPAI | 0±0 | n/a |
| 923 | gi\|5524203 | OPA-containing protein | 6.35 | 225734 | 80 | 3 | 3.5 | no emPAI | 0±0 | n/a |
| 924 | gi\|5531821 | NPD001 | 10.29 | 15597 | 98 | 4 | 35 | no emPAI | 0±0 | n/a |

**Supplementary Table 1.** Altered phosphoproteins in antibody-depedent enhancement (ADE) of DENV2-infected U937 cells (cont.)

| **No.** | **NCBI ID** | **Protein** | **pI** | **MW (Da)** | **Identification score** | **No. of matched peptides** | **%cov** | **Mock (Mean±SD)** | **Treated (Mean±SD)** | **Ratio (Treated/Mock)** |
| --- | --- | --- | --- | --- | --- | --- | --- | --- | --- | --- |
| 925 | gi\|556192 | 51C protein | 9.08 | 126729 | 78 | 2 | 3.7 | 0±0 | no emPAI | n/a |
| 926 | gi\|55742736 | zinc finger protein 700 isoform 1 | 9.15 | 86176 | 70 | 2 | 7.7 | 0±0 | no emPAI | n/a |
| 927 | gi\|55749758 | disco-interacting protein 2 homolog B | 8.43 | 171382 | 97 | 4 | 5.7 | no emPAI | 0±0 | n/a |
| 928 | gi\|55770834 | centromere protein F | 5.06 | 357306 | 125 | 5 | 4.6 | 0±0 | no emPAI | n/a |
| 929 | gi\|559715 | HCAP-H | 5.01 | 83293 | 67 | 2 | 7.4 | no emPAI | 0±0 | n/a |
| 930 | gi\|56160428 | polymerase kappa isoform 2 | 8.45 | 55868 | 83 | 3 | 12.3 | no emPAI | 0±0 | n/a |
| 931 | gi\|563217 | NFX1 | 8.47 | 123068 | 92 | 4 | 7.8 | 0±0 | no emPAI | n/a |
| 932 | gi\|56417899 | ARF-binding protein 1 | 5.1 | 481605 | 113 | 4 | 3 | 0±0 | no emPAI | n/a |
| 933 | gi\|565324143 | FERM and PDZ domain-containing protein 3 | 8.44 | 199085 | 106 | 4 | 4.8 | 0±0 | no emPAI | n/a |
| 934 | gi\|56554357 | Chain A, Binary Structure Of Human Decr Solved By Semet Sad. | 8.78 | 32100 | 66 | 2 | 15.6 | 0±0 | no emPAI | n/a |
| 935 | gi\|565671697 | maestro heat-like repeat-containing protein family member 2A | 6.42 | 191089 | 66 | 2 | 3 | 0±0 | no emPAI | n/a |
| 936 | gi\|5669090 | API2-MLT fusion protein | 5.51 | 128656 | 102 | 4 | 7.4 | no emPAI | 0±0 | n/a |
| 937 | gi\|56792342 | AKAP9-BRAF fusion protein | 5.25 | 172057 | 145 | 6 | 6.4 | no emPAI | 0±0 | n/a |
| 938 | gi\|568245056 | AKAP350C | 4.87 | 362225 | 171 | 7 | 4.7 | no emPAI | 0±0 | n/a |
| 939 | gi\|568815724 | dihydropyrimidinase-related protein 1 isoform 4 | 6.41 | 61428 | 69 | 2 | 7.6 | no emPAI | 0±0 | n/a |
| 940 | gi\|5689551 | KIAA1107 protein | 5.93 | 140714 | 113 | 4 | 7 | no emPAI | 0±0 | n/a |
| 941 | gi\|5701717 | UDP-N-acetylglucosamine:alpha-1,3-D-mannoside beta-1,4-N-acetylglucosaminyltransferase IV-homologue | 8.03 | 56008 | 74 | 2 | 7.7 | 0±0 | no emPAI | n/a |
| 942 | gi\|5702306 | vault protein | 5.43 | 192550 | 66 | 2 | 5 | no emPAI | 0±0 | n/a |
| 943 | gi\|570359824 | Chain A, Crystal Structure Of The Human Cyclin G Associated Kinase (gak) | 6.51 | 37730 | 92 | 4 | 14.2 | 0±0 | no emPAI | n/a |

**Supplementary Table 1.** Altered phosphoproteins in antibody-depedent enhancement (ADE) of DENV2-infected U937 cells (cont.)

| **No.** | **NCBI ID** | **Protein** | **pI** | **MW (Da)** | **Identification score** | **No. of matched peptides** | **%cov** | **Mock (Mean±SD)** | **Treated (Mean±SD)** | **Ratio (Treated/Mock)** |
| --- | --- | --- | --- | --- | --- | --- | --- | --- | --- | --- |
| 944 | gi\|571026674 | transcription factor E4F1 isoform 2 | 8.64 | 70318 | 94 | 4 | 8.7 | 0±0 | no emPAI | n/a |
| 945 | gi\|57242774 | UPF0378 protein KIAA0100 precursor | 6.71 | 253539 | 145 | 5 | 5.7 | no emPAI | 0±0 | n/a |
| 946 | gi\|5729734 | protein DBF4 homolog A | 8.03 | 76810 | 123 | 5 | 12.8 | 0±0 | no emPAI | n/a |
| 947 | gi\|578796397 | PREDICTED: mucin-5AC, partial | 6.09 | 215191 | 98 | 4 | 5.7 | no emPAI | 0±0 | n/a |
| 948 | gi\|578796689 | PREDICTED: uncharacterized protein LOC144535 | 8.75 | 322309 | 101 | 4 | 3.1 | 0±0 | no emPAI | n/a |
| 949 | gi\|578797449 | PREDICTED: uncharacterized protein LOC102725322 | 9.82 | 49615 | 81 | 3 | 17.5 | no emPAI | 0±0 | n/a |
| 950 | gi\|578797738 | PREDICTED: uncharacterized protein LOC102723553 | 9.38 | 31167 | 66 | 2 | 10.8 | no emPAI | 0±0 | n/a |
| 951 | gi\|578797830 | PREDICTED: paternally-expressed gene 3 protein-like, partial | 4.78 | 112428 | 97 | 4 | 4.8 | 0±0 | no emPAI | n/a |
| 952 | gi\|578798066 | PREDICTED: myosin light chain kinase 2, skeletal/cardiac muscle-like | 8.87 | 61648 | 67 | 2 | 8.9 | no emPAI | 0±0 | n/a |
| 953 | gi\|578798573 | PREDICTED: sperm-associated antigen 17 isoform X4 | 5.9 | 151681 | 76 | 2 | 5 | 0±0 | no emPAI | n/a |
| 954 | gi\|578798804 | PREDICTED: microtubule-actin cross-linking factor 1 isoform X12 | 5.19 | 865433 | 150 | 7 | 3 | no emPAI | 0±0 | n/a |
| 955 | gi\|578799635 | PREDICTED: phosphatase and actin regulator 4 isoform X2 | 6.02 | 80922 | 78 | 2 | 6.7 | no emPAI | 0±0 | n/a |
| 956 | gi\|578800138 | PREDICTED: rootletin isoform X1 | 5.53 | 224093 | 66 | 2 | 3.4 | 0±0 | no emPAI | n/a |
| 957 | gi\|578800406 | PREDICTED: double-stranded RNA-specific adenosine deaminase isoform X1 | 8.81 | 137073 | 80 | 3 | 4.8 | no emPAI | 0±0 | n/a |
| 958 | gi\|578800920 | PREDICTED: hornerin isoform X1 | 9.88 | 213331 | 109 | 4 | 6.2 | 0±0 | no emPAI | n/a |
| 959 | gi\|578803490 | PREDICTED: striated muscle preferentially expressed protein kinase isoform X14 | 8.39 | 283537 | 148 | 7 | 5.5 | 0±0 | no emPAI | n/a |

**Supplementary Table 1.** Altered phosphoproteins in antibody-depedent enhancement (ADE) of DENV2-infected U937 cells (cont.)

| **No.** | **NCBI ID** | **Protein** | **pI** | **MW (Da)** | **Identification score** | **No. of matched peptides** | **%cov** | **Mock (Mean±SD)** | **Treated (Mean±SD)** | **Ratio (Treated/Mock)** |
| --- | --- | --- | --- | --- | --- | --- | --- | --- | --- | --- |
| 960 | gi\|578804363 | PREDICTED: unconventional myosin-VIIb isoform X1 | 8.92 | 244294 | 107 | 4 | 5.9 | 0±0 | no emPAI | n/a |
| 961 | gi\|578804672 | PREDICTED: ankyrin repeat domain-containing protein 36B isoform X11 | 9 | 149847 | 67 | 2 | 7.1 | no emPAI | 0±0 | n/a |
| 962 | gi\|578804834 | PREDICTED: titin isoform X6 | 6.04 | 3773133 | 297 | 10 | 3.5 | no emPAI | 0±0 | n/a |
| 963 | gi\|578805110 | PREDICTED: coiled-coil domain-containing protein 74A isoform X5 | 11.12 | 36162 | 73 | 2 | 18 | no emPAI | 0±0 | n/a |
| 964 | gi\|578805156 | PREDICTED: leucine-rich repeat flightless-interacting protein 1 isoform X35 | 4.69 | 105504 | 111 | 4 | 11.5 | no emPAI | 0±0 | n/a |
| 965 | gi\|578805890 | PREDICTED: inactive phospholipase C-like protein 2 isoform X1 | 6 | 115970 | 83 | 3 | 6 | no emPAI | 0±0 | n/a |
| 966 | gi\|578806857 | PREDICTED: xylulose kinase isoform X5 | 5.77 | 43154 | 77 | 3 | 13 | 0±0 | no emPAI | n/a |
| 967 | gi\|578807038 | PREDICTED: cell surface glycoprotein CD200 receptor 1 isoform X1 | 9 | 35273 | 66 | 2 | 6 | no emPAI | 0±0 | n/a |
| 968 | gi\|578808301 | PREDICTED: protein FAM193A isoform X3 | 5.94 | 160835 | 100 | 4 | 3.5 | no emPAI | 0±0 | n/a |
| 969 | gi\|578808689 | PREDICTED: centrosomal protein of 135 kDa isoform X2 | 5.82 | 132037 | 86 | 3 | 5.2 | 0±0 | no emPAI | n/a |
| 970 | gi\|578809088 | PREDICTED: coiled-coil domain-containing protein 158 isoform X6 | 6.34 | 128402 | 74 | 2 | 8 | 0±0 | no emPAI | n/a |
| 971 | gi\|578809111 | PREDICTED: plasma kallikrein isoform X2 | 8.63 | 67103 | 70 | 2 | 6.2 | 0±0 | no emPAI | n/a |
| 972 | gi\|578809489 | PREDICTED: sorbin and SH3 domain-containing protein 2 isoform X29 | 6.18 | 146779 | 117 | 5 | 6.9 | no emPAI | 0±0 | n/a |
| 973 | gi\|578810328 | PREDICTED: microtubule-associated serine/threonine-protein kinase 4 isoform X2 | 8.9 | 287102 | 140 | 6 | 5.7 | 0±0 | no emPAI | n/a |
| 974 | gi\|578810583 | PREDICTED: calpastatin isoform X6 | 5.1 | 82387 | 130 | 6 | 13.9 | no emPAI | 0±0 | n/a |

**Supplementary Table 1.** Altered phosphoproteins in antibody-depedent enhancement (ADE) of DENV2-infected U937 cells (cont.)

| **No.** | **NCBI ID** | **Protein** | **pI** | **MW (Da)** | **Identification score** | **No. of matched peptides** | **%cov** | **Mock (Mean±SD)** | **Treated (Mean±SD)** | **Ratio (Treated/Mock)** |
| --- | --- | --- | --- | --- | --- | --- | --- | --- | --- | --- |
| 975 | gi\|578812976 | PREDICTED: androglobin isoform X1 | 8.49 | 189580 | 86 | 3 | 4.6 | 0±0 | no emPAI | n/a |
| 976 | gi\|578813410 | PREDICTED: amphiphysin isoform X1 | 4.45 | 134798 | 114 | 4 | 5.2 | 0±0 | no emPAI | n/a |
| 977 | gi\|578813686 | PREDICTED: trinucleotide repeat-containing gene 18 protein isoform X2 | 9.01 | 232055 | 128 | 6 | 7.4 | no emPAI | 0±0 | n/a |
| 978 | gi\|578814313 | PREDICTED: neuronal cell adhesion molecule isoform X24 | 5.35 | 133417 | 80 | 3 | 5 | 0±0 | no emPAI | n/a |
| 979 | gi\|578816138 | PREDICTED: maestro heat-like repeat-containing protein family member 1 isoform X1 | 6.52 | 181676 | 82 | 3 | 3.6 | no emPAI | 0±0 | n/a |
| 980 | gi\|578816553 | PREDICTED: centlein isoform X2 | 8.51 | 155582 | 70 | 2 | 6.3 | no emPAI | 0±0 | n/a |
| 981 | gi\|578817809 | PREDICTED: spectrin alpha chain, non-erythrocytic 1 isoform X7 | 5.22 | 285172 | 89 | 3 | 3.6 | no emPAI | 0±0 | n/a |
| 982 | gi\|578818464 | PREDICTED: sickle tail protein homolog isoform X27 | 6.67 | 214106 | 119 | 5 | 6.7 | no emPAI | 0±0 | n/a |
| 983 | gi\|578818488 | PREDICTED: partitioning defective 3 homolog isoform X17 | 6.39 | 126327 | 117 | 5 | 5.6 | 0±0 | no emPAI | n/a |
| 984 | gi\|578818573 | PREDICTED: uncharacterized protein C10orf68 isoform X5 | 7.16 | 157352 | 77 | 3 | 4.3 | no emPAI | 0±0 | n/a |
| 985 | gi\|578818788 | PREDICTED: sorbin and SH3 domain-containing protein 1 isoform X52 | 7.19 | 168453 | 70 | 2 | 4.6 | no emPAI | 0±0 | n/a |
| 986 | gi\|578819312 | PREDICTED: ankyrin-3 isoform X14 | 5.73 | 387388 | 144 | 5 | 5.6 | no emPAI | 0±0 | n/a |
| 987 | gi\|578819342 | PREDICTED: ankyrin-3 isoform X29 | 7.84 | 205953 | 112 | 4 | 3.7 | no emPAI | 0±0 | n/a |
| 988 | gi\|578819440 | PREDICTED: actin-binding LIM protein 1 isoform X19 | 8.77 | 82746 | 82 | 3 | 11.7 | no emPAI | 0±0 | n/a |
| 989 | gi\|578821001 | PREDICTED: mucin-5AC | 6.56 | 520340 | 96 | 4 | 1.6 | 0±0 | no emPAI | n/a |
| 990 | gi\|578821432 | PREDICTED: unconventional myosin-VIIa isoform X3 | 8.86 | 255235 | 86 | 3 | 3.9 | 0±0 | no emPAI | n/a |

**Supplementary Table 1.** Altered phosphoproteins in antibody-depedent enhancement (ADE) of DENV2-infected U937 cells (cont.)

| **No.** | **NCBI ID** | **Protein** | **pI** | **MW (Da)** | **Identification score** | **No. of matched peptides** | **%cov** | **Mock (Mean±SD)** | **Treated (Mean±SD)** | **Ratio (Treated/Mock)** |
| --- | --- | --- | --- | --- | --- | --- | --- | --- | --- | --- |
| 991 | gi\|578821730 | PREDICTED: neuroblast differentiation-associated protein AHNAK isoform X9 | 5.96 | 423528 | 169 | 8 | 4.1 | 0±0 | no emPAI | n/a |
| 992 | gi\|578821826 | PREDICTED: 182 kDa tankyrase-1-binding protein isoform X4 | 4.78 | 111431 | 72 | 2 | 6.6 | 0±0 | no emPAI | n/a |
| 993 | gi\|578822065 | PREDICTED: exophilin-5 isoform X2 | 6.81 | 204383 | 124 | 5 | 5.9 | DIV/0 | no emPAI | n/a |
| 994 | gi\|578822277 | PREDICTED: serine/threonine-protein phosphatase 2A 65 kDa regulatory subunit A beta isoform isoform X1 | 5.53 | 84734 | 80 | 3 | 7.8 | no emPAI | 0±0 | n/a |
| 995 | gi\|578824032 | PREDICTED: disco-interacting protein 2 homolog B isoform X4 | 8.39 | 158512 | 139 | 6 | 6.1 | DIV/0 | no emPAI | n/a |
| 996 | gi\|578824414 | PREDICTED: neuron navigator 3 isoform X4 | 8.78 | 247060 | 115 | 4 | 6.9 | no emPAI | 0±0 | n/a |
| 997 | gi\|578824605 | PREDICTED: uncharacterized protein LOC144535 | 8.73 | 322366 | 122 | 5 | 5 | no emPAI | 0±0 | n/a |
| 998 | gi\|578824784 | PREDICTED: probable E3 ubiquitin-protein ligase MYCBP2 isoform X3 | 6.73 | 526204 | 102 | 3 | 3.2 | 0±0 | no emPAI | n/a |
| 999 | gi\|578824800 | PREDICTED: probable E3 ubiquitin-protein ligase MYCBP2 isoform X11 | 6.7 | 515217 | 194 | 9 | 5.5 | DIV/0 | no emPAI | n/a |
| 1000 | gi\|578824845 | PREDICTED: neurobeachin isoform X6 | 5.74 | 212636 | 76 | 2 | 2.7 | no emPAI | 0±0 | n/a |
| 1001 | gi\|578824866 | PREDICTED: LIM domain only protein 7 isoform X27 | 7.8 | 208695 | 101 | 4 | 6.7 | no emPAI | 0±0 | n/a |
| 1002 | gi\|578827007 | PREDICTED: unconventional myosin-IXa isoform X4 | 8.98 | 191285 | 162 | 7 | 6.8 | no emPAI | 0±0 | n/a |
| 1003 | gi\|578827259 | PREDICTED: ras-specific guanine nucleotide-releasing factor 1 isoform X2 | 8.24 | 140704 | 69 | 2 | 6.1 | no emPAI | 0±0 | n/a |
| 1004 | gi\|578827487 | PREDICTED: talin-2 isoform X13 | 5.46 | 268860 | 185 | 6 | 7 | no emPAI | 0±0 | n/a |
| 1005 | gi\|578828140 | PREDICTED: periplakin isoform X1 | 5.38 | 205526 | 105 | 4 | 4.2 | no emPAI | 0±0 | n/a |

**Supplementary Table 1.** Altered phosphoproteins in antibody-depedent enhancement (ADE) of DENV2-infected U937 cells (cont.)

| **No.** | **NCBI ID** | **Protein** | **pI** | **MW (Da)** | **Identification score** | **No. of matched peptides** | **%cov** | **Mock (Mean±SD)** | **Treated (Mean±SD)** | **Ratio (Treated/Mock)** |
| --- | --- | --- | --- | --- | --- | --- | --- | --- | --- | --- |
| 1006 | gi\|578828326 | PREDICTED: voltage-dependent T-type calcium channel subunit alpha-1H isoform X6 | 8.56 | 178427 | 97 | 4 | 5.3 | no emPAI | 0±0 | n/a |
| 1007 | gi\|578828592 | PREDICTED: dynein heavy chain 3, axonemal isoform X1 | 6 | 465974 | 92 | 4 | 2.9 | no emPAI | 0±0 | n/a |
| 1008 | gi\|578829655 | PREDICTED: chromodomain-helicase-DNA-binding protein 3 isoform X10 | 8.37 | 229675 | 82 | 2 | 3.6 | 0±0 | no emPAI | n/a |
| 1009 | gi\|578829750 | PREDICTED: dynein heavy chain 9, axonemal isoform X1 | 5.72 | 441462 | 133 | 4 | 3.4 | no emPAI | 0±0 | n/a |
| 1010 | gi\|578830959 | PREDICTED: protein AF-17 isoform X1 | 8.84 | 114976 | 83 | 3 | 5.1 | 0±0 | no emPAI | n/a |
| 1011 | gi\|578831186 | PREDICTED: E3 ubiquitin-protein ligase RNF213 isoform X1 | 6 | 596107 | 112 | 4 | 3.5 | 0±0 | no emPAI | n/a |
| 1012 | gi\|578832667 | PREDICTED: transcription factor 4 isoform X31 | 8.84 | 75644 | 105 | 4 | 10.6 | no emPAI | 0±0 | n/a |
| 1013 | gi\|578832927 | PREDICTED: zinc finger protein 266 isoform X5 | 8.57 | 69805 | 77 | 3 | 8.6 | 0±0 | no emPAI | n/a |
| 1014 | gi\|578833555 | PREDICTED: perilipin-4 isoform X6 | 8.85 | 123226 | 92 | 4 | 8.9 | no emPAI | 0±0 | n/a |
| 1015 | gi\|578835719 | PREDICTED: receptor-type tyrosine-protein phosphatase T isoform X3 | 6.84 | 160682 | 73 | 2 | 3 | no emPAI | 0±0 | n/a |
| 1016 | gi\|578835897 | PREDICTED: nuclear receptor coactivator 6 isoform X9 | 9.24 | 209756 | 82 | 3 | 1.5 | 0±0 | no emPAI | n/a |
| 1017 | gi\|578837815 | PREDICTED: BEN domain-containing protein 2 isoform X1 | 8.44 | 48059 | 109 | 4 | 18.9 | no emPAI | 0±0 | n/a |
| 1018 | gi\|578845984 | PREDICTED: SAGA-associated factor 29 homolog isoform X2 | 10.39 | 35280 | 74 | 2 | 12.8 | no emPAI | 0±0 | n/a |
| 1019 | gi\|578846521 | PREDICTED: uncharacterized protein LOC102723805 isoform X8 | 9.56 | 53398 | 111 | 4 | 12.8 | no emPAI | 0±0 | n/a |
| 1020 | gi\|57997475 | hypothetical protein | 7.93 | 101806 | 65 | 2 | 3.7 | 0±0 | no emPAI | n/a |

**Supplementary Table 1.** Altered phosphoproteins in antibody-depedent enhancement (ADE) of DENV2-infected U937 cells (cont.)

| **No.** | **NCBI ID** | **Protein** | **pI** | **MW (Da)** | **Identification score** | **No. of matched peptides** | **%cov** | **Mock (Mean±SD)** | **Treated (Mean±SD)** | **Ratio (Treated/Mock)** |
| --- | --- | --- | --- | --- | --- | --- | --- | --- | --- | --- |
| 1021 | gi\|5802821 | Gag-Pro-Pol protein | 8.98 | 208865 | 76 | 2 | 3.1 | 0±0 | no emPAI | n/a |
| 1022 | gi\|5803121 | protein disulfide-isomerase A5 precursor | 8.08 | 59556 | 67 | 2 | 6.6 | 0±0 | no emPAI | n/a |
| 1023 | gi\|58197558 | tudor domain-containing protein 6 isoform 1 | 5.14 | 236367 | 70 | 2 | 2.8 | no emPAI | 0±0 | n/a |
| 1024 | gi\|5821145 | RNA binding protein | 12.3 | 106400 | 141 | 6 | 11.6 | 0±0 | no emPAI | n/a |
| 1025 | gi\|58257676 | KIAA1032 protein | 5.21 | 192745 | 103 | 4 | 5.2 | 0±0 | no emPAI | n/a |
| 1026 | gi\|58257686 | KIAA1277 protein | 9.04 | 117247 | 93 | 4 | 4.3 | 0±0 | no emPAI | n/a |
| 1027 | gi\|58257688 | KIAA1426 protein | 7.05 | 113996 | 75 | 2 | 7.4 | no emPAI | 0±0 | n/a |
| 1028 | gi\|58257700 | KIAA1558 protein | 4.55 | 98739 | 78 | 2 | 4.5 | no emPAI | 0±0 | n/a |
| 1029 | gi\|58331187 | T-lymphoma invasion and metastasis-inducing protein 2 isoform a | 6.8 | 189995 | 131 | 6 | 7.6 | 0±0 | no emPAI | n/a |
| 1030 | gi\|585724702 | lysyl oxidase-like 2 delta e13 | 6.26 | 81144 | 68 | 2 | 2.1 | 0±0 | no emPAI | n/a |
| 1031 | gi\|586476412 | PREDICTED: twisted gastrulation protein homolog 1 [Chrysochloris asiatica] | 5.34 | 16798 | 77 | 3 | 30.4 | no emPAI | 0±0 | n/a |
| 1032 | gi\|5869878 | apoptotic protease activating factor 1 | 6.03 | 136987 | 100 | 4 | 8.5 | no emPAI | 0±0 | n/a |
| 1033 | gi\|5869884 | apoptotic protease activating factor 1 | 6.01 | 136994 | 90 | 3 | 8 | no emPAI | 0±0 | n/a |
| 1034 | gi\|5870866 | TATA element modulatory factor | 4.95 | 123096 | 73 | 2 | 5.9 | 0±0 | no emPAI | n/a |
| 1035 | gi\|591320686 | PREDICTED: ADP-ribosylation factor-like protein 8A isoform X2 [Panthera tigris altaica] | 5.32 | 10480 | 71 | 2 | 38 | no emPAI | 0±0 | n/a |
| 1036 | gi\|6005757 | FACT complex subunit SPT16 | 5.5 | 119838 | 79 | 3 | 6.1 | no emPAI | 0±0 | n/a |
| 1037 | gi\|6005970 | zinc finger protein 175 | 8.98 | 81557 | 72 | 2 | 4.6 | no emPAI | 0±0 | n/a |
| 1038 | gi\|6006515 | spliceosomal protein SAP 130 | 5.15 | 135507 | 69 | 2 | 4.7 | no emPAI | 0±0 | n/a |
| 1039 | gi\|60097902 | filaggrin | 9.24 | 434922 | 151 | 7 | 4.9 | 0±0 | no emPAI | n/a |
| 1040 | gi\|6010217 | NOV/plexin-A1 protein | 6.56 | 194686 | 72 | 2 | 3.6 | no emPAI | 0±0 | n/a |

**Supplementary Table 1.** Altered phosphoproteins in antibody-depedent enhancement (ADE) of DENV2-infected U937 cells (cont.)

| **No.** | **NCBI ID** | **Protein** | **pI** | **MW (Da)** | **Identification score** | **No. of matched peptides** | **%cov** | **Mock (Mean±SD)** | **Treated (Mean±SD)** | **Ratio (Treated/Mock)** |
| --- | --- | --- | --- | --- | --- | --- | --- | --- | --- | --- |
| 1041 | gi\|608785732 | major vault protein isoform E | 10.67 | 13698 | 68 | 2 | 21.1 | 0±0 | no emPAI | n/a |
| 1042 | gi\|612407777 | zinc finger protein 285 isoform c | 8.85 | 50010 | 93 | 4 | 14.3 | 0±0 | no emPAI | n/a |
| 1043 | gi\|61679987 | Chain A, Crystal Structure Of The TollINTERLEUKIN-1 Receptor (Tir) Domain Of Human Il-1rapl | 6.37 | 18853 | 69 | 2 | 18.2 | no emPAI | 0±0 | n/a |
| 1044 | gi\|62087248 | Zinc finger protein 236 variant | 8.81 | 152549 | 77 | 3 | 5 | no emPAI | 0±0 | n/a |
| 1045 | gi\|62088204 | DNA-repair protein complementing XP-G cells variant | 6.03 | 181337 | 91 | 3 | 4.2 | 0±0 | no emPAI | n/a |
| 1046 | gi\|62088246 | protein tyrosine phosphatase, receptor type, sigma isoform 3 precursor variant | 6.61 | 174545 | 66 | 2 | 4.6 | no emPAI | 0±0 | n/a |
| 1047 | gi\|62088700 | polymerase (DNA directed), delta 1, catalytic subunit 125kDa variant | 7.68 | 111788 | 66 | 2 | 4.8 | no emPAI | 0±0 | n/a |
| 1048 | gi\|62148934 | BNIP2 motif containing molecule at the carboxyl tarminal region 1 | 4.31 | 298883 | 75 | 2 | 2.9 | no emPAI | 0±0 | n/a |
| 1049 | gi\|62526543 | vanilloid receptor variant TRPV1b | 8.05 | 88009 | 72 | 2 | 7.4 | 0±0 | no emPAI | n/a |
| 1050 | gi\|62550738 | calcium/calmodulin-dependent protein kinase II delta | 7.62 | 55331 | 71 | 2 | 11.7 | 0±0 | no emPAI | n/a |
| 1051 | gi\|6273778 | trabeculin-alpha | 5.26 | 613652 | 141 | 6 | 3.1 | no emPAI | 0±0 | n/a |
| 1052 | gi\|62860046 | DNA annealing helicase and endonuclease ZRANB3 isoform 1 | 8.74 | 123170 | 73 | 2 | 6.6 | no emPAI | 0±0 | n/a |
| 1053 | gi\|62871110 | immunoglobulin alpha heavy chain variable region | 8.81 | 17240 | 132 | 5 | 18.8 | no emPAI | 0±0 | n/a |
| 1054 | gi\|62897687 | trans-golgi network protein 2 variant | 5.39 | 45909 | 77 | 3 | 18.8 | no emPAI | 0±0 | n/a |
| 1055 | gi\|62898077 | tropomyosin 2 (beta) isoform 2 variant | 4.63 | 32943 | 67 | 2 | 14.1 | no emPAI | 0±0 | n/a |
| 1056 | gi\|62898956 | pinin, desmosome associated protein variant | 6.71 | 81535 | 74 | 2 | 9.1 | no emPAI | 0±0 | n/a |
| 1057 | gi\|63102229 | Zinc finger protein 592 | 8.1 | 137468 | 87 | 2 | 3.8 | 0±0 | no emPAI | n/a |
| 1058 | gi\|6331407 | KIAA1288 protein | 8.26 | 138448 | 81 | 3 | 5.9 | 0±0 | no emPAI | n/a |

**Supplementary Table 1.** Altered phosphoproteins in antibody-depedent enhancement (ADE) of DENV2-infected U937 cells (cont.)

| **No.** | **NCBI ID** | **Protein** | **pI** | **MW (Da)** | **Identification score** | **No. of matched peptides** | **%cov** | **Mock (Mean±SD)** | **Treated (Mean±SD)** | **Ratio (Treated/Mock)** |
| --- | --- | --- | --- | --- | --- | --- | --- | --- | --- | --- |
| 1059 | gi\|635172847 | kelch-like protein 8 isoform 2 | 6.22 | 60242 | 88 | 3 | 12.7 | no emPAI | 0±0 | n/a |
| 1060 | gi\|635575426 | Chain A, Crystal Structure Of Sulfamidase | 6.69 | 57733 | 65 | 2 | 4.5 | 0±0 | no emPAI | n/a |
| 1061 | gi\|642945631 | titin isoform IC | 6.05 | 3992165 | 316 | 13 | 3.1 | 0±0 | no emPAI | n/a |
| 1062 | gi\|65301139 | probable phospholipid-transporting ATPase IIA | 8 | 118506 | 73 | 2 | 4.5 | 0±0 | no emPAI | n/a |
| 1063 | gi\|6633953 | KIAA0097 protein | 8.11 | 225893 | 76 | 2 | 3.8 | 0±0 | 0±0 | n/a |
| 1064 | gi\|66346695 | fibrillin-2 precursor | 4.73 | 314558 | 83 | 3 | 6.9 | no emPAI | 0±0 | n/a |
| 1065 | gi\|6649242 | splicing coactivator subunit SRm300 | 11.96 | 251817 | 145 | 6 | 7.4 | 0±0 | 0±0 | n/a |
| 1066 | gi\|6649886 | Ig heavy chain | 10.09 | 16045 | 83 | 3 | 26.8 | no emPAI | 0±0 | n/a |
| 1067 | gi\|6683645 | KIAA0347 protein | 6.13 | 139340 | 123 | 5 | 9.4 | no emPAI | 0±0 | n/a |
| 1068 | gi\|6683749 | hqp0376 protein | 8.17 | 12510 | 69 | 2 | 36.1 | no emPAI | 0±0 | n/a |
| 1069 | gi\|672886600 | Chain A, Crystal Structure Of A Functionally Uncharacterized Domain Of E3 Ubiquitin Ligase Shprh | 6.25 | 48208 | 147 | 7 | 18.6 | no emPAI | 0±0 | n/a |
| 1070 | gi\|675724255 | PREDICTED: protein kinase C beta type isoform X2 [Pan paniscus] | 5.68 | 62381 | 86 | 3 | 6.5 | 0±0 | no emPAI | n/a |
| 1071 | gi\|675733893 | PREDICTED: E3 ubiquitin-protein ligase MIB1 [Pan paniscus] | 6.49 | 101085 | 73 | 2 | 8.2 | no emPAI | 0±0 | n/a |
| 1072 | gi\|675765492 | PREDICTED: grainyhead-like protein 2 homolog isoform X2 [Pan paniscus] | 6.05 | 69278 | 74 | 2 | 9 | 0±0 | no emPAI | n/a |
| 1073 | gi\|6807904 | hypothetical protein | 8.59 | 23934 | 85 | 3 | 18.2 | no emPAI | 0±0 | n/a |
| 1074 | gi\|683437139 | Chain D, Crystal Structure Of Heterotetrameric Human Ketoacyl Reductase Complexed With Nad And Nadp | 6.09 | 26981 | 90 | 3 | 32.6 | 0±0 | no emPAI | n/a |
| 1075 | gi\|6841336 | HSPC049 | 8.55 | 82899 | 69 | 2 | 10 | 0±0 | no emPAI | n/a |

**Supplementary Table 1.** Altered phosphoproteins in antibody-depedent enhancement (ADE) of DENV2-infected U937 cells (cont.)

| **No.** | **NCBI ID** | **Protein** | **pI** | **MW (Da)** | **Identification score** | **No. of matched peptides** | **%cov** | **Mock (Mean±SD)** | **Treated (Mean±SD)** | **Ratio (Treated/Mock)** |
| --- | --- | --- | --- | --- | --- | --- | --- | --- | --- | --- |
| 1076 | gi\|68533053 | JARID1A variant protein | 6.14 | 196230 | 67 | 2 | 3 | no emPAI | 0±0 | n/a |
| 1077 | gi\|68533123 | C6orf21 variant protein | 6.67 | 163276 | 67 | 2 | 3.5 | 0±0 | no emPAI | n/a |
| 1078 | gi\|6939732 | transcription factor Elongin A2 | 9.73 | 83873 | 81 | 3 | 8 | no emPAI | 0±0 | n/a |
| 1079 | gi\|7018409 | hypothetical protein | 5.34 | 84825 | 66 | 2 | 6.1 | no emPAI | 0±0 | n/a |
| 1080 | gi\|704000369 | RecName: Full=Basic helix-loop-helix and HMG box domain-containing protein 1 | 9.1 | 70154 | 71 | 2 | 11.3 | 0±0 | no emPAI | n/a |
| 1081 | gi\|71051467 | IQUB protein | 6.03 | 69339 | 88 | 3 | 10.2 | 0±0 | no emPAI | n/a |
| 1082 | gi\|71051553 | ZNF557 protein, partial | 9.36 | 46150 | 74 | 2 | 11.1 | no emPAI | 0±0 | n/a |
| 1083 | gi\|71297475 | ST7L protein | 8.42 | 36682 | 71 | 2 | 15.3 | 0±0 | no emPAI | n/a |
| 1084 | gi\|71297481 | PARP8 protein | 9.89 | 55098 | 68 | 2 | 7.5 | 0±0 | no emPAI | n/a |
| 1085 | gi\|71891683 | KIAA0774 protein | 6.24 | 151264 | 95 | 4 | 5.7 | no emPAI | 0±0 | n/a |
| 1086 | gi\|71891685 | KIAA0829 protein | 5.71 | 141472 | 105 | 4 | 10.8 | no emPAI | 0±0 | n/a |
| 1087 | gi\|71891705 | KIAA1664 protein | 6.1 | 101474 | 77 | 3 | 10.1 | no emPAI | 0±0 | n/a |
| 1088 | gi\|71891711 | KIAA1858 protein | 8.24 | 258549 | 69 | 2 | 3.1 | 0±0 | no emPAI | n/a |
| 1089 | gi\|7242977 | KIAA1311 protein | 9.42 | 98234 | 90 | 3 | 5.5 | no emPAI | 0±0 | n/a |
| 1090 | gi\|7243015 | KIAA1317 protein | 8.6 | 49905 | 81 | 3 | 4.4 | 0±0 | no emPAI | n/a |
| 1091 | gi\|7243105 | KIAA1362 protein | 6.06 | 79902 | 76 | 2 | 4.9 | 0±0 | no emPAI | n/a |
| 1092 | gi\|7417372 | intracellular hyaluronan-binding protein | 7.02 | 45824 | 68 | 2 | 11.9 | no emPAI | 0±0 | n/a |
| 1093 | gi\|7427519 | DNA replication licensing factor MCM6 | 5.29 | 92831 | 69 | 2 | 2.8 | no emPAI | 0±0 | n/a |
| 1094 | gi\|748983076 | mucin-5AC precursor | 6.71 | 585199 | 110 | 4 | 2.5 | no emPAI | 0±0 | n/a |
| 1095 | gi\|7542537 | mixed lineage kinase ZAK | 7.92 | 91207 | 70 | 2 | 7.8 | 0±0 | no emPAI | n/a |
| 1096 | gi\|756398302 | G patch domain-containing protein 8 isoform 3 | 8.95 | 155100 | 142 | 6 | 11 | no emPAI | 0±0 | n/a |

**Supplementary Table 1.** Altered phosphoproteins in antibody-depedent enhancement (ADE) of DENV2-infected U937 cells (cont.)

| **No.** | **NCBI ID** | **Protein** | | **pI** | **MW (Da)** | **Identification score** | **No. of matched peptides** | **%cov** | **Mock (Mean±SD)** | **Treated (Mean±SD)** | **Ratio (Treated/Mock)** |
| --- | --- | --- | --- | --- | --- | --- | --- | --- | --- | --- | --- |
| 1097 | gi\|7573074 | | immunoglobulin lambda light chain variable region | 9.07 | 7147 | 77 | 3 | 44.4 | 0±0 | no emPAI | n/a |
| 1098 | gi\|76150613 | dedicator of cytokinesis 8 | | 6.43 | 238409 | 85 | 3 | 3.2 | no emPAI | 0±0 | n/a |
| 1099 | gi\|7638038 | beta V spectrin | | 6.23 | 416579 | 123 | 6 | 2.6 | 0±0 | no emPAI | n/a |
| 1100 | gi\|7656861 | disintegrin and metalloproteinase domain-containing protein 18 isoform 1 preproprotein | | 7.16 | 82802 | 65 | 2 | 8.1 | no emPAI | 0±0 | n/a |
| 1101 | gi\|7656967 | cadherin EGF LAG seven-pass G-type receptor 1 precursor | | 5.59 | 329278 | 75 | 2 | 3.8 | 0±0 | no emPAI | n/a |
| 1102 | gi\|7657619 | sushi repeat-containing protein SRPX2 precursor | | 7.05 | 52938 | 71 | 2 | 9.7 | 0±0 | no emPAI | n/a |
| 1103 | gi\|7662394 | rab11 family-interacting protein 2 | | 9.33 | 58243 | 68 | 2 | 10 | no emPAI | 0±0 | n/a |
| 1104 | gi\|7688705 | AD-012 protein | | 6.99 | 60671 | 68 | 2 | 7.2 | 0±0 | no emPAI | n/a |
| 1105 | gi\|7739725 | ASH1 | | 9.46 | 332560 | 150 | 7 | 5 | 0±0 | no emPAI | n/a |
| 1106 | gi\|77819840 | rhabdomyosarcoma antigen MU-RMS-40.12 | | 6.24 | 55605 | 81 | 3 | 13.3 | 0±0 | no emPAI | n/a |
| 1107 | gi\|7799418 | ELG protein | | 6.8 | 38896 | 78 | 2 | 9.1 | 0±0 | no emPAI | n/a |
| 1108 | gi\|77997595 | c-myc intron-binding protein 1 | | 6.45 | 268634 | 114 | 4 | 4.5 | 0±0 | no emPAI | n/a |
| 1109 | gi\|790819 | polycystic kidney disease-associated protein | | 6.45 | 391939 | 109 | 4 | 3.7 | no emPAI | 0±0 | n/a |
| 1110 | gi\|7959207 | KIAA1473 protein | | 9.33 | 65909 | 101 | 4 | 12.2 | no emPAI | 0±0 | n/a |
| 1111 | gi\|7959253 | KIAA1496 protein | | 5.82 | 100681 | 69 | 2 | 3.8 | no emPAI | 0±0 | n/a |
| 1112 | gi\|799335 | autosomal dominant polycystic kidney disease protein 1 | | 6.26 | 462359 | 115 | 4 | 3 | no emPAI | 0±0 | n/a |
| 1113 | gi\|82654947 | RecName: Full=Keratin, type II cytoskeletal 4; AltName: Full=Cytokeratin-4; Short=CK-4; AltName: Full=Keratin-4; Short=K4; AltName: Full=Type-II keratin Kb4 | | 6.25 | 57250 | 75 | 2 | 8.2 | no emPAI | 0±0 | n/a |

**Supplementary Table 1.** Altered phosphoproteins in antibody-depedent enhancement (ADE) of DENV2-infected U937 cells (cont.)

| **No.** | **NCBI ID** | **Protein** | **pI** | **MW (Da)** | **Identification score** | **No. of matched peptides** | **%cov** | **Mock (Mean±SD)** | **Treated (Mean±SD)** | **Ratio (Treated/Mock)** |
| --- | --- | --- | --- | --- | --- | --- | --- | --- | --- | --- |
| 1114 | gi\|82830440 | Fanconi anemia group I protein isoform 2 | 6.33 | 142479 | 114 | 4 | 7.5 | 0±0 | 0±0 | n/a |
| 1115 | gi\|836683 | metalloprotease/disintegrin-like protein major transcript form | 6.63 | 72111 | 69 | 2 | 6.9 | no emPAI | 0±0 | n/a |
| 1116 | gi\|83715968 | coiled-coil domain-containing protein 73 | 5.42 | 124077 | 84 | 3 | 8.2 | no emPAI | 0±0 | n/a |
| 1117 | gi\|8489093 | winged helix/forkhead transcription factor | 9.58 | 41459 | 66 | 2 | 6.2 | no emPAI | 0±0 | n/a |
| 1118 | gi\|849083 | acetyl-CoA carboxylase | 6.1 | 264872 | 71 | 2 | 2.1 | 0±0 | no emPAI | n/a |
| 1119 | gi\|85726510 | ZNF493 protein, partial | 9.46 | 78988 | 82 | 3 | 10 | no emPAI | 0±0 | n/a |
| 1120 | gi\|87299628 | biorientation of chromosomes in cell division protein 1-like 1 | 5 | 330266 | 102 | 4 | 2.9 | 0±0 | no emPAI | n/a |
| 1121 | gi\|89130381 | CAP-GLY domain containing linker protein 1 | 5.32 | 156671 | 103 | 4 | 5.6 | no emPAI | 0±0 | n/a |
| 1122 | gi\|8922077 | double-stranded RNA-specific editase B2 | 10.19 | 80571 | 79 | 3 | 9.2 | no emPAI | 0±0 | n/a |
| 1123 | gi\|8923448 | 39S ribosomal protein L16, mitochondrial | 10.13 | 28431 | 66 | 2 | 9.6 | 0±0 | no emPAI | n/a |
| 1124 | gi\|89363020 | NF-X1-type zinc finger protein NFXL1 | 8.85 | 101270 | 73 | 2 | 10.9 | 0±0 | no emPAI | n/a |
| 1125 | gi\|9247066 | cone photoreceptor cyclic nucleotide-gated channel beta subunit | 8.08 | 92192 | 70 | 2 | 6.8 | 0±0 | no emPAI | n/a |
| 1126 | gi\|9255863 | chromokinesin | 6.29 | 125863 | 77 | 3 | 6 | 0±0 | no emPAI | n/a |
| 1127 | gi\|927596 | prepromultimerin | 8.15 | 137985 | 95 | 4 | 8.6 | no emPAI | 0±0 | n/a |
| 1128 | gi\|9279803 | transcription factor BMAL2 | 7.25 | 61748 | 76 | 2 | 7.6 | 0±0 | no emPAI | n/a |
| 1129 | gi\|987661 | transcriptional activator hSNF2a | 6.84 | 179222 | 69 | 2 | 2.5 | no emPAI | 0±0 | n/a |
| 1130 | gi\|99031624 | Chain A, Crystal Structure Of Homo Sapien Glycerol-3-Phosphate Dehydrogenase 1 | 5.81 | 37774 | 107 | 4 | 10.7 | no emPAI | 0±0 | n/a |
| 1131 | gi\|9966764 | acyl-protein thioesterase 2 | 6.75 | 24721 | 66 | 2 | 19 | no emPAI | 0±0 | n/a |

NCBI = National center for Biotechnology Information. %Cov. = %Sequence covage [(number of the mathched residues/total number of residues in the entire sequence) x 100%]. DIV/0 = Divide by zero. emPAI = Exponentially modified protein abundance index. n/a = not available.
