# Supplementary material for: Protein Disulfide Isomerase Inhibitor Suppresses Viral Replication and Production during Antibody-Dependent Enhancement of Dengue Virus Infection in Human Monocytic Cells
Source: Viruses. 2019 Feb 13;11(2):155. doi: 10.3390/v11020155 (PMC6410196; doi:10.3390/v11020155)
Supplement: Supplementary file 1 [file viruses-11-00155-s001.zip › Supplementary Table 2 Nov 2018.docx]

**Supplementary Table 2.** Summary of high-ranking fold-changes in upregulated altered proteins during ADE of DENV2-infected U937 cells

| **No.** | **NCBI ID** | **Protein** | **pI** | **MW (Da)** | **Identification score** | **No. of matched peptides** | **%cov** | **Mock (Mean±SD)** | **Treated (Mean±SD)** | **Ratio (Treated/Mock)** |
| --- | --- | --- | --- | --- | --- | --- | --- | --- | --- | --- |
| 1 | gi\|119389581 | Chain C, Ctd-Specific Phosphatase Scp1 in Complex with Peptide From C-Terminal Domain of Rna Polymerase Ii | 5.25 | 848 | 94 | 4 | 100 | no emPAI | 5.59±0 | n/a |
| 2 | gi\|190238 | Nucleolar phosphoprotein B23, partial | 9.72 | 9189 | 157 | 5 | 52.4 | no emPAI | 3.98±0 | n/a |
| 3 | gi\|190240 | Nucleolar phosphoprotein B23, partial | 9.84 | 9292 | 144 | 5 | 56.1 | 0±0 | 3.89±0 | n/a |
| 4 | gi\|119395750 | Keratin, type II cytoskeletal 1 | 8.15 | 65999 | 872 | 7 | 35.2 | 0±0 | 0.88±0 | n/a |
| 5 | gi\|578802476 | PREDICTED: protein disulfide-isomerase A6 isoform X4 | 5.79 | 53510 | 741 | 7 | 31 | no emPAI | 0.87±0.27 | n/a |
| 6 | gi\|48257068 | HSPA8 protein, partial | 5.36 | 64633 | 791 | 7 | 31.2 | 0±0 | 0.81±0.62 | n/a |
| 7 | gi\|178024 | Beta-actin, partial | 6.75 | 7794 | 133 | 4 | 52.1 | 0±0 | 0.78±0.33 | n/a |
| 8 | gi\|8569617 | Chain C, Crystal Structure of the Moesin Ferm DomainTAIL DOMAIN COMPLEX | 7 | 10341 | 101 | 4 | 33.3 | no emPAI | 0.78±0 | n/a |
| 9 | gi\|574584803 | Tubulin beta-4A chain isoform 1 | 4.92 | 54432 | 434 | 6 | 28.1 | 0±0 | 0.7±0.1 | n/a |
| 10 | gi\|6063147 | Ezrin | 9.19 | 19224 | 336 | 5 | 42.1 | no emPAI | 0.65±0.27 | n/a |
| 11 | gi\|12620919 | Prosomal P27K protein | 9.15 | 6196 | 125 | 5 | 62.1 | 0±0 | 0.58±0 | n/a |
| 12 | gi\|178027 | Alpha-actin | 5.23 | 42081 | 445 | 6 | 27.9 | 0±0 | 0.55±0.21 | n/a |
| 13 | gi\|30908859 | Actin alpha 1 skeletal muscle protein | 5.71 | 28133 | 335 | 5 | 27.2 | 0±0 | 0.48±0.26 | n/a |
| 14 | gi\|4139784 | Chain A, Canine Gdp-Ran Q69l Mutant | 7.01 | 24393 | 242 | 5 | 27.3 | 0±0 | 0.47±0 | n/a |
| 15 | gi\|187661962 | RecName: Full=Putative tubulin beta chain-like protein ENSP00000290377 | 4.77 | 41748 | 160 | 6 | 8.1 | 0±0 | 0.46±0 | n/a |
| 16 | gi\|346652078 | Chain A, Crystal Structure of P97n in Complex with the C-Terminus of Gp78 | 6.19 | 21025 | 157 | 5 | 25.1 | 0±0 | 0.46±0.11 | n/a |
| 17 | gi\|33990951 | RDX protein, partial | 9.53 | 40371 | 373 | 6 | 21.2 | 0±0 | 0.39±0.22 | n/a |

**Supplementary Table 2.** Summary of high-ranking fold-changes in upregulated altered proteins during ADE of DENV2-infected U937 cells

| **No.** | **NCBI ID** | **Protein** | **pI** | **MW (Da)** | **Identification score** | **No. of matched peptides** | **%cov** | **Mock (Mean±SD)** | **Treated (Mean±SD)** | **Ratio (Treated/Moc)** |
| --- | --- | --- | --- | --- | --- | --- | --- | --- | --- | --- |
| 18 | gi\|28436809 | Radixin | 5.88 | 68522 | 370 | 6 | 13.7 | 0±0 | 0.29±0.03 | n/a |
| 19 | gi\|14326412 | Short heat shock protein 60 Hsp60s2 | 4.62 | 27079 | 159 | 5 | 12.4 | 0±0 | 0.26±0 | n/a |
| 20 | gi\|605603744 | Chain A, Crystal Structure of the Human Mortalin (grp75) Atpase Domain in the Apo Form | 6.54 | 41340 | 211 | 5 | 21 | 0±0 | 0.26±0 | n/a |
| 21 | gi\|6063145 | Ezrin | 9.3 | 19076 | 244 | 5 | 40.5 | 0±0 | 0.25±0.09 | n/a |
| 22 | gi\|1314308 | Nucleophosmin-retinoic acid receptor alpha fusion protein NPM-RAR long form | 5.85 | 62533 | 131 | 4 | 6.9 | no emPAI | 0.23±0 | n/a |
| 23 | gi\|62088144 | Stathmin 1 variant | 8.47 | 15093 | 112 | 5 | 22.4 | 0±0 | 0.23±0 | n/a |
| 24 | gi\|38455427 | T-complex protein 1 subunit delta isoform a | 7.96 | 57888 | 307 | 5 | 22.3 | no emPAI | 0.22±0.04 | n/a |
| 25 | gi\|189238 | Neuroleukin | 8.43 | 63149 | 138 | 6 | 7.9 | 0±0 | 0.2±0.09 | n/a |
| 26 | gi\|119595805 | hCG41772, isoform CRA_c | 8.9 | 18242 | 67 | 2 | 19.3 | 0±0 | 0.19±0 | n/a |
| 27 | gi\|292162 | Heat shock protein 86, partial | 4.56 | 35652 | 188 | 6 | 14.7 | 0±0 | 0.19±0 | n/a |
| 28 | gi\|1857526 | Beta-tubulin | 5.11 | 48346 | 131 | 4 | 9.2 | 0±0 | 0.18±0.04 | n/a |
| 29 | gi\|33357460 | Chain B, Human Pyruvate Dehydrogenase | 5.95 | 37018 | 163 | 6 | 8.2 | 0±0 | 0.16±0.05 | n/a |
| 30 | gi\|2285963 | High mobility group protein 2a | 8.67 | 22902 | 87 | 3 | 15 | 0±0 | 0.15±0 | n/a |
| 31 | gi\|131412225 | Keratin, type I cytoskeletal 13 isoform a | 4.91 | 49527 | 160 | 6 | 22.7 | 0±0 | 0.14±0 | n/a |
| 32 | gi\|57997573 | Hypothetical protein | 4.71 | 27159 | 133 | 4 | 31 | 0±0 | 0.12±0 | n/a |
| 33 | gi\|119577215 | Actinin, alpha 4, isoform CRA_c | 5.24 | 104099 | 489 | 6 | 23.1 | 0±0 | 0.11±0.05 | n/a |
| 34 | gi\|350610483 | Chain A, Truncated Human Atp-Citrate Lyase with Adp and Tartrate Bound | 6.83 | 90640 | 222 | 5 | 10 | 0±0 | 0.11±0.04 | n/a |
| 35 | gi\|3157976 | Alpha actinin | 5.47 | 105159 | 431 | 6 | 19 | 0±0 | 0.11±0.03 | n/a |
| 36 | gi\|378404908 | Glyceraldehyde-3-phosphate dehydrogenase isoform 2 | 7.15 | 31528 | 117 | 5 | 20.1 | 0±0 | 0.11±0 | n/a |

**Supplementary Table 2.** Summary of high-ranking fold-changes in upregulated altered proteins during ADE of DENV2-infected U937 cells

| **No.** | **NCBI ID** | **Protein** | **pI** | **MW (Da)** | **Identification score** | **No. of matched peptides** | **%cov** | **Mock (Mean±SD)** | **Treated (Mean±SD)** | **Ratio (Treated/Mock)** |
| --- | --- | --- | --- | --- | --- | --- | --- | --- | --- | --- |
| 37 | gi\|453155 | Keratin 9 | 5.14 | 61950 | 244 | 5 | 26.8 | 0±0 | 0.11±0 | n/a |
| 38 | gi\|4557719 | DNA ligase 1 isoform 1 | 5.49 | 101673 | 157 | 5 | 6.5 | no emPAI | 0.1±0 | n/a |
| 39 | gi\|119616807 | hCG2043493, partial | 9.26 | 33954 | 111 | 4 | 8.9 | 0±0 | 0.1±0 | n/a |
| 40 | gi\|62898171 | L-plastin variant | 5.2 | 70215 | 120 | 5 | 12.4 | 0±0 | 0.1±0 | n/a |
| 41 | gi\|56967028 | Chain A, Crystal Structure of Human Dna Ligase I Bound to 5~-Adenylated, Nicked Dna | 5.79 | 76060 | 180 | 6 | 11.8 | no emPAI | 0.09±0 | n/a |
| 42 | gi\|119574084 | Guanine nucleotide binding protein (G protein), beta polypeptide 2-like 1, isoform CRA_h | 8.67 | 39680 | 72 | 2 | 5.8 | 0±0 | 0.08±0 | n/a |
| 43 | gi\|3329390 | SKD1 homolog | 6.75 | 49255 | 67 | 2 | 7.4 | no emPAI | 0.07±0 | n/a |
| 44 | gi\|119581140 | Keratin, hair, acidic, 5, isoform CRA_a | 4.85 | 50339 | 107 | 5 | 6.4 | 0±0 | 0.07±0 | n/a |
| 45 | gi\|12311759 | Type I hair keratin 8 | 4.79 | 50457 | 71 | 2 | 6.1 | 0±0 | 0.07±0 | n/a |
| 46 | gi\|186685 | Keratin type 16 | 4.97 | 50668 | 131 | 4 | 13.2 | 0±0 | 0.07±0 | n/a |
| 47 | gi\|31074643 | Type I inner root sheath specific keratin 25 irs4 | 5.33 | 50564 | 96 | 4 | 8.4 | 0±0 | 0.07±0 | n/a |
| 48 | gi\|3724107 | Type I hair keratin 5 | 4.75 | 47556 | 73 | 2 | 6.6 | 0±0 | 0.07±0 | n/a |
| 49 | gi\|4929561 | CGI-46 protein | 6.25 | 48261 | 126 | 5 | 13.1 | 0±0 | 0.07±0 | n/a |
| 50 | gi\|7108915 | Glucocorticoid receptor AF-1 specific elongation factor | 9.08 | 46240 | 124 | 5 | 12.4 | 0±0 | 0.07±0 | n/a |
| 51 | gi\|94538345 | Keratin, type I cuticular Ha5 | 4.85 | 50329 | 80 | 3 | 6.4 | 0±0 | 0.07±0 | n/a |
| 52 | gi\|12803709 | Keratin 14 | 5.09 | 51619 | 148 | 5 | 16.9 | no emPAI | 0.06±0 | n/a |
| 53 | gi\|1195531 | Type I keratin 16 | 4.99 | 51206 | 122 | 5 | 10.6 | 0±0 | 0.06±0 | n/a |
| 54 | gi\|6739602 | Talin | 5.77 | 269486 | 264 | 5 | 6.3 | 0±0 | 0.04±0 | n/a |
| 55 | gi\|116284394 | Myosin-14 isoform 2 | 5.52 | 227732 | 152 | 5 | 3.7 | 0±0 | 0.03±0.02 | n/a |
| 56 | gi\|35046 | NF-M | 4.9 | 102386 | 67 | 2 | 3.9 | 0±0 | 0.03±0 | n/a |

**Supplementary Table 2.** Summary of high-ranking fold-changes in upregulated altered proteins during ADE of DENV2-infected U937 cells

| **No.** | **NCBI ID** | **Protein** | **pI** | **MW (Da)** | **Identification score** | **No. of matched peptides** | **%cov** | **Mock (Mean±SD)** | **Treated (Mean±SD)** | **Ratio (Treated/Mock)** |
| --- | --- | --- | --- | --- | --- | --- | --- | --- | --- | --- |
| 57 | gi\|7243019 | KIAA1319 protein | 5.44 | 137518 | 80 | 3 | 2.9 | no emPAI | 0.02±0 | n/a |
| 58 | gi\|119608589 | Calmodulin regulated spectrin-associated protein 1, isoform CRA_a | 6.27 | 160293 | 103 | 4 | 2.8 | 0±0 | 0.02±0 | n/a |
| 59 | gi\|825671 | B23 nucleophosmin (280 AA) | 4.71 | 30919 | 247 | 5 | 24.6 | 0.4±0.07 | 1.5±0 | 3.69 |
| 60 | gi\|307086 | Keratin-10 | 5.11 | 46359 | 350 | 6 | 31.6 | 0.15±0 | 0.51±0 | 3.40 |
| 61 | gi\|119581085 | Keratin 10 (epidermolytic hyperkeratosis; keratosis palmaris et plantaris), isoform CRA_b | 5.13 | 63308 | 509 | 6 | 33.8 | 0.16±0 | 0.5±0 | 3.13 |
| 62 | gi\|623409 | Keratin 10 | 5.01 | 57213 | 390 | 6 | 30.5 | 0.18±0 | 0.4±0 | 2.22 |
| 63 | gi\|181402 | Epidermal cytokeratin 2 | 8.07 | 65825 | 417 | 6 | 25.7 | 0.1±0 | 0.22±0 | 2.20 |
| 64 | gi\|119617032 | Keratin 6B, isoform CRA_a | 8.38 | 59874 | 222 | 5 | 12.4 | 0.05±0 | 0.11±0 | 2.20 |
| 65 | gi\|8394076 | Proteasome subunit alpha type-6 [Rattus norvegicus] | 6.34 | 27382 | 212 | 5 | 30.1 | 0.12±0 | 0.26±0 | 2.17 |
| 66 | gi\|13124875 | Myosin-11 isoform SM2A | 5.44 | 223439 | 221 | 5 | 4.3 | 0.02±0.01 | 0.04±0.02 | 2.00 |
| 67 | gi\|6005942 | Transitional endoplasmic reticulum ATPase | 5.14 | 89266 | 581 | 6 | 21.1 | 0.23±0.11 | 0.46±0.13 | 2.00 |
| 68 | gi\|5453603 | T-complex protein 1 subunit beta isoform 1 | 6.01 | 57452 | 444 | 6 | 37.4 | 0.22±0.04 | 0.4±0.2 | 1.86 |
| 69 | gi\|119590106 | hCG27371 | 9.11 | 9203 | 114 | 4 | 28.6 | 0.9±0 | 1.62±0 | 1.80 |
| 70 | gi\|11935049 | Keratin 1 | 8.16 | 66027 | 868 | 7 | 35.2 | 0.52±0.07 | 0.88±0 | 1.69 |
| 71 | gi\|34740335 | Tubulin alpha-1B chain [Mus musculus] | 4.94 | 50120 | 487 | 6 | 29.9 | 0.47±0.33 | 0.77±0 | 1.63 |
| 72 | gi\|386854 | Type II keratin subunit protein, partial | 5.31 | 52757 | 461 | 6 | 26.8 | 0.33±0.08 | 0.53±0 | 1.62 |
| 73 | gi\|401664164 | EZR-ROS1 fusion protein | 5.8 | 98886 | 380 | 6 | 9.9 | 0.12±0.05 | 0.18±0.09 | 1.54 |
| 74 | gi\|4503483 | Elongation factor 2 | 6.41 | 95277 | 547 | 6 | 19.8 | 0.17±0.11 | 0.27±0.05 | 1.53 |

**Supplementary Table 2.** Summary of high-ranking fold-changes in upregulated altered proteins during ADE of DENV2-infected U937 cells

| **No.** | **NCBI ID** | **Protein** | **pI** | **MW (Da)** | **Identification score** | **No. of matched peptides** | **%cov** | **Mock (Mean±SD)** | **Treated (Mean±SD)** | **Ratio (Treated/Mock)** |
| --- | --- | --- | --- | --- | --- | --- | --- | --- | --- | --- |
| 75 | gi\|375314779 | Keratin 1 | 8.15 | 66026 | 820 | 7 | 35.2 | 0.52±0.07 | 0.79±0 | 1.52 |
| 76 | gi\|441674116 | PREDICTED: LOW QUALITY PROTEIN: moesin [Nomascus leucogenys] | 5.98 | 67936 | 1146 | 8 | 42.7 | 0.99±0.14 | 1.45±0 | 1.46 |
| 77 | gi\|4503481 | Elongation factor 1-gamma | 6.25 | 50087 | 374 | 6 | 25.6 | 0.35±0.15 | 0.49±0.25 | 1.40 |
| 78 | gi\|426352367 | PREDICTED: spliceosome RNA helicase DDX39B isoform 3 [Gorilla gorilla gorilla] | 8.93 | 40551 | 133 | 4 | 18.6 | 0.13±0.05 | 0.17±0.09 | 1.36 |
| 79 | gi\|74722492 | RecName: Full=Putative heat shock protein HSP 90-beta-3; AltName: Full=Heat shock protein 90-beta c; Short=Heat shock protein 90Bc | 4.71 | 68282 | 357 | 6 | 18.3 | 0.16±0.06 | 0.21±0.06 | 1.32 |
| 80 | gi\|4507677 | Endoplasmin precursor | 4.76 | 92411 | 575 | 6 | 16.9 | 0.31±0.16 | 0.4±0.03 | 1.27 |

NCBI = National center for Biotechnology Information. %Cov. = %Sequence covage [(number of the mathched residues/total number of residues in the entire sequence) x 100%]. DIV/0 = Divide by zero. emPAI = Exponentially modified protein abundance index. n/a = not available.
