# Supplementary material for: Protein Disulfide Isomerase Inhibitor Suppresses Viral Replication and Production during Antibody-Dependent Enhancement of Dengue Virus Infection in Human Monocytic Cells
Source: Viruses. 2019 Feb 13;11(2):155. doi: 10.3390/v11020155 (PMC6410196; doi:10.3390/v11020155)
Supplement: Supplementary file 1 [file viruses-11-00155-s001.zip › Supplementary Table 3 Nov 2018.docx]

**Supplementary Table 3.** Summary of high-ranking fold-changes in downregulated altered proteins during ADE of DENV2-infected U937 cells

| **No.** | **NCBI ID** | **Protein** | **pI** | **MW (Da)** | **Identification score** | **No. of matched peptides** | **%cov** | **Mock (Mean±SD)** | **Treated (Mean±SD)** | **Ratio (Treated/Mock)** |
| --- | --- | --- | --- | --- | --- | --- | --- | --- | --- | --- |
| 1 | gi\|159164226 | Chain A, The Solution Structure of The Second Thioredoxin Domain of Human Protein Disulfide-Isomerase A3 | 5.27 | 15669 | 438 | 6 | 50.7 | 2.81±1.42 | 0±0 | 0.00 |
| 2 | gi\|538260950 | Chain A, Crystal Structure of A0-domain Of P5 From H. Sapiens | 5.88 | 12786 | 240 | 5 | 46.6 | 1.04±0 | 0±0 | 0.00 |
| 3 | gi\|119597758 | dUTP pyrophosphatase, isoform CRA_d | 9.88 | 23028 | 260 | 5 | 31.7 | 0.89±0.12 | 0±0 | 0.00 |
| 4 | gi\|4501881 | Actin, alpha skeletal muscle | 5.23 | 42024 | 542 | 6 | 30.2 | 0.71±0.21 | 0±0 | 0.00 |
| 5 | gi\|4501889 | Actin, gamma-enteric smooth muscle isoform 1 precursor | 5.31 | 41850 | 532 | 6 | 29.3 | 0.71±0.21 | 0±0 | 0.00 |
| 6 | gi\|14625824 | Moesin/anaplastic lymphoma kinase fusion protein | 7.61 | 61833 | 703 | 7 | 27.9 | 0.61±0.35 | 0±0 | 0.00 |
| 7 | gi\|119630329 | Chaperonin containing TCP1, subunit 8 (theta), isoform CRA_a | 5.54 | 59440 | 510 | 6 | 33.5 | 0.5±0.12 | 0±0 | 0.00 |
| 8 | gi\|38014278 | TUBB3 protein, partial | 4.9 | 45593 | 365 | 6 | 26.8 | 0.47±0.05 | 0±0 | 0.00 |
| 9 | gi\|6470150 | BiP protein, partial | 5.23 | 70888 | 562 | 6 | 26.9 | 0.31±0.06 | 0±0 | 0.00 |
| 10 | gi\|193244897 | Beta globin | 6.17 | 11479 | 89 | 3 | 26.7 | 0.3±0 | 0±0 | 0.00 |
| 11 | gi\|4502643 | T-complex protein 1 subunit zeta isoform a | 6.23 | 57988 | 304 | 5 | 21.1 | 0.29±0.03 | 0±0 | 0.00 |
| 12 | gi\|1800303 | HIV-1 Nef interacting protein, partial | 6.92 | 45205 | 200 | 5 | 20.6 | 0.29±.0.04 | 0±0 | 0.00 |
| 13 | gi\|296080693 | Glucose-6-phosphate isomerase isoform 1 | 9.01 | 64284 | 302 | 5 | 18.1 | 0.23±13 | 0±0 | 0.00 |
| 14 | gi\|530417302 | PREDICTED: alpha-actinin-4 isoform X1 | 5.31 | 104260 | 581 | 6 | 24.3 | 0.23±0.05 | 0±0 | 0.00 |
| 15 | gi\|15214751 | EEF1A1 protein, partial | 9.56 | 17033 | 97 | 4 | 13 | 0.2±0 | 0±0 | 0.00 |
| 16 | gi\|9507215 | Tubulin alpha-8 chain isoform 1 | 4.94 | 50062 | 139 | 6 | 12 | 0.18±0.04 | 0±0 | 0.00 |
| 17 | gi\|46249758 | Ezrin | 5.94 | 69199 | 370 | 6 | 13.5 | 0.17±0.07 | 0±0 | 0.00 |
| 18 | gi\|386781550 | Radixin isoform 1 | 6.3 | 71005 | 336 | 5 | 10.3 | 0.16±0.07 | 0±0 | 0.00 |

**Supplementary Table 3.** Summary of high-ranking fold-changes in downregulated altered proteins during ADE of DENV2-infected U937 cells

| **No.** | **NCBI ID** | **Protein** | **pI** | **MW (Da)** | **Identification score** | **No. of matched peptides** | **%cov** | **Mock (Mean±SD)** | **Treated (Mean±SD)** | **Ratio (Treated/Mock)** |
| --- | --- | --- | --- | --- | --- | --- | --- | --- | --- | --- |
| 19 | gi\|609342 | Nucleophosmin-anaplastic lymphoma kinase fusion protein | 6.44 | 75266 | 120 | 5 | 10.4 | 0.16±0.02 | 0±0 | 0.00 |
| 20 | gi\|24119203 | Tropomyosin alpha-3 chain isoform Tpm3.1cy | 4.75 | 29015 | 166 | 6 | 27.8 | 0.15±0.06 | 0±0 | 0.00 |
| 21 | gi\|167887670 | Proteasome subunit alpha type 7-like protein variant 1 | 9.14 | 23789 | 84 | 3 | 18.9 | 0.14±0 | 0±0 | 0.00 |
| 22 | gi\|62897075 | Heat shock 70kDa protein 9B precursor variant | 5.87 | 73589 | 317 | 5 | 18.7 | 0.12±0.08 | 0±0 | 0.00 |
| 23 | gi\|119576470 | hCG2010471 | 7.7 | 32925 | 78 | 2 | 5.2 | 0.1±0 | 0±0 | 0.00 |
| 24 | gi\|167614506 | Plastin-2 | 5.29 | 70244 | 197 | 5 | 9.9 | 0.1±0 | 0±0 | 0.00 |
| 25 | gi\|9230777 | LIM protein ACT | 7.77 | 32791 | 73 | 2 | 18.3 | 0.1±0 | 0±0 | 0.00 |
| 26 | gi\|62089222 | Heat shock 70kDa protein 1A variant | 5.97 | 77448 | 298 | 5 | 18.9 | 0.09±0.04 | 0±0 | 0.00 |
| 27 | gi\|158518381 | RecName: Full=Putative heat shock 70 kDa protein 7; AltName: Full=Heat shock 70 kDa protein B | 7.72 | 40220 | 128 | 5 | 7.9 | 0.08±0 | 0±0 | 0.00 |
| 28 | gi\|206725454 | Phosphatidylinositol 4-phosphate 5-kinase-like protein 1 isoform 1 | 9.63 | 44544 | 83 | 3 | 7.4 | 0.07±0 | 0±0 | 0.00 |
| 29 | gi\|578817058 | PREDICTED: phosphatidylinositol 4-phosphate 5-kinase-like protein 1 isoform X1 | 9.48 | 53306 | 95 | 4 | 8 | 0.06±0 | 0±0 | 0.00 |
| 30 | gi\|88900491 | Neutral alpha-glucosidase AB isoform 3 precursor | 5.82 | 109369 | 164 | 6 | 10.4 | 0.06±0 | 0±0 | 0.00 |
| 31 | gi\|194018511 | Keratin, type II cytoskeletal 1b | 5.73 | 61864 | 108 | 4 | 11.4 | 0.05±0 | 0±0 | 0.00 |
| 32 | gi\|12666531 | Putative b,b-carotene-9~,10~-dioxygenase | 8.11 | 62842 | 75 | 2 | 3.6 | 0.05±0 | 0±0 | 0.00 |
| 33 | gi\|10863945 | X-ray repair cross-complementing protein 5 | 5.55 | 82652 | 73 | 2 | 2.3 | 0.04±0 | 0±0 | 0.00 |
| 34 | gi\|38196957 | FASN protein, partial | 6.22 | 84158 | 111 | 4 | 4.9 | 0.04±0 | 0±0 | 0.00 |
| 35 | gi\|33438760 | Myosin heavy chain | 5.76 | 227863 | 247 | 5 | 5.2 | 0.03±0.02 | 0±0 | 0.00 |

**Supplementary Table 3.** Summary of high-ranking fold-changes in downregulated altered proteins during ADE of DENV2-infected U937 cells

| **No.** | **NCBI ID** | **Protein** | **pI** | **MW (Da)** | **Identification score** | **No. of matched peptides** | **%cov** | **Mock (Mean±SD)** | **Treated (Mean±SD)** | **Ratio (Treated/Mock)** |
| --- | --- | --- | --- | --- | --- | --- | --- | --- | --- | --- |
| 36 | gi\|20521736 | KIAA1027 protein | 5.8 | 270596 | 257 | 5 | 7.1 | 0.03±0.01 | 0±0 | 0.00 |
| 37 | gi\|4235275 | Talin | 5.75 | 269661 | 252 | 5 | 6.7 | 0.03±0.01 | 0±0 | 0.00 |
| 38 | gi\|178058 | Alpha-actinin | 5.4 | 103229 | 143 | 5 | 4.7 | 0.03±0 | 0±0 | 0.00 |
| 39 | gi\|288562972 | Chain A, Structure of The Human Fatty Acid Synthase Ks-Mat Didomain As A Framework For Inhibitor Design | 5.82 | 104302 | 102 | 4 | 5.3 | 0.03±0 | 0±0 | 0.00 |
| 40 | gi\|393007760 | SND1-BRAF fusion | 9.36 | 109079 | 90 | 3 | 8.7 | 0.03±0 | 0±0 | 0.00 |
| 41 | gi\|4579911 | apg-1 | 5.65 | 94446 | 142 | 5 | 9.4 | 0.03±0 | 0±0 | 0.00 |
| 42 | gi\|459352730 | POTE ankyrin domain family member J | 5.66 | 117315 | 126 | 5 | 4.4 | 0.03±0 | 0±0 | 0.00 |
| 43 | gi\|77404397 | staphylococcal nuclease domain-containing protein 1 | 6.74 | 101934 | 110 | 4 | 9 | 0.03±0 | 0±0 | 0.00 |
| 44 | gi\|799177 | 100 kDa coactivator | 6.62 | 99628 | 95 | 4 | 8 | 0.03±0 | 0±0 | 0.00 |
| 45 | gi\|30026465 | pol protein [Human endogenous retrovirus HCML-ARV] | 9.22 | 131387 | 70 | 2 | 3.9 | 0.02±0 | 0±0 | 0.00 |
| 46 | gi\|530405585 | PREDICTED: dmX-like protein 2 isoform X3 | 5.88 | 291483 | 84 | 3 | 2.1 | 0.01±0 | 0±0 | 0.00 |
| 47 | gi\|27451602 | Tau-tubulin kinase | 6.58 | 182353 | 112 | 5 | 4.1 | 0.02±0 | no emPAI | n/a |
| 48 | gi\|4758282 | Ephrin type-A receptor 7 isoform 1 precursor | 5.58 | 112024 | 95 | 4 | 6.3 | 0.03±0.01 | no emPAI | n/a |
| 49 | gi\|117606360 | PH and SEC7 domain-containing protein 3 Isoform a | 5.68 | 115863 | 98 | 3 | 5.4 | 0.03±0 | no emPAI | n/a |
| 50 | gi\|62087882 | Heat shock 70kDa protein 4 isoform a variant | 5.44 | 87949 | 76 | 2 | 5.4 | 0.04±0 | no emPAI | n/a |
| 51 | gi\|4507241 | FACT complex subunit SSRP1 | 6.45 | 81024 | 87 | 3 | 7.5 | 0.05±0.02 | no emPAI | n/a |
| 52 | gi\|68533125 | ACLY variant protein | 8.24 | 124477 | 328 | 5 | 12.5 | 0.09±0.06 | no emPAI | n/a |
| 53 | gi\|260268505 | FLJ00343 protein | 5.77 | 281288 | 1018 | 8 | 17.6 | 0.12±0.02 | no emPAI | n/a |

**Supplementary Table 3.** Summary of high-ranking fold-changes in downregulated altered proteins during ADE of DENV2-infected U937 cells

| **No.** | **NCBI ID** | **Protein** | **pI** | **MW (Da)** | **Identification score** | **No. of matched peptides** | **%cov** | **Mock (Mean±SD)** | **Treated (Mean±SD)** | **Ratio (Treated/Mock)** |
| --- | --- | --- | --- | --- | --- | --- | --- | --- | --- | --- |
| 54 | gi\|190613719 | Chain B, Crystal Structure of A Complex of Sse1p And Hsp70, Selenomethionine- Labeled Crystals | 6.4 | 41904 | 259 | 5 | 26.4 | 0.13±0.04 | no emPAI | n/a |
| 55 | gi\|426331707 | PREDICTED: tropomyosin alpha-3 chain isoform 6 [Gorilla gorilla gorilla] | 4.73 | 33202 | 120 | 5 | 14 | 0.14±0.05 | no emPAI | n/a |
| 56 | gi\|13529302 | GANAB protein, partial | 6.78 | 34558 | 129 | 4 | 16 | 0.15±0.05 | no emPAI | n/a |
| 57 | gi\|3712663 | DEAD-box protein | 6.37 | 33095 | 131 | 4 | 17.3 | 0.16±0.06 | no emPAI | n/a |
| 58 | gi\|292659561 | Chain A, Crystal Structure of The Complex Between The Bag5 Bd5 And Hsp70 Nbd | 6.38 | 43068 | 180 | 6 | 16.8 | 0.16±0 | no emPAI | n/a |
| 59 | gi\|190447 | Prosomal protein P30-33K | 6.51 | 30208 | 127 | 5 | 15.2 | 0.17±0.06 | no emPAI | n/a |
| 60 | gi\|4204880 | Heat shock protein | 5.56 | 69952 | 335 | 5 | 15.2 | 0.23±0.14 | no emPAI | n/a |
| 61 | gi\|197115528 | Immunoglobulin heavy chain variable region | 8.96 | 12966 | 121 | 5 | 28.9 | 0.26±0 | no emPAI | n/a |
| 62 | gi\|222476525 | 60 kDa chaperonin | 7.93 | 19902 | 79 | 2 | 15.2 | 0.27±0.1 | no emPAI | n/a |
| 63 | gi\|671527 | Gamma subunit of CCT chaperonin | 6.23 | 60292 | 485 | 6 | 28.1 | 0.44±0.24 | no emPAI | n/a |
| 64 | gi\|178045 | Gamma-actin, partial | 5.65 | 25862 | 214 | 5 | 20.7 | 0.45±0.18 | no emPAI | n/a |
| 65 | gi\|6457378 | Cytovillin 2 | 9.32 | 16237 | 326 | 5 | 49.6 | 0.56±0.15 | no emPAI | n/a |
| 66 | gi\|62421170 | Actin-like protein | 6.06 | 11549 | 96 | 4 | 24.3 | 0.5±0.2 | no emPAI | n/a |
| 67 | gi\|31645 | Glyceraldehyde-3-phosphate dehydrogenase | 8.26 | 36031 | 117 | 5 | 13.7 | 0.44±0.25 | 0.09±0 | 0.20 |
| 68 | gi\|4503423 | Deoxyuridine 5~-triphosphate Nucleotidohydrolase, mitochondrial isoform 2 | 6.15 | 17737 | 299 | 5 | 62.2 | 1.87±0.4 | 0.98±0.62 | 0.53 |
| 69 | gi\|5453549 | Peroxiredoxin-4 precursor | 5.86 | 30521 | 188 | 6 | 18.1 | 0.36±0 | 0.19±0.06 | 0.53 |
| 70 | gi\|4503143 | Cathepsin D preproprotein | 6.1 | 44524 | 116 | 5 | 15.3 | 0.27±0.04 | 0.15±0 | 0.56 |
| 71 | gi\|62088022 | Hypothetical protein FLJ41407 variant | 8.54 | 59576 | 198 | 5 | 8.8 | 0.24±0.07 | 0.14±0.03 | 0.58 |

**Supplementary Table 3.** Summary of high-ranking fold-changes in downregulated altered proteins during ADE of DENV2-infected U937 cells

| **No.** | **NCBI ID** | **Protein** | **pI** | **MW (Da)** | **Identification score** | **No. of matched peptides** | **%cov** | **Mock (Mean±SD)** | **Treated (Mean±SD)** | **Ratio (Treated/Mock)** |
| --- | --- | --- | --- | --- | --- | --- | --- | --- | --- | --- |
| 72 | gi\|4529892 | HSP70-2 | 5.48 | 69982 | 202 | 5 | 12.9 | 0.13±0.03 | 0.08±0.03 | 0.60 |
| 73 | gi\|5902134 | Coronin-1A | 6.25 | 50994 | 227 | 5 | 9.8 | 0.37±0.09 | 0.25±0.04 | 0.66 |
| 74 | gi\|5803187 | Transaldolase | 6.36 | 37516 | 282 | 5 | 29.4 | 0.77±0.47 | 0.53±0.13 | 0.69 |
| 75 | gi\|2443580 | dUTPase | 9.65 | 26690 | 283 | 5 | 40.1 | 1.04±0.2 | 0.73±0.31 | 0.70 |
| 76 | gi\|5729877 | Heat shock cognate 71 kDa protein isoform 1 | 5.37 | 70854 | 986 | 7 | 33.7 | 1.33±0.27 | 0.95±0.67 | 0.72 |
| 77 | gi\|159162689 | Chain A, Human Protein Disulfide Isomerase, Nmr, 40 Structures | 5.94 | 13249 | 433 | 6 | 70.8 | 6.13±4.15 | 4.66±3.3 | 0.76 |
| 78 | gi\|12667788 | Myosin-9 | 5.5 | 226392 | 1008 | 8 | 15.7 | 0.32±0.22 | 0.25±0.17 | 0.76 |
| 79 | gi\|5729953 | Nuclear migration protein nudC | 5.27 | 38219 | 165 | 6 | 12.1 | 0.19±0.1 | 0.15±0.04 | 0.81 |
| 80 | gi\|114794262 | Chain A, Crystal Structure of The Bb~ Fragment of Erp57 | 5.49 | 28426 | 316 | 5 | 33.7 | 0.99±0.62 | 0.83±0.33 | 0.84 |

NCBI = National center for Biotechnology Information. %Cov. = %Sequence covage [(number of the mathched residues/total number of residues in the entire sequence) x 100%]. DIV/0 = Divide by zero. emPAI = Exponentially modified protein abundance index. n/a = not available.
